# B(C_6_F_5_)_3_-catalyzed oxidation of α-diazoesters using DMF and molecular oxygen as oxygen sources†

**DOI:** 10.1039/d2ra05739e

**Published:** 2022-11-23

**Authors:** Bei Wang, Guo-Min Zhang, Hua Zhang, Ji-Yu Wang

**Affiliations:** Department of Chemistry, Asymmetric Synthesis and Chiral Technology Key Laboratory of Sichuan Province, Xihua University Chengdu 610041 P. R. China; Chengdu Institute of Organic Chemistry, Chinese Academy of Sciences Chengdu 610041 P. R. China; University of Chinese Academy of Sciences Beijing 100049 P. R. China

## Abstract

A metal-free catalytic oxidation of α-diazoesters *via* a green environmental-friendly route was developed. The α-diazoesters were converted to α-ketoesters using DMF and molecular oxygen as oxygen sources and B(C_6_F_5_)_3_ as the catalyst, without any additives. This protocol has a broad adaptability of substrates and good compatibility with a range of functional groups, and it offers new insight into reactions catalyzed by B(C_6_F_5_)_3_.

## Abstract 2

B(C_6_F_5_)_3_-catalyzed synthesis of α-ketoesters *via* the oxidation of α-diazoesters using DMF and molecular oxygen as oxygen sources..

## Graphical abstract

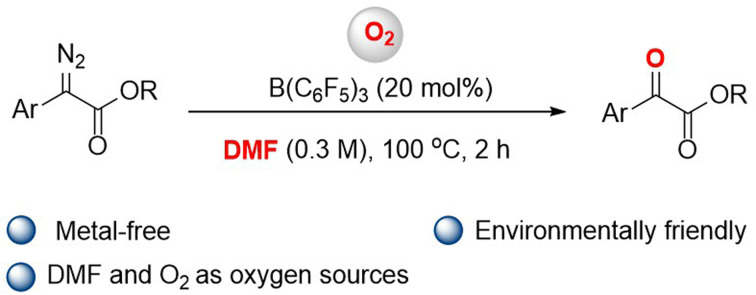

## Introduction

**Fig. 1:**
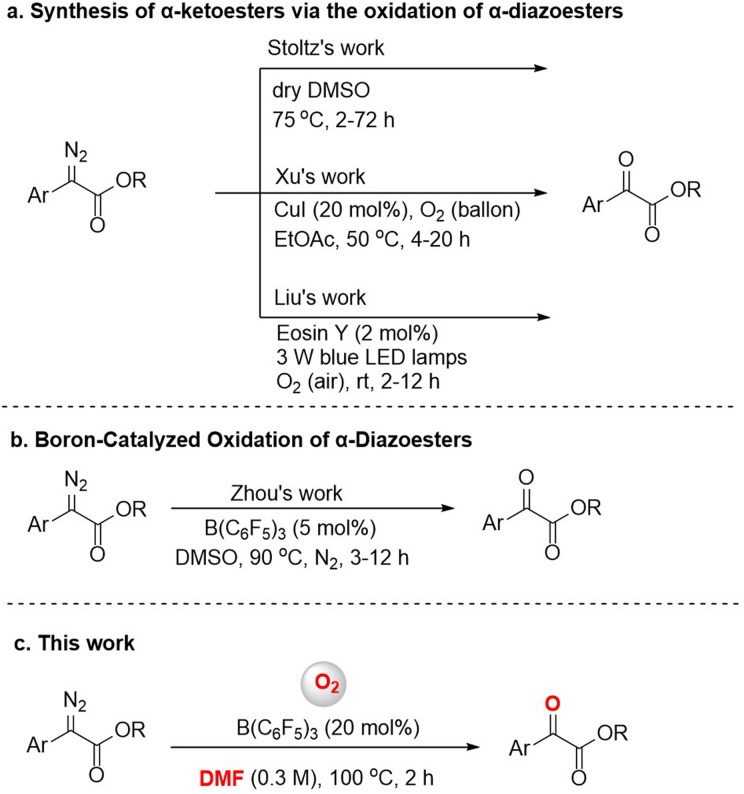
The oxidation reactions of α-diazoesters.

α-Diazoesters are an important class of organic synthesis synthon.^1^ Diazo compounds can form metal carbenes by transition metal catalysis, and the reactions of insertion or cyclopropanation based on metal carbines have been developed in the past few decades.^2^ The oxidation reaction of α-diazoesters can generate α-ketoesters, which usually have biological activity, and can also transform into a variety of functional groups.^3^ Various methods for this oxidation have been reported, although many require the use of harsh oxidants like dimethyldioxirane (DMDO) or *t*-BuOCl,^4^ or expensive transition metal catalysts like Rh.^5^ In 2016, Stoltz's group found that dimethyl sulfoxide (DMSO) could also serve as an oxidant to achieve oxidation of aryl α-diazoesters (Fig. 1a).^6^ But the method applied only to the electron-rich diazo compounds. According to green and sustainable chemistry principles, molecular oxygen is considered to be an ideal oxidant due to its natural, inexpensive, and environmentally friendly nature, and therefore it has attractive academic and industrial prospects.^7^ The reactions of preformed stable metal carbene compounds with oxygen are known.^8^ In 2021, Xu *et al.* reported a highly efficient and catalytic procedure for the aerobic oxidation of α-diazoesters to α-ketoesters *via* a copper carbene intermediate (Fig. 1a).^9^ In addition, Liu *et al.* used cheap, readily available Eosin Y as a photocatalyst and O_2_ (air) as a green oxidant, and achieved the aerobic oxidation of α-diazoesters under visible light in air at room temperature (Fig. 1a).^10^ Research on the aerobic oxidation of α-diazoesters especially by non-metal catalysts still has space for exploration.

In recent years, tris(pentafluorophenyl)borane has been widely used in the reduction or addition reactions of unsaturated compounds, and in other reactions.^11^ However, few efforts have been devoted to B(C_6_F_5_)_3_-catalyzed oxidation reactions. For instance, the borane-mediated hydride abstraction of amines results in the generation of reactive iminium hydridoborate salts that participate in a variety of stoichiometric and catalytic processes.^12^ In 2021, Basak *et al.* provided a detailed overview of the borane-mediated dehydrogenation functionalization of amine compounds.^13^ Moreover, in 2017, Babu's group reported an efficient one-pot oxidative esterification and amidation of aldehydes using B(C_6_F_5_)_3_ as the catalyst and TBHP as the oxidant.^14^ In 2018, Ling *et al.* reported a B(C_6_F_5_)_3_-catalyzed oxidative deamination/cyclization cascade reaction of benzyl amines and ketones for the synthesis of 2,4,6-triarylpyridines in an oxygen atmosphere.^15^ In addition, several reports have been published on the B(C_6_F_5_)_3_-catalyzed activation of diazo compounds;^16^ for example, Tang's group reported a boron-catalyzed O–H bond insertion of α-aryl α-diazoesters in water.^17^ In previous works, our group has also conducted research on B(C_6_F_5_)_3_-catalyzed reactions and achieved some results.^18^ During our research, we found that B(C_6_F_5_)_3_ could catalyze the oxidation of α-diazoesters under an O_2_ atmosphere. Subsequently, Zhou's group described a B(C_6_F_5_)_3_-catalyzed oxidation reaction of α-diazoesters with DMSO as an oxygen source (Fig. 1b).^19^ But, different to this work, we report a B(C_6_F_5_)_3_-catalyzed oxidation of α-diazoesters to obtain α-ketoesters by activating O_2_ directly (Fig. 1c). And this protocol features a wide substrate scope including aromatic heterocycle α-diazoesters and good functional group tolerance.

## Results and discussion

The ethyl phenyldiazoacetate 1a was selected as the model substrate to investigate the oxidation reaction under various reaction conditions (Table 1). Initially, the reaction was carried out using different catalysts at 100 °C for 2 hours in DMF in an oxygen atmosphere. First, only a small amount of oxidation product 2-1a was obtained without any catalyst (entry 1). To our delight, B(C_6_F_5_)_3_ catalyzed the oxidation reaction and the yield of 2-1a increased to 57% (entry 2). 2,4,6-BAr^F^, 3,4,5-BAr^F^, Cu(OAc)_2_ and CoCl_2_ also had certain catalytic effects on the reaction, but the results were not as good as those with B(C_6_F_5_)_3_ (entries 3–6). Furthermore, the catalytic effects of metal Lewis acid catalysts with weaker acidity such as La(OTf)_3_ or Sc(OTf)_3_ were worse (entries 7 and 8). The byproduct ethyl mandelate was reduced when using dry DMF as solvent (entry 9). Next, we tried to increase the amount of catalyst. When the amount of catalyst was increased to 20 mol%, the yield of 2-1a was 86% (entries 10–12). Then, the temperature was adjusted to 80 °C, and the yield of the expected product decreased significantly (entry 13). Further screening of solvents showed that the type of solvent had effects on the reaction. When the reaction was performed in CH_3_CN, THF or 1, 4-dioxane, the yields of 2-1a were very low (entries 14–16). Finally, the best conditions for the reaction were determined: the reaction was run with 1a (0.3 mmol) and B(C_6_F_5_)_3_ (20 mol%) under an oxygen atmosphere (balloon) in 1 mL of dry DMF at 100 °C for 2 hours.

**Table 1:** Optimization of reaction conditions^a^^,^^b^

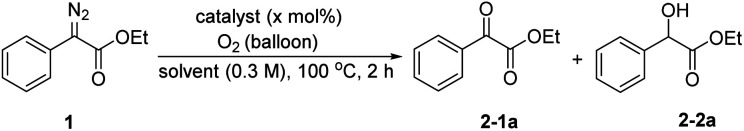					
Entry	Catalyst	Solvent	Catalyst (*x* mol%)	2-1a (%)	2-2a (%)
1	—	DMF	5	5	Trace
2	B(C_6_F_5_)_3_	DMF	5	57	20
3	2,4,6-BAr^F^	DMF	5	50	18
4	3,4,5-BAr^F^	DMF	5	47	22
5	Cu(OAc)_2_	DMF	5	42	18
6	CoCl_2_	DMF	5	39	20
7	La(OTf)_3_	DMF	5	20	25
8	Sc(OTf)_3_	DMF	5	24	23
9^c^	B(C_6_F_5_)_3_	DMF	5	68	7
10^c^	B(C_6_F_5_)_3_	DMF	10	63	4
11^c^	B(C_6_F_5_)_3_	DMF	15	70	6
12^c^	B(C_6_F_5_)_3_	DMF	20	86	1
13^c^^,^^d^	B(C_6_F_5_)_3_	DMF	20	52	7
14^c^	B(C_6_F_5_)_3_	CH_3_CN	20	15	12
15^c^	B(C_6_F_5_)_3_	THF	20	19	13
16^c^	B(C_6_F_5_)_3_	1,4-Dioxane	20	17	16

a Reaction conditions: 1a (54.0 mg, 0.3 mmol) and catalyst (*x* mol%) in solvent (1 mL) for 2 h at *T* °C.

b Yield of GC.

c The dry solvent.

d The reaction temperature was 80 °C.

**Scheme 1:**
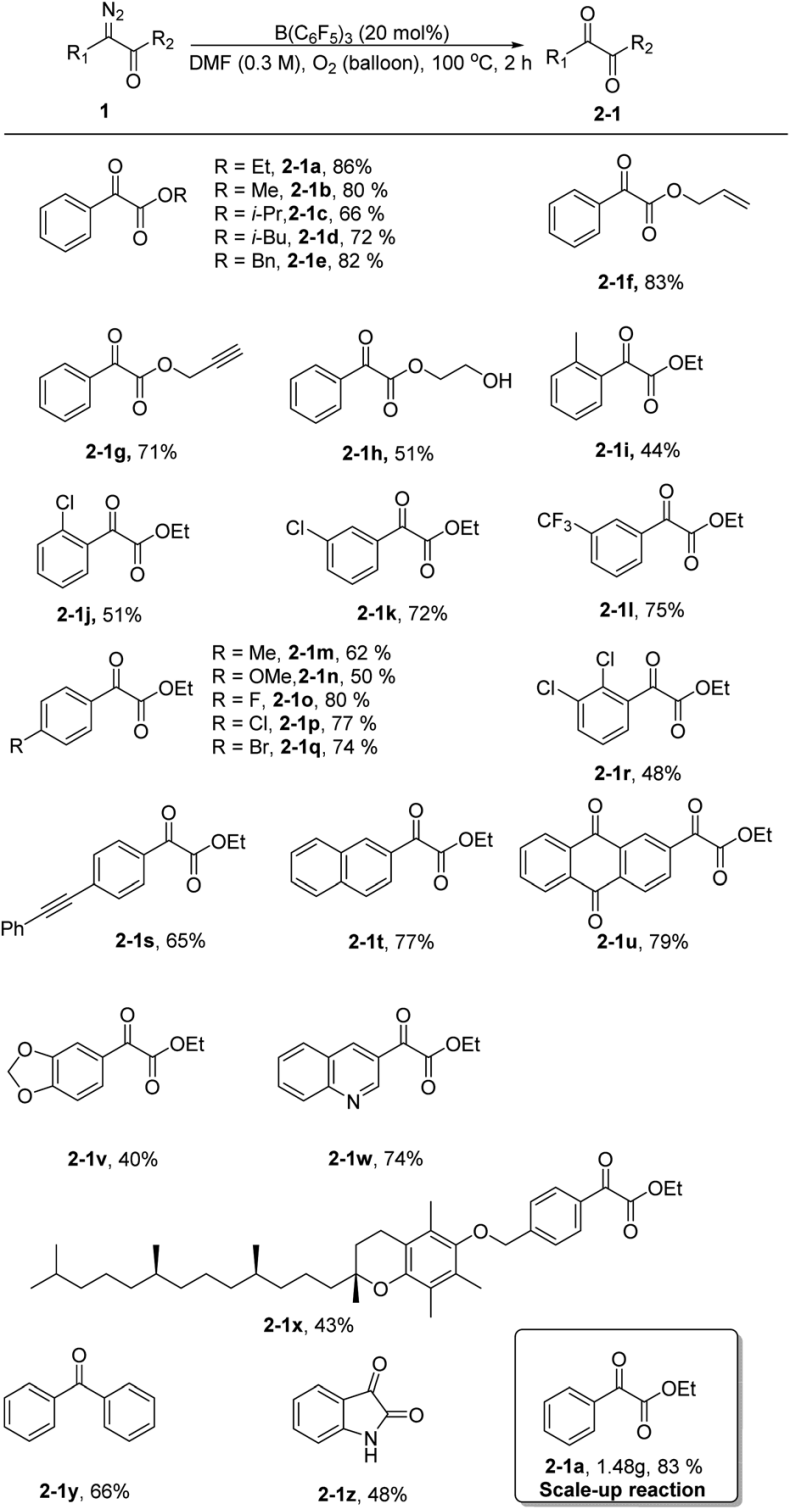
Substrate scope for α-diazoesters. Reaction conditions: 1 (0.3 mmol, 1 equiv.), B(C_6_F_5_)_3_ (20 mol%), and DMF (1 mL) at 100 °C for 2 h. Yield of isolated product. Scale-up reaction conditions: 1 (10 mmol, 1.9 g, 1 equiv.), B(C_6_F_5_)_3_ (20 mol%), and DMF (30 mL) at 100 °C for 2 h. Yield of isolated product.

Next, using the optimized conditions, the applicability of the protocol for a broad range of substrates was explored, as shown in Scheme 1. Generally, phenyl α-diazoesters with alkyl, benzyl, allyl, or propargyl substituents at R_2_ all afforded moderate to high yields of the desired α-ketoesters (2-1a–2-1h). The position and electrical properties of the substituents on the benzene ring have a great influence on the reaction. With substituents at the *ortho* position, the yield of products substantially declined, and the decrease is more obvious when the methyl group was substituted (2-1i–2-1j). This is probably due to steric hindrance and the donating property of the group, because the carbene intermediate that formed during the reaction was unstable. The yield of the products was moderate when a *meta* substituent was located on the benzene ring (2-1k–2-1l). The yield of the products was lower when the benzene ring had an electron donating substituent at the *para* position (2-1m–2-1n), and the yield of the products was moderate to good with halogen substituents (2-1o–2-1q). Multisubstituted aryl α-diazoesters such as ethyl 2-diazo-2-(2,3-dichlorophenyl)acetate could be converted to the corresponding oxidation product 2-1r with a yield of 48%. Unsaturated substituents, such as alkyne groups, also tolerated the reaction conditions (2-1s). In the case of polycyclic substrates, such as naphthalene and 2-anthraquinone, the desired products 2-1t and 2-1u could also be formed in good yields; the oxidation product of ethyl-2-(benzo[*d*][1,3]dioxol-5-yl)-2-diazoacetate could be obtained with a yield of 40% (2-1v). In addition, the substrate bearing a quinoline moiety also gave the corresponding product 2-1w, albeit in a slightly diminished yield. Aryl α-diazoesters containing tocopherol moieties could also smoothly undergo the reaction to give the corresponding product with a yield of 43% (2-1x). Finally, diphenyl–diazomethane was converted to benzophenone in a moderate yield (2-1y) and an oxindole scaffold was compatible with the reaction, allowing for the formation of isatin in 48% yield (2-1z).

Subsequently, we increased the amount of ethyl phenyldiazoacetate 1a to 10 mmol and the yield of the product 2-1a did not decrease remarkably, demonstrating that the reaction has potential for industrial scale-up (Scheme 1).

**Fig. 2:**
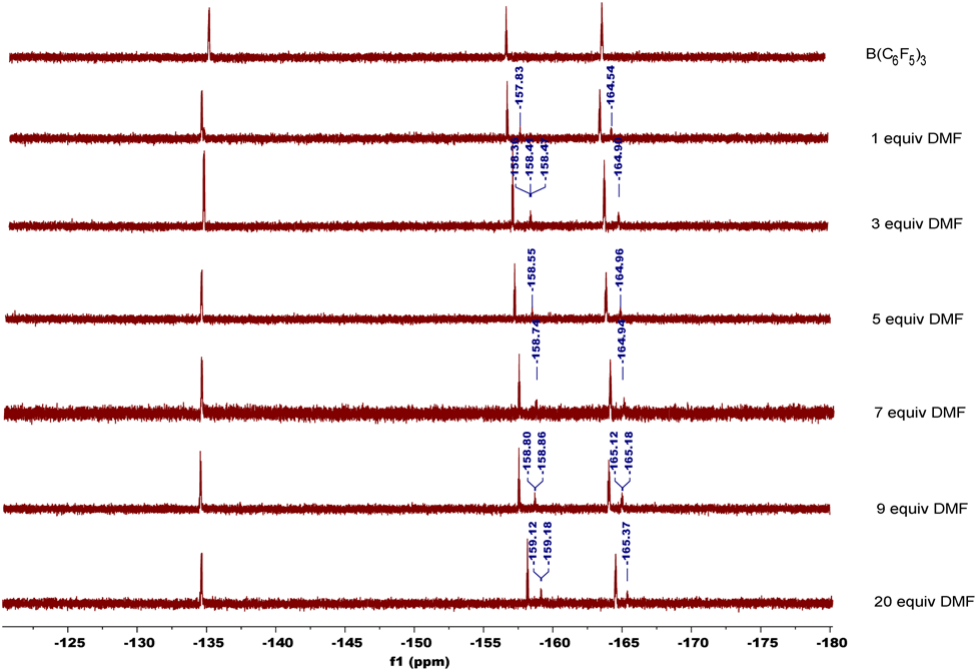
^19^F NMR titration experiments.

**Scheme 2:**
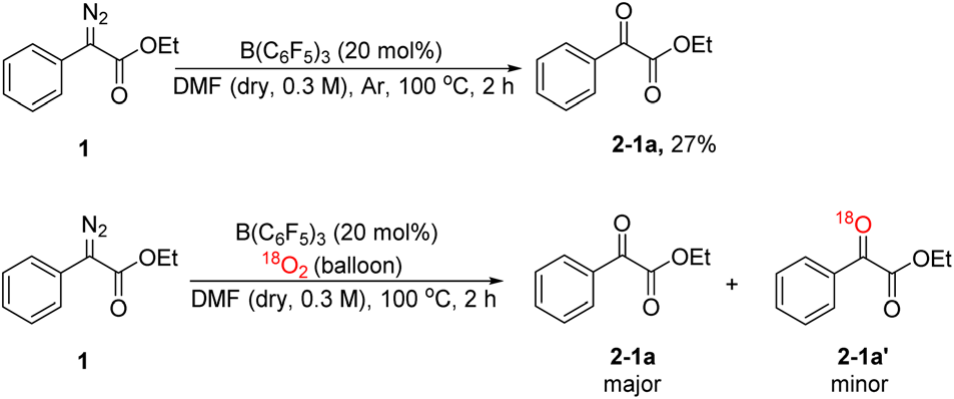
Control experiments.

In order to explore the mechanism, we conducted some control experiments. First, we carried out ^19^F NMR titration experiments using B(C_6_F_5_)_3_ and DMF; when DMF was added to the CDCl_3_ solution of B(C_6_F_5_)_3_, the ^19^F NMR spectra of B(C_6_F_5_)_3_ showed two new peaks at chemical shifts of −158 and −165 ppm (Fig. 2), which were caused by B(C_6_F_5_)_3_ coordinating with DMF. Then, the oxidation reaction was carried out in a pure argon atmosphere, and the yield of 2-1a reduced remarkably, while the results of the control experiment with ^18^O_2_ confirmed that the oxygen source of the product was mainly DMF, and a small portion of it came from ^18^O_2_ (Scheme 2).

**Scheme 3:**
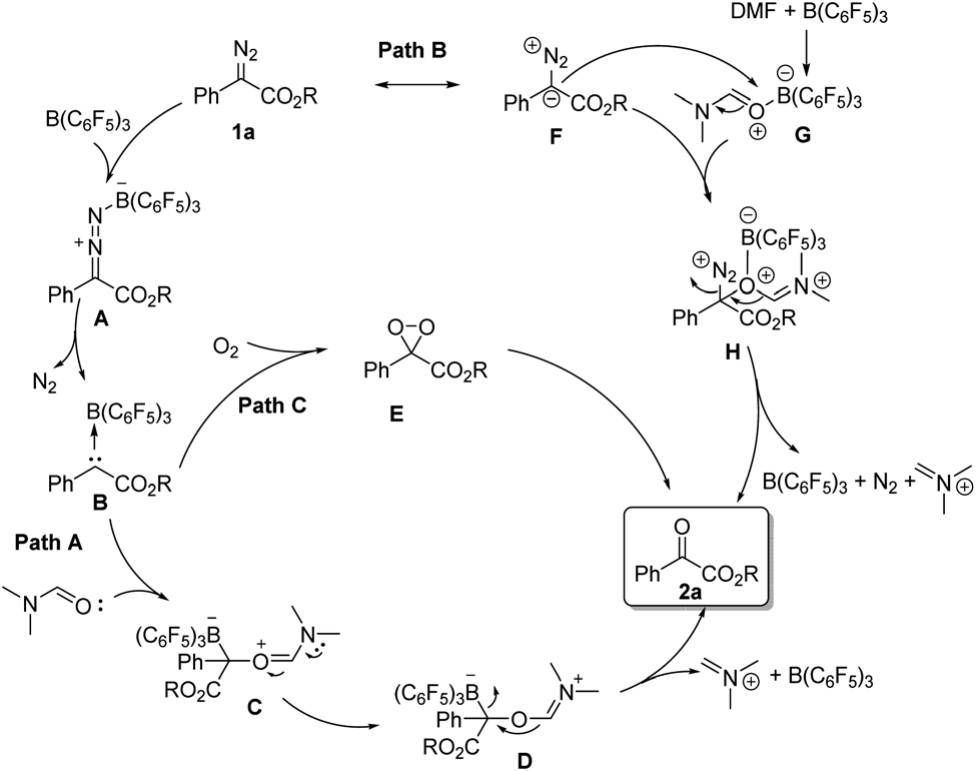
Proposed reaction mechanism.

According to the related literature reports^16*b*,20–22^ and the results of control experiments, we can shed light on the reaction mechanism (Scheme 3). On the one hand, the diazoester compound might be activated by B(C_6_F_5_)_3_ and then release N_2_ to generate carbine intermediate B. Then, the carbene intermediate may directly react with DMF or molecular oxygen to form the corresponding oxidation product 2-1a (path A and path C). On the other hand, B(C_6_F_5_)_3_ might coordinate with DMF, and its coordination intermediate G will then react with the resonance structure F of 1a to form intermediate H. Next, intermediate H will further react to generate the product 2-1a (path B).

## Conclusions

In summary, a metal-free B(C_6_F_5_)_3_-catalyzed oxidative reaction of α-diazoesters in dry DMF under an oxygen atmosphere was reported. This reaction features a broad substrate scope, good compatibility of functional groups and a green environment-friendly nature. Importantly, the control experiments confirmed that the oxygen sources of the product 2-1a were DMF and molecular oxygen. The protocol offers new insight on reactions catalyzed by B(C_6_F_5_)_3_.

## Conflicts of interest

There are no conflicts to declare.

## Supplementary Material

RA-012-D2RA05739E-s001
