# Supplementary material for: B(C6F5)3-catalyzed oxidation of α-diazoesters using DMF and molecular oxygen as oxygen sources
Source: RSC Adv. 2022 Nov 23;12(52):33584–8. doi: 10.1039/d2ra05739e (PMC9682326; doi:10.1039/d2ra05739e)

Electronic Supplementary Material (ESI) for RSC Advances.
This journal is © The Royal Society of Chemistry 2022

Supporting Information

$B(C_6F_5)_3$-Catalyzed Oxidation of α-Diazoesters with DMF and Molecular Oxygen as Oxygen Sources

Bei Wang,‡[a,b,c],Guo-Min Zhang, ‡[a,b,c], Hua Zhang[a,b,c], Ji-Yu Wang*[a,b]

[a]Department of Chemistry, Xihua University, Chengdu 610041, P. R. China.

[b]Chengdu Institute of Organic Chemistry, Chinese Academy of Sciences, Chengdu 610041, P. R. China.

[c]University of Chinese Academy of Sciences, Beijing 100049, P. R. China.

*Corresponding author: Ji-Yu Wang

Email: Jiyuwang@cioc.ac.cn.

Contents

1 General Information 2
2 Experimental sections 2
3 Data of products 3
4 References 8
5 ^{1}H NMR and ^{13}C NMR spectra 9
6 HRMS of $^{18}O_2$ control experiment 36

1 General Information

All reagents and solvents were purchased from Adamas Reagent, Energy Chemical, Alfa Aesa Chemical Co., Aladdin Industrial Corp., Macklin Biochemical Co., Acors Organics, Bide Pharmatech Ltd., and so forth. All chemicals are without purification unless otherwise stated. All heating was done in an oil bath. Thin-layer chromatography (TLC) for analysis was performed on Schleicher&Schuell F1400/LS 254 silica gel plates observed under UV light (λ = 254 nm). ^{1}H-NMR spectra were recorded at 300 MHz or 400 MHz, and carbon NMR (^{13}C {^{1}H}) were recorded at 101 MHz. $CDCl_3$ was used as a solvent as an internal standard (0.00 ppm) unless otherwise stated. High-resolution mass spectra were recorded using a Q-TOF time-of-flight mass spectrometer. Coupling constants (J) were reported in Hertz (Hz).

2 Experimental sections

2.1 The synthesis of α-Diazoesters.

(1) Substrates **1a-1r**, **1t** could be synthesized by the procedure: ethyl arylacetate (3 mmol), TsN_3 (1.5 eq), DBU (1.5 eq) in MeCN (0.2 M) under the Ar atmosphere at 0 ^{o}C stired overlight.[1a] (2) Substrates **1s**, **1u-1x** could be synthesized by the procedure: Aryl iodide (1 mmol), ethyl 2-diazo-3-oxobutanoate (3 eq), $Pd(PPh_3)_4$ (2.5 mol%), NaOH (3 eq) in EtOH (0.3 M) under the Ar atmosphere at room temperature for 6 -10 h.[1b] (3) Substrates **1y** and **1z** could be synthesized by the Curtius or Bamford-Stevens reaction.[1c,d]

2.2 General Process of the oxidative reaction of α-Diazoesters.

The catalyst $B(C_6F_5)_3$ (30 mg, 20 mol%) was put into a Schlenk tube equipped with a rubber balloon filled with pure O_2. The tube was evacuated and then backfilled with oxygen three times. Subsequently, α-Diazoesters **1** (0.3 mmol) in dry DMF (1 mL) was added to the tube. The reaction mixture was heated to 100 °C in an oil bath and stirred until completion, as indicated by TLC. After cooling to room temperature, ethyl acetate (5 mL) and water (5 mL) were added to the reaction mixture. The aqueous layer was extracted by ethyl acetate (3 × 5 mL). The combined organic phases were washed with brine and dried over anhydrous sodium sulfate. The solvent was removed by rotary evaporation and purified by silica gel column chromatography with petroether/ethyl acetate (50:1) as the eluent to give the desired products.

3 Data of products

*Ethyl 2-oxo-2-phenylacetate (**2-1a**).*[2a]

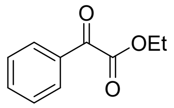

Yield: 46 mg, 86% yield; as a colorless liquid; Rf: 0.5 (petroleum ether/EtOAc = 10/1). ^{1}H NMR (400 MHz, $CDCl_3$) δ 8.15 (dd, *J* = 8.1, 2.5 Hz, 2H), 7.80 (t, *J* = 7.5 Hz, 1H), 7.65 (t, *J* = 7.7 Hz, 2H), 4.64-4.54 (m, 2H), 1.56 (td, *J* = 7.5, 2.0 Hz, 3H). ^{13}C {^{1}H} NMR (101 MHz, $CDCl_3$) δ 186.4, 163.8, 134.9, 132.4, 130.1, 130.0, 128.9, 62.3, 14.1.

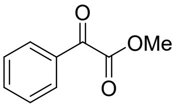

*Methyl 2-oxo-2-phenylacetate (**2-1b**).*[2a]

Yield: 39 mg, 80% yield; as a colorless liquid; Rf: 0.5 (petroleum ether/EtOAc = 10/1). ^{1}H NMR (400 MHz, $CDCl_3$) δ 8.05-7.99 (m, 2H), 7.67 (ddq, *J* = 8.6, 7.2, 1.1 Hz, 1H), 7.52 (dd, *J* = 8.4, 7.2 Hz, 2H), 3.98 (d, *J* = 0.8 Hz, 3H). ^{13}C {^{1}H} NMR (101 MHz, $CDCl_3$) δ 186.0, 164.0, 135.0, 132.4, 130.1, 128.9, 52.8, 52.8.

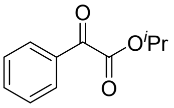

*Isopropyl 2-oxo-2-phenylacetate (**2-1c**).*[2a]

Yield: 38 mg, 66% yield; as a colorless liquid; Rf: 0.5 (petroleum ether/EtOAc = 10/1). ^{1}H NMR (400 MHz, $CDCl_3$) δ 7.99 (dd, *J* = 8.2, 1.4 Hz, 2H), 7.70-7.61 (m, 1H), 7.51 (t, *J* = 7.8 Hz, 2H), 5.33 (m, *J* = 6.3 Hz, 1H), 1.41 (d, *J* = 6.2 Hz, 6H). ^{13}C {^{1}H} NMR (101 MHz, $CDCl_3$) δ 186.7, 163.6, 134.8, 132.5, 130.0, 128.9, 70.7, 70.7, 21.7, 21.7.

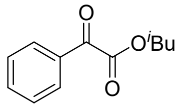

*Isobutyl 2-oxo-2-phenylacetate (**2-1d**).*[2a]

Yield: 44 mg, 72% yield; as a colorless liquid; Rf: 0.5 (petroleum ether/EtOAc = 10/1). ^{1}H NMR (300 MHz, $CDCl_3$) δ 8.05-7.96 (m, 1H), 7.66 (t, *J* = 7.4 Hz, 2H), 7.57-7.46 (m, 2H), 4.18 (d, *J* = 6.7 Hz, 2H), 2.09 (dt, *J* = 13.3, 6.7 Hz, 1H), 1.01 (d, *J* = 6.8 Hz, 6H). ^{13}C {^{1}H} NMR (101 MHz, $CDCl_3$) δ 186.5, 164.1, 134.9, 132.5, 130.0, 129.2, 128.9, 128.5, 72.1, 27.7, 19.0.

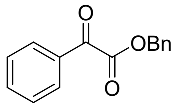

*Benzyl 2-oxo-2-phenylacetate (**2-1e**).*[2a]

Yield: 59 mg, 82% yield; as a colorless liquid; Rf: 0.5 (petroleum ether/EtOAc = 10/1). ^{1}H NMR (400 MHz, $CDCl_3$) δ 7.97 (dd, J = 8.4, 1.4 Hz, 1H), 7.64 (t, J = 7.5 Hz, 0H), 7.53-7.41 (m, 1H), 7.45-7.33 (m, 1H), 5.42 (s, 2H). ^{13}C {^{1}H} NMR (101 MHz, $CDCl_3$) δ 186.1, 163.7, 135.0, 134.5, 132.4, 130.0, 128.9, 128.8, 128.8, 128.6, 67.8.

*Allyl 2-oxo-2-phenylacetate (**2-1f**).*[2a]

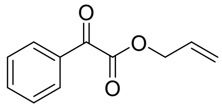

Yield: 47 mg, 83% yield; as a colorless liquid; Rf: 0.4 (petroleum ether/EtOAc = 10/1). ^{1}H NMR (400 MHz, $CDCl_3$) δ 8.04-7.98 (m, 2H), 7.70-7.62 (m, 1H), 7.52 (t, *J* = 7.8

Hz, 2H), 6.02 (ddt, J = 16.6, 10.4, 5.9 Hz, 1H), 5.45 (dq, J = 17.2, 1.4 Hz, 1H), 5.35 (dq, J = 10.4, 1.1 Hz, 1H), 4.88 (dt, J = 5.9, 1.3 Hz, 2H). ^{13}C {^{1}H} NMR (101 MHz, $CDCl_3$) δ 186.1, 163.4, 135.0, 132.4, 130.8, 130.1, 128.9, 120.1, 66.6.

*Prop-2-yn-1-yl 2-oxo-2-phenylacetate (**2-1g**).*[2a]

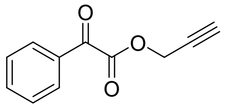

Yield: 40 mg, 71% yield; as a colorless liquid; Rf: 0.5 (petroleum ether/EtOAc = 10/1). ^{1}H NMR (300 MHz, $CDCl_3$) δ 8.08-7.98 (m, 2H), 7.68 (t, J = 7.5 Hz, 1H), 7.53 (t, J = 7.6 Hz, 2H), 4.97 (d, J = 2.5 Hz, 2H), 2.60 (t, J = 2.5 Hz, 1H). ^{13}C {^{1}H} NMR (101 MHz, $CDCl_3$) δ 185.2, 162.8, 135.2, 132.3, 130.1, 129.0, 76.3, 53.3.

*2-hydroxyethyl 2-oxo-2-phenylacetate (**2-1h**).*[2b]

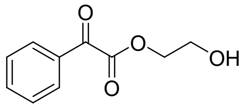

Yield: 29 mg, 51% yield; as a colorless liquid; Rf: 0.5 (petroleum ether/EtOAc =1/1). ^{1}H NMR (400 MHz, $CDCl_3$) δ 8.03 (dd, J = 8.4, 1.3 Hz, 2H), 7.71-7.60 (m, 1H), 7.56-7.46 (m, 2H), 4.54-4.47 (m, 2H), 3.99-3.92 (m, 2H), 2.58 (s, 1H). ^{13}C {^{1}H} NMR (101 MHz, $CDCl_3$) δ 186.1, 163.6, 135.1, 132.3, 130.2, 128.9, 67.4, 60.6.

*Ethyl 2-(2-methyl)-2-oxoacetate (**2-1i**).*[1c]

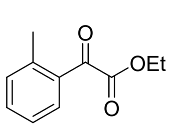

Yield: 25 mg, 44% yield; as a colorless liquid; Rf: 0.5 (petroleum ether/EtOAc = 10/1). δ 7.69 (dd, J = 7.7, 1.5 Hz, 1H), 7.58-7.44 (m, 1H), 7.39-7.26 (m, 2H), 4.43 (q, J = 7.2 Hz, 2H), 2.60 (s, 3H), 1.41 (t, J = 7.1 Hz, 3H). ^{13}C {^{1}H} NMR (101 MHz, $CDCl_3$) δ186.1, 164.0, 146.2, 130.6, 130.1, 130.0, 129.6, 62.2, 21.9, 14.1.

*Ethyl 2-(2-chlorophenyl)-2-oxoacetate (**2-1j**).*[2c]

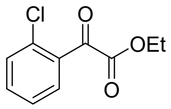

Yield: 32 mg, 51% yield; as a colorless liquid; Rf: 0.5 (petroleum ether/EtOAc = 10/1). ^{1}H NMR (300 MHz, $CDCl_3$) δ 7.76 (dd, J = 7.7, 1.7 Hz, 1H), 7.56 – 7.49 (m, 1H), 7.47 – 7.36 (m, 2H), 4.42 (q, J = 7.1 Hz, 3H), 1.40 (t, J = 7.1 Hz, 3H). ^{13}C {^{1}H} NMR (101 MHz, $CDCl_3$) δ 186.8, 163.3, 134.5, 134.1, 133.6, 131.8, 130.7, 127.4, 63.0, 14.1.

*Ethyl 2-(3-chlorophenyl)-2-oxoacetate (**2-1k**).*[2c]

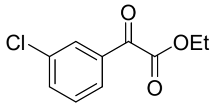

Yield: 46 mg, 72% yield; as a colorless liquid; Rf: 0.5 (petroleum ether/EtOAc = 10/1). ^{1}H NMR (300 MHz, $CDCl_3$) δ 8.01 (d, J = 1.9 Hz, 1H), 7.96-7.86 (m, 1H), 7.62 (ddd, J = 8.0, 2.1, 1.1 Hz, 1H), 7.46 (t, J = 7.9 Hz, 1H), 4.45 (q, J = 7.2 Hz, 2H), 1.42 (t, J = 7.1 Hz, 3H). ^{13}C {^{1}H} NMR (101 MHz, $CDCl_3$) δ 184.8, 163.0, 135.2, 134.8, 134.0, 130.2, 129.8, 128.2, 62.6, 14.1.

*Ethyl 2-oxo-2-(3-(trifluoromethyl)phenyl)acetate (**2-1l**).*[2c]

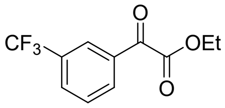

Yield: 55 mg, 75% yield; as a colorless liquid; Rf: 0.5 (petroleum ether/EtOAc = 10/1). ^{1}H NMR (300 MHz, $CDCl_3$) δ 8.32 (s, 1H), 8.28-8.19 (d, 1H), 7.91 (d, J = 7.8 Hz, 1H), 7.67 (t, J = 7.8 Hz, 1H), 4.48 (q, J = 7.2 Hz, 2H), 1.44 (t, J = 7.1 Hz, 3H). ^{13}C {^{1}H} NMR (101 MHz, CDCl3) δ 184.6, 162.7, 133.2, 133.2, 133.2, 131.2, 131.1, 131.1, 131.1, 129.6, 126.9, 126.9, 126.9, 126.8, 122.0, 62.8, 14.0. ^{19}F NMR (376 MHz, $CDCl_3$) δ -63.0

*Ethyl 2-(4-methyl)-2-oxoacetate (**2-1m**).*[1c]

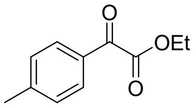

Yield: 35 mg, 62% yield; as a colorless liquid; Rf: 0.5 (petroleum ether/EtOAc = 10/1). ^{1}H NMR (400 MHz, $CDCl_3$) δ 7.90 (d, J = 8.3 Hz, 2H), 7.30 (d, J = 7.6 Hz, 2H), 4.43 (q, J = 7.2 Hz, 2H), 2.43 (s, 3H), 1.41 (t, J = 7.2 Hz, 3H). ^{13}C {^{1}H} NMR (101 MHz, $CDCl_3$) δ 184.9, 163.2, 141.6, 131.4, 130.9, 129.3, 62.5, 21.9, 14.1.

*Ethyl 2-(4-methoxyl)-2-oxoacetate (**2-1n**).* [2c]

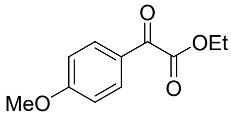

Yield: 31 mg, 50% yield; as a colorless liquid; Rf: 0.5 (petroleum ether/EtOAc = 10/1). ^{1}H NMR (300 MHz, $CDCl_3$) δ 8.05-7.96 (m, 1H), 7.02-6.93 (m, 1H), 4.43 (q, J = 7.1 Hz, 2H), 3.89 (s, 3H), 1.47-1.36 (m, 3H). ^{13}C {^{1}H} NMR (101 MHz, $CDCl_3$) δ 184.9, 165.0, 164.1, 156.5, 143.4, 132.6, 125.5, 121.7, 114.6, 114.2, 63.6, 62.1, 55.6, 14.1.

Ethyl 2-(4-fluorophenyl)-2-oxoacetate (***2-1o***).[2c]

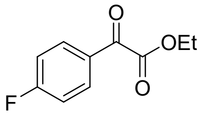

Yield: 47 mg, 80% yield; as a colorless liquid; Rf: 0.5 (petroleum ether/EtOAc = 10/1). ^{1}H NMR (300 MHz, $CDCl_3$) δ 8.03-7.93 (m, 2H), 7.54-7.44 (m, 2H), 4.45 (q, J = 7.1 Hz, 2H), 1.42 (t, J = 7.1 Hz, 3H). ^{13}C {^{1}H} NMR (101 MHz, $CDCl_3$) δ 184.5, 168.1, 165.5, 163.4, 133.0, 132.9, 128.5, 116.3, 116.1, 62.5, 14.1. ^{19}F NMR (376 MHz, $CDCl_3$) δ -101.3.

Ethyl 2-(4-chlorophenyl)-2-oxoacetate (***2-1p***).[2c]

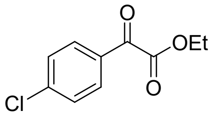

Yield: 49 mg, 77% yield; as a colorless liquid; Rf: 0.5 (petroleum ether/EtOAc = 10/1). ^{1}H NMR (300 MHz, $CDCl_3$) δ 8.03-7.93 (m, 2H), 7.54-7.44 (m, 2H), 4.45 (q, J = 7.1 Hz, 2H), 1.42 (t, J = 7.1 Hz, 3H). ^{13}C {^{1}H} NMR (101 MHz, $CDCl_3$) δ 184.9, 163.2, 141.6, 131.4, 130.9, 129.3, 62.5, 14.1.

Ethyl 2-(4-bromophenyl)-2-oxoacetate (***2-1q***).[2c]

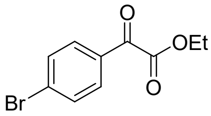

Yield: 57 mg, 74% yield; as a colorless liquid; Rf: 0.5 (petroleum ether/EtOAc = 10/1). ^{1}H NMR (300 MHz, $CDCl_3$) δ 7.90 (d, J = 8.5 Hz, 2H), 7.66 (d, J = 8.5 Hz, 2H), 4.44 (q, J = 7.2 Hz, 2H), 1.47-1.37 (t, 3H). ^{13}C {^{1}H} NMR (101 MHz, $CDCl_3$) δ 185.1, 163.1, 132.3, 131.4, 131.3, 130.5, 62.6, 14.1.

Ethyl 2-(2, 3-dichlorophenyl)-2-oxoacetate (***2-1r***).[2c]

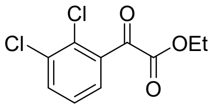

Yield: 36 mg, 48% yield; as a colorless liquid; Rf: 0.4 (petroleum ether/EtOAc = 10/1). ^{1}H NMR (300 MHz, $CDCl_3$) δ 7.68 (dd, J = 8.0, 1.6 Hz, 1H), 7.60 (dd, *J* = 7.8, 1.6 Hz, 1H), 4.42 (q, *J* = 7.2 Hz, 2H), 1.40 (t, *J* = 7.2 Hz, 3H). ^{13}C {^{1}H} NMR (101 MHz, $CDCl_3$) δ 185.9, 162.3, 135.9, 134.6, 134.2, 131.7, 129.4, 127.9, 63.1, 13.9.

Ethyl 2-oxo-2-(4-(phenylethynyl)phenyl)acetate (***2-1s***).

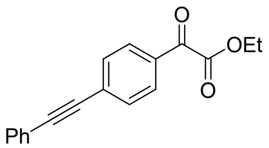

Yield: 54 mg, 65% yield; white solid; m.p.112.1-113.0 °C; Rf: 0.4 (petroleum ether/EtOAc = 7/1). ^{1}H NMR (300 MHz, $CDCl_3$) δ 8.06-7.97 (m, 2H), 7.68-7.61 (m, 2H), 7.60-7.51 (m, 2H), 7.46-7.33 (m, 3H), 4.46 (q, *J* = 7.1 Hz, 2H), 1.44 (t, *J* = 7.1 Hz, 3H). ^{13}C {^{1}H} NMR (101 MHz, $CDCl_3$) δ 185.4, 163.5, 131.9, 131.8, 131.5, 130.1, 130.0, 129.1, 128.5, 122.4, 94.1, 88.4, 62.5, 14.1.

HRMS (ESI): m/z: $[M+Na]^+$ Calcd. for $C_{18}H_{14}O_3Na^+$: 301.0841; Found: 301.0839.

Ethyl 2-(naphthalen-2-yl)-2-oxoacetate (***2-1t***).[2c]

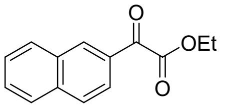

Yield: 53 mg, 77% yield; white solid; m.p.112.1-113.0 °C; Rf: 0.4 (petroleum ether/EtOAc = 7/1). ^{1}H NMR (300 MHz, $CDCl_3$) δ 8.56 (s, 1H), 8.05 (dd, *J* = 8.7, 1.7 Hz, 1H), 7.98 (d, *J* = 8.2 Hz, 1H), 7.93 (d, *J* = 8.7 Hz, 1H), 7.90 (d, *J* = 7.5 Hz, 1H), 7.66 (ddd, *J* = 8.3, 6.9, 1.4 Hz, 1H), 7.58 (ddd, *J* = 8.2, 6.9, 1.3 Hz, 1H), 4.51 (q, *J* = 7.1 Hz, 2H), 1.46 (t, *J* = 7.2 Hz, 3H). ^{13}C {^{1}H} NMR (101 MHz, $CDCl_3$) 186.3, 163.9, 136.4, 133.5, 132.3, 130.0, 129.8, 129.6, 128.9, 127.9, 127.2, 124.0, 62.4, 14.2.

Ethyl 2-(9,10-dioxo-8a,9,10,10a-tetrahydroanthracen-2-yl)-2-oxoacetate (***2-1u***).

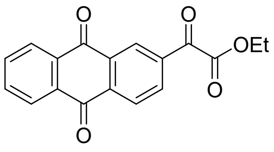

Yield: 73 mg, 79% yield; yellow solid; m.p.222.1-223.0 °C; Rf: 0.4 (petroleum ether/EtOAc = 7/1). ^{1}H NMR (300 MHz, $CDCl_3$) δ 8.90 (s, 1H), 8.43 (s, 2H), 8.33 (td, *J* = 6.2, 3.3 Hz, 2H), 7.90-7.81 (m, 2H), 4.52 (q, *J* = 7.1 Hz, 2H), 1.47 (t, *J* = 7.2 Hz, 3H). ^{13}C {^{1}H} NMR (101 MHz, $CDCl_3$) 184.7, 182.3, 181.9, 162.5, 136.9, 136.7, 134.7, 134.6, 134.3, 133.7, 133.3, 133.3, 129.4, 127.9, 127.5, 127.5, 63.0, 14.1.

HRMS (ESI): m/z: $[M+H]^+$ Calcd. for $C_{18}H_{13}O_5^+$: 309.0763; Found: 309.0760.

Ethyl 2-(benzo[d][1,3]dioxol-5-yl)-2-oxoacetate (***2-1v***).[2d]

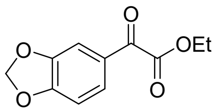

Yield: 27 mg, 40% yield; as a colorless liquid; Rf: 0.4 (petroleum ether/EtOAc = 7/1). ^{1}H NMR (400 MHz, $CDCl_3$) δ 7.60 (dd, *J* = 8.2, 1.7 Hz, 1H), 7.47 (d, *J* = 1.7 Hz, 1H), 6.88 (d, *J* = 8.2 Hz, 1H), 6.08 (s, 2H), 4.42 (q, *J* = 7.2 Hz, 2H), 1.40 (t, *J* = 7.1 Hz,

3H). ^{13}C {^{1}H} NMR (101 MHz, $CDCl_3$) δ 184.6, 164.0, 153.5, 148.5, 127.8, 127.2, 108.7, 108.3, 103.0, 102.2, 62.3, 14.1.

HRMS (ESI): m/z: $[M+Na]^+$ Calcd. for $C_{11}H_{10}O_5Na^+$: 245.0426; Found: 205.0426.

Ethyl 2-oxo-2-(quinolin-3-yl)acetate (***2-1w***).[2e]

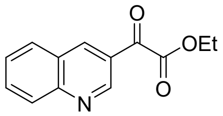

Yield: 51 mg, 74% yield; as a colorless liquid; Rf: 0.4 (petroleum ether/EtOAc = 7/1). ^{1}H NMR (300 MHz, $CDCl_3$) δ 9.46 (d, J = 2.2 Hz, 1H), 8.92 (d, J = 2.2 Hz, 1H), 8.17 (d, J = 8.5 Hz, 1H), 8.00-7.83 (m, 2H), 7.71-7.60 (m, 1H), 4.57-4.44 (m, 1H), 1.46 (t, J = 7.2 Hz, 2H). ^{13}C {^{1}H} NMR (101 MHz, $CDCl_3$) 184.4, 162.5, 150.3, 149.5, 140.4, 133.1, 129.8, 129.6, 127.9, 126.5, 125.3, 77.3, 77.0, 76.7, 62.8, 14.1.

HRMS (ESI): m/z: $[M+H]^+$ Calcd. for $C_{13}H_{12}NO_3^+$: 230.0817; Found: 230.0819.

Ethyl 2-oxo-2-(4-(((2,5,7,8-tetramethyl-2-(4,8,12-trimethyltridecyl)chroman-6-yl)oxy)methyl)phenyl) acetate (***2-1x***).[2i]

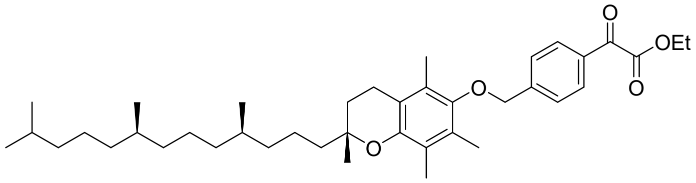

Yield: 80 mg, 43% yield; as a colorless liquid; Rf: 0.4 (petroleum ether/EtOAc = 7/1). ^{1}H NMR (300 MHz, $CDCl_3$) δ 8.05 (d, J = 8.3 Hz, 2H), 7.64 (d, J = 8.1 Hz, 2H), 4.79 (s, 2H), 4.47 (q, J = 7.1 Hz, 2H), 2.59 (t, J = 6.8 Hz, 2H), 2.22-2.08 (m, 9H), 1.80 (h, J = 6.6 Hz, 2H), 1.58 (s, 3H), 1.44 (t, J = 7.1 Hz, 8H), 1.25 (d, J = 3.3 Hz, 10H), 1.13 (dd, J = 16.5, 9.5 Hz, 6H), 0.86 (t, J = 6.4 Hz, 12H). ^{13}C {^{1}H} NMR (101 MHz, $CDCl_3$) δ 186.0, 163.8, 148.1, 147.8, 145.7, 131.7, 130.3, 127.7, 127.3, 125.8, 123.1, 117.7, 74.9, 73.6, 62.3, 40.0, 40.0, 39.4, 37.6, 37.5, 37.5, 37.4, 37.4, 37.3, 32.8, 32.8, 32.7, 32.7, 31.3, 31.2, 28.0, 24.8, 24.8, 24.4, 23.9, 22.7, 22.6, 21.0, 20.7, 19.7, 19.7, 19.7, 19.6, 14.1, 12.8, 11.9, 11.8.

Benzophenone (***2-1y***).[2f]

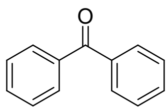

Yield: 36 mg, 66% yield; white solid; m.p.47-49 °C; Rf: 0.5 (petroleum ether/EtOAc = 15/1). ^{1}H NMR (300 MHz, $CDCl_3$) δ 7.81 (d, J = 6.9 Hz, 4H), 7.58 (t, J = 7.4 Hz, 2H), 7.48 (t, J = 7.7 Hz, 4H). ^{13}C {^{1}H} NMR (101 MHz, $CDCl_3$) δ 196.7, 137.6, 132.4, 130.1, 128.3.

Indoline-2,3-dione (***2-1z***).[2g]

Yield: 21 mg, 48% yield; red solid; m.p.203.1-204.2 °C; Rf: 0.4 (petroleum ether/EtOAc = 7/1). ^{1}H NMR (400 MHz, DMSO-d_6) δ 11.01 (s, 1H), 7.58-7.54 (m, 1H), 7.48 (d, J = 7.4 Hz, 1H), 7.04 (t, J = 7.5

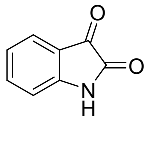

Hz, 1H), 6.89 (d, J = 7.9 Hz, 1H). ^{13}C NMR (101 MHz, DMSO-d_6) δ 184.8, 159.8, 151.2, 138.8, 125.1, 123.2, 118.3, 112.6.

4 References

[1] (a) Z. Guo, H. Huang, Q. Fu, W. Hu, *Synlett.,* 2006, **15**, 2486-2488. (b) F. Ye, C.-P. Wang, Y. Zhang, J.-B. Wang, *Angew. Chem., Int. Ed.,* 2014, **53**, 11625-11628. (c) Y. Yu, Q. Sha, H. Cui, K. S. Chandler, M. P. Doyle, *Org. Lett.,* 2018, **20**, 776-779. (d) H.-H. Wang, Y.-W. Zhao, X.-B. Wan, *J. Org. Chem.,* 2020, **85**, 3050-3058.

[2] (a) T. Shao, X. Fang, J. Zhou, C. Jin, X.-Y. Yang, F.-H. Wu, *Synlett.,* 2017, **28**, 2018-2023. (b) Q. Yu, Y.-T. Zhang, J.-P. Wan, *Green Chem.,* 2019, **21**, 3436-3441. (c) R. Ntaganda, T. Milovic, J. Tiburcio, A. N. Thadani, *Chem. Commun*. 2008, **34**, 4052-4054. (d) J. C. Leung, L. M. Geary, T.-Y. Chen, J. R. Zbieg, M. J. Krische, *J. Am. Chem. Soc*., 2012, **134**, 15700-15703. (e) A. Klapars, E. Vedejs, *Chem. Heterocycl. Com.,* 2004, **40**, 759-766. (f) S. Kamijo, K. Tao, G. Takao, H. Tonoda, T. Murafuji, *Org. Lett.* 2015, **17**, 3326-3329. (h) N. R. O'Connor, P. Bolgar, B. M. Stoltz, *Tetrahedron Lett*. 2016, **57**, 849-851. (i) F. Ye, C. Wang, Y. Zhang, J. Wang, *Angew. Chem. Int. Ed.* 2014, **53**, 11625-11628.

5 ^{1}H NMR and ^{13}C NMR spectra

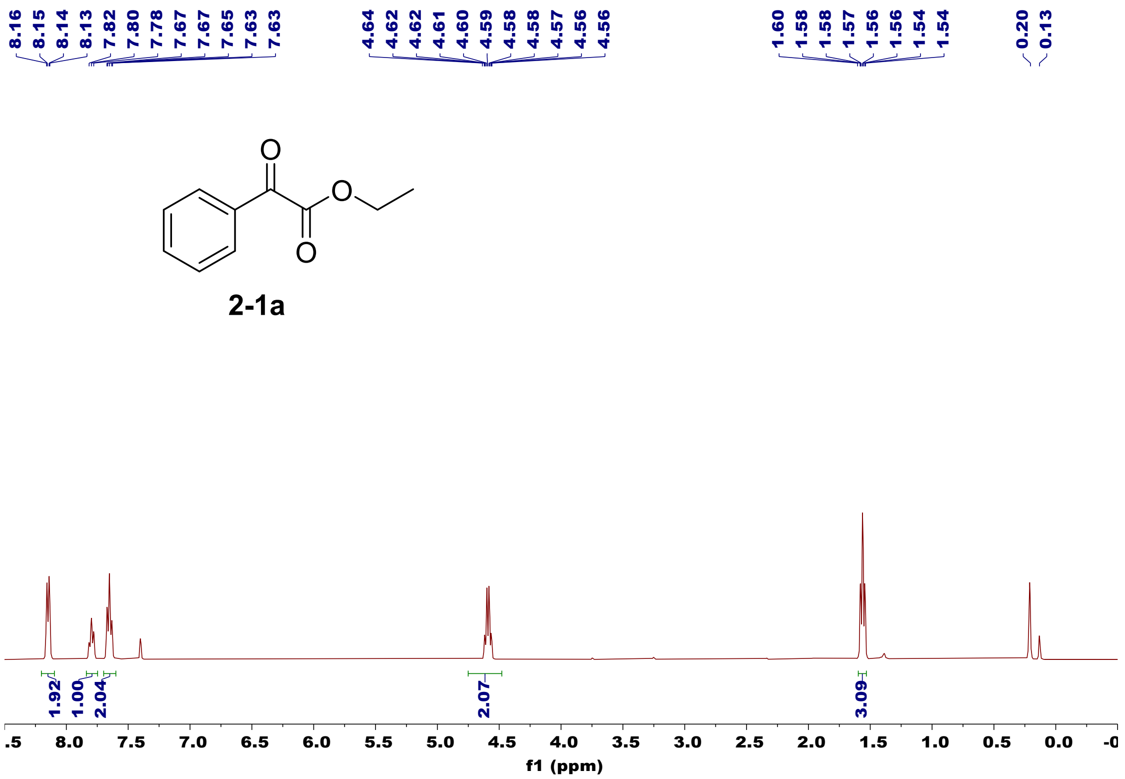

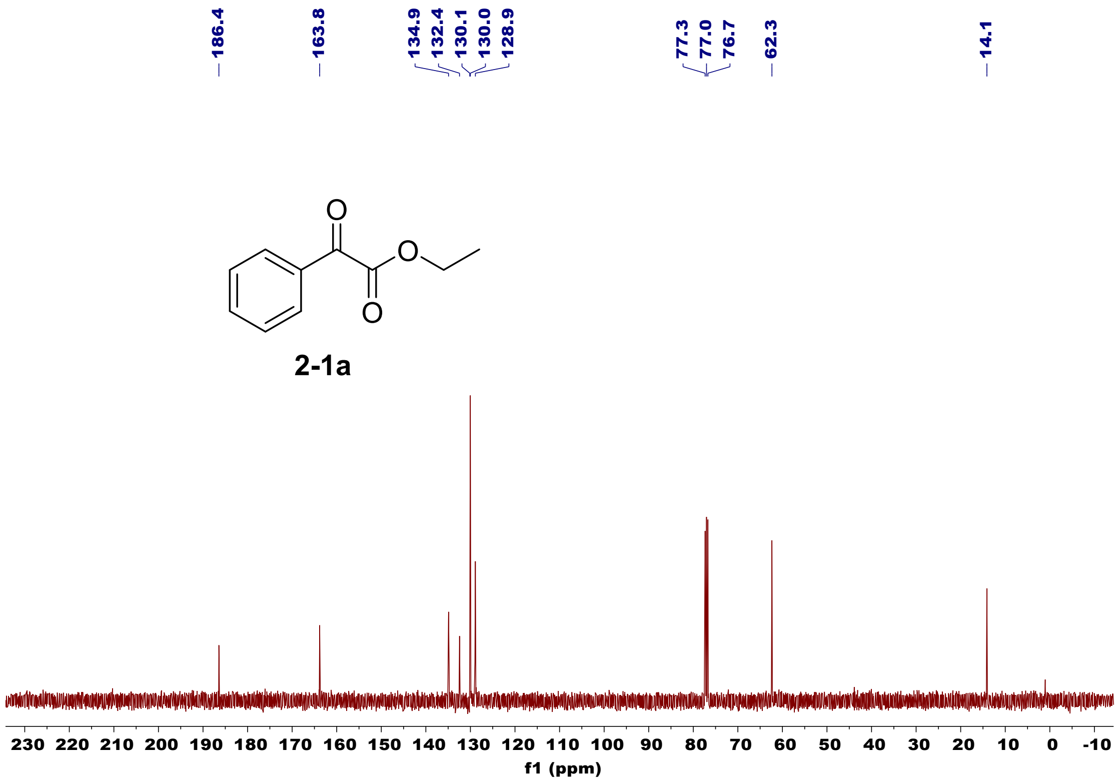

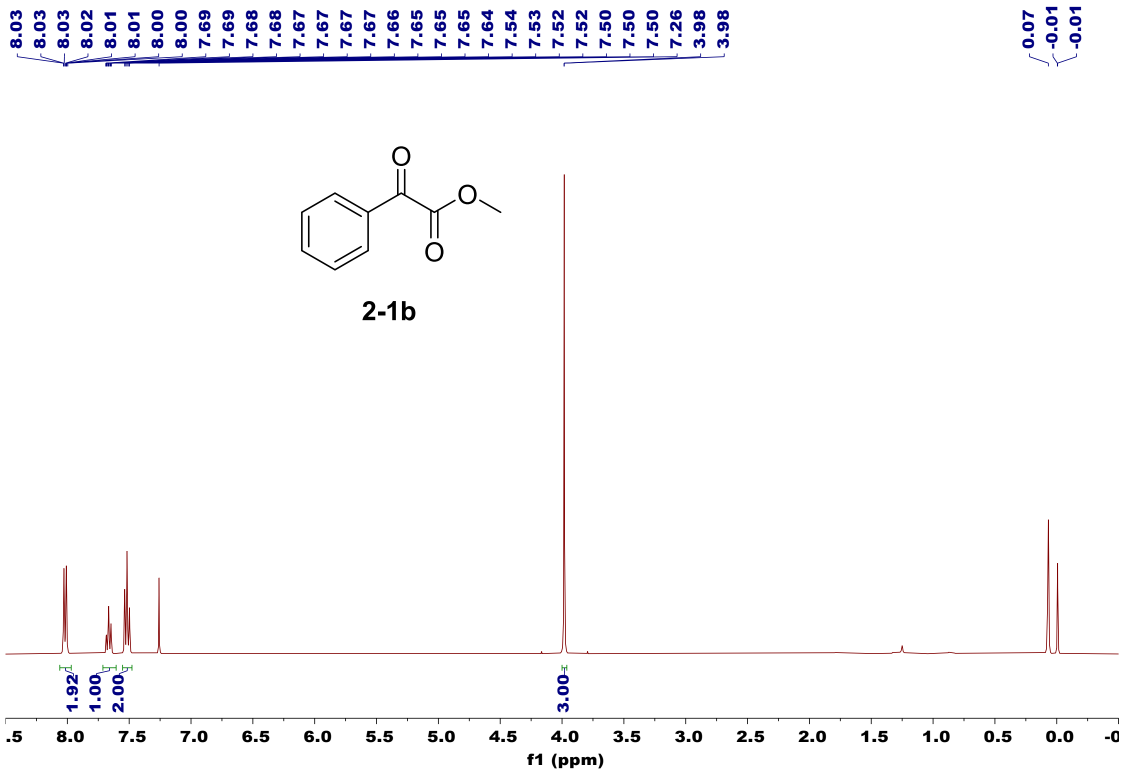
8.03 8.03 8.03 8.02 8.01 8.01 8.00 8.00 7.69 7.69 7.68 7.68 7.67 7.67 7.67 7.67 7.66 7.65 7.65 7.65 7.64 7.54 7.53 7.52 7.52 7.50 7.50 7.50 7.26 3.98 3.98
0.07 -0.01 -0.01
2-1b
1.92 1.00 2.00 3.00
f1 (ppm)

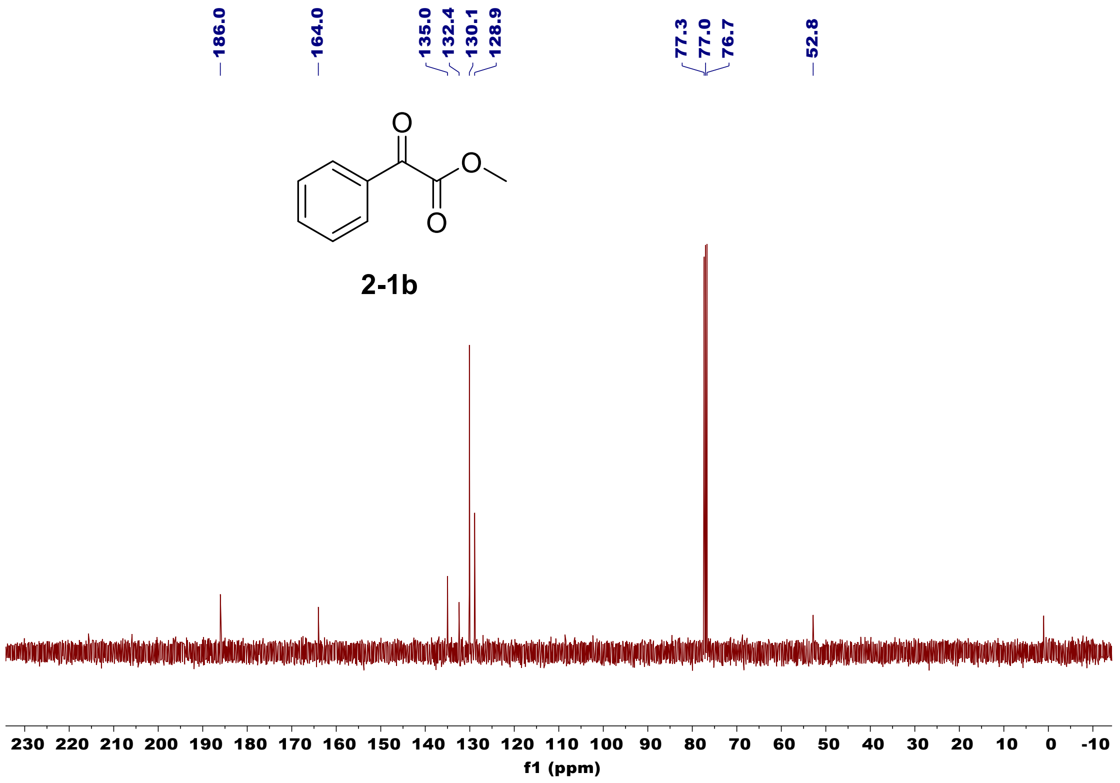
186.0 164.0 135.0 132.4 130.1 128.9 77.3 77.0 76.7 52.8
2-1b
f1 (ppm)

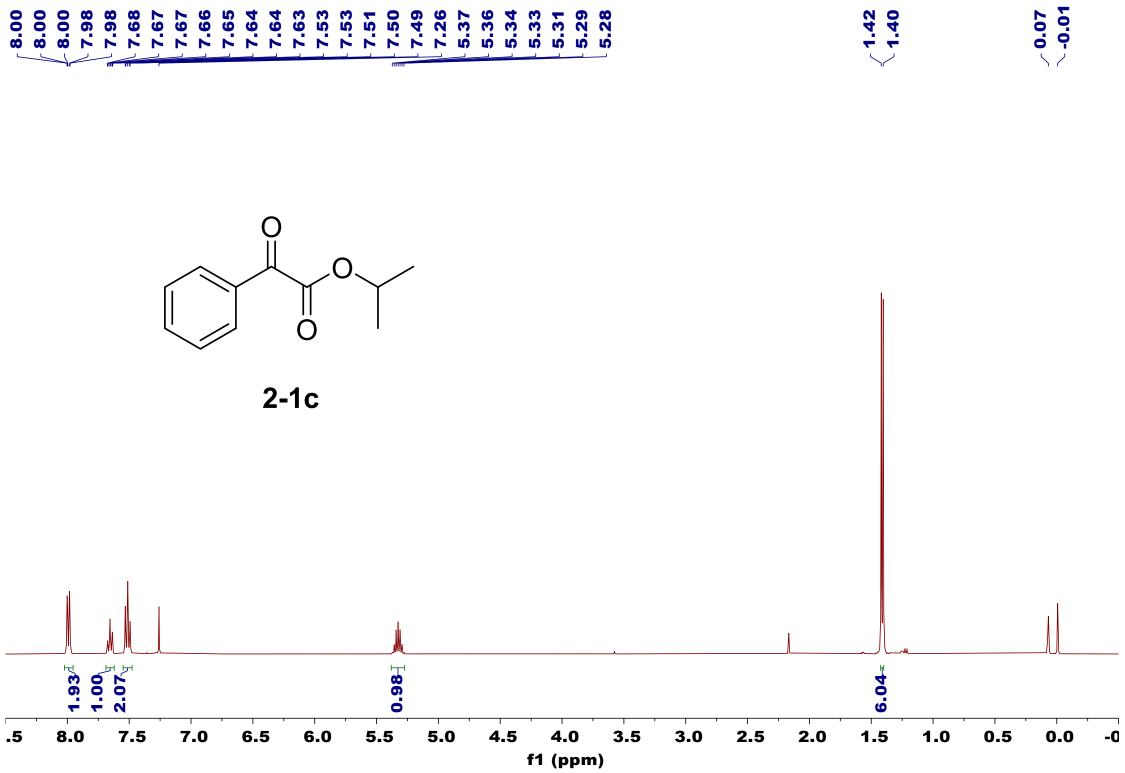
8.00
8.00
8.00
7.98
7.98
7.68
7.67
7.67
7.66
7.65
7.64
7.64
7.63
7.53
7.53
7.51
7.50
7.49
7.26
5.37
5.36
5.34
5.33
5.31
5.29
5.28
1.42
1.40
0.07
-0.01
O
O
O
2-1c
1.93
1.00
2.07
0.98
6.04
f1 (ppm)

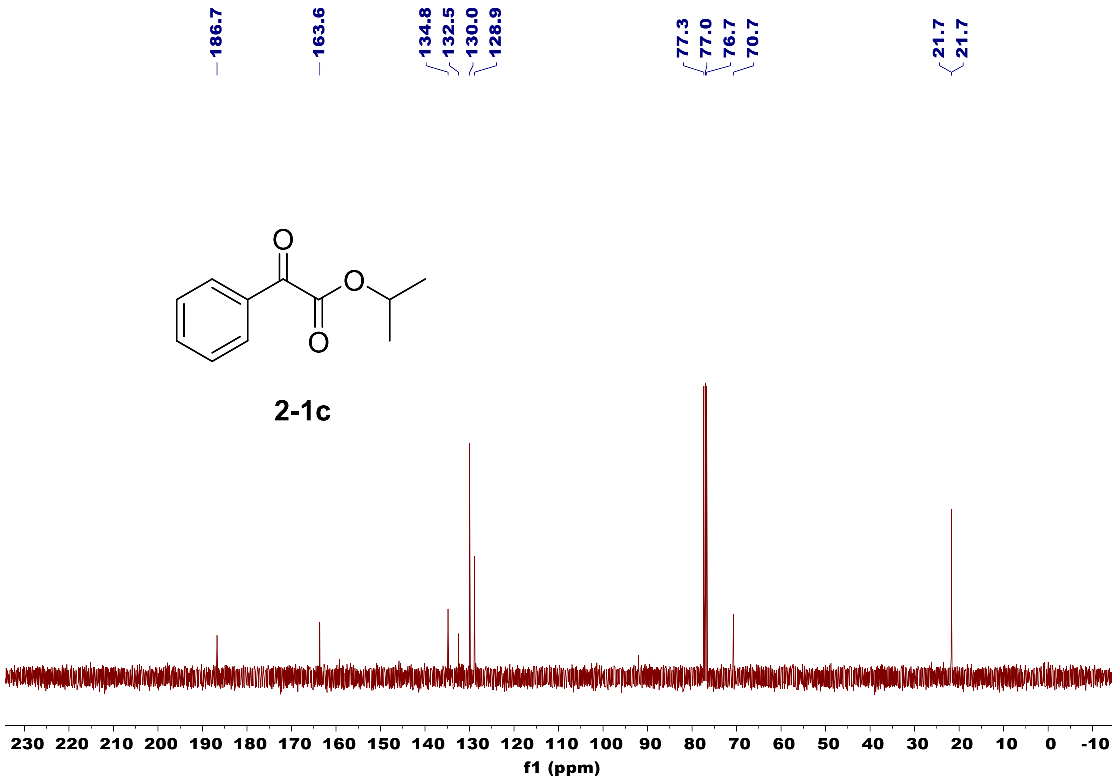
186.7
163.6
134.8
132.5
130.0
128.9
77.3
77.0
76.7
70.7
21.7
21.7
O
O
O
2-1c
f1 (ppm)

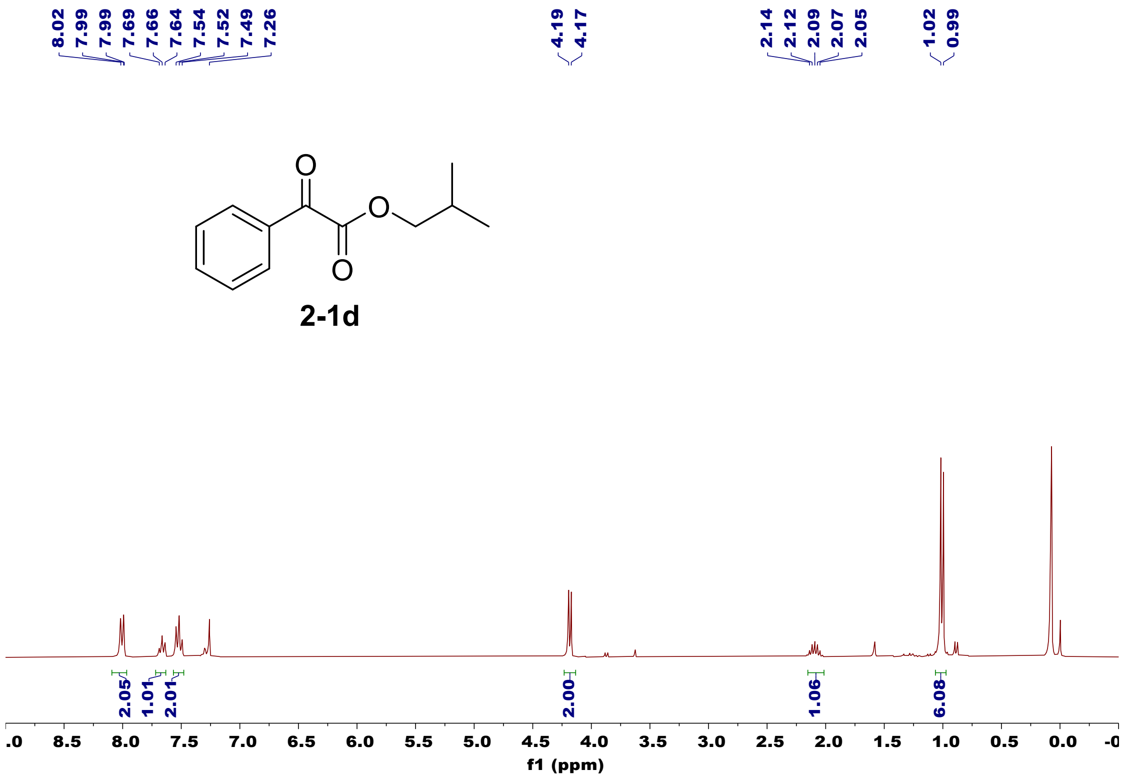
8.02 7.99 7.99 7.69 7.66 7.64 7.54 7.52 7.49 7.26
4.19 4.17
2.14 2.12 2.09 2.07 2.05
1.02 0.99
O
O
O
2-1d
2.05 1.01 2.01
2.00
1.06
6.08
.0 8.5 8.0 7.5 7.0 6.5 6.0 5.5 5.0 4.5 4.0 3.5 3.0 2.5 2.0 1.5 1.0 0.5 0.0 -0
f1 (ppm)

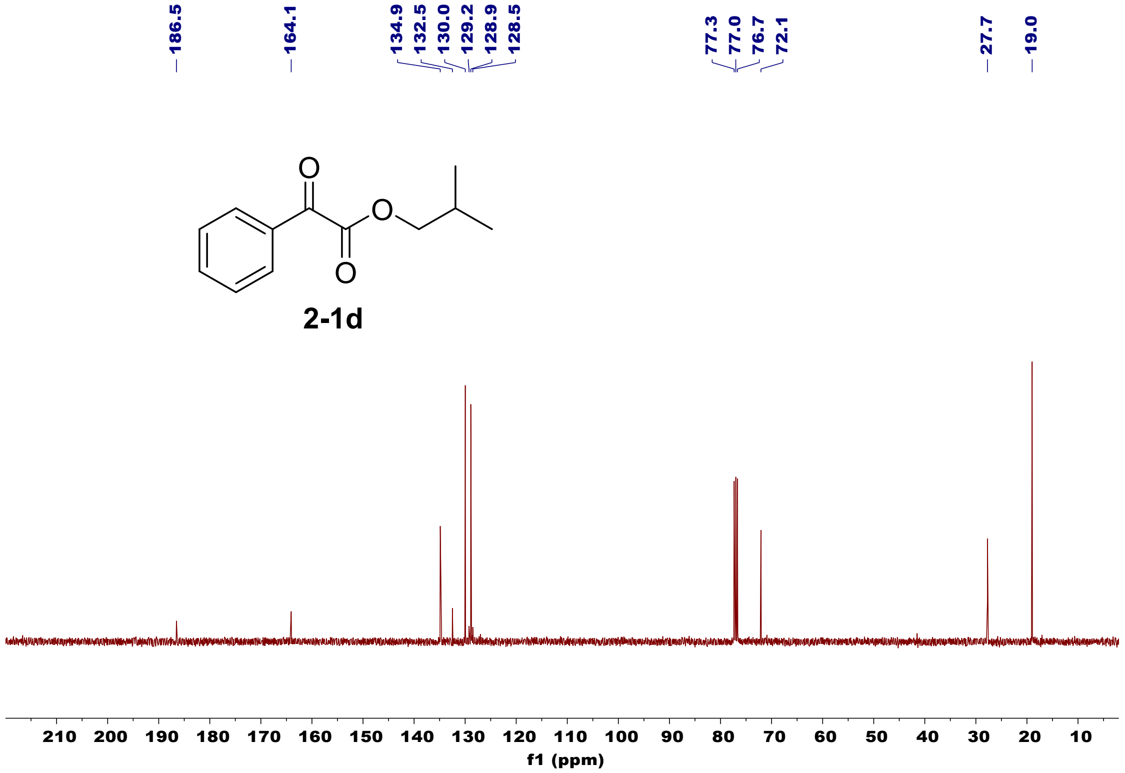
186.5
164.1
134.9 132.5 130.0 129.2 128.9 128.5
77.3 77.0 76.7 72.1
27.7
19.0
O
O
O
2-1d
210 200 190 180 170 160 150 140 130 120 110 100 90 80 70 60 50 40 30 20 10
f1 (ppm)

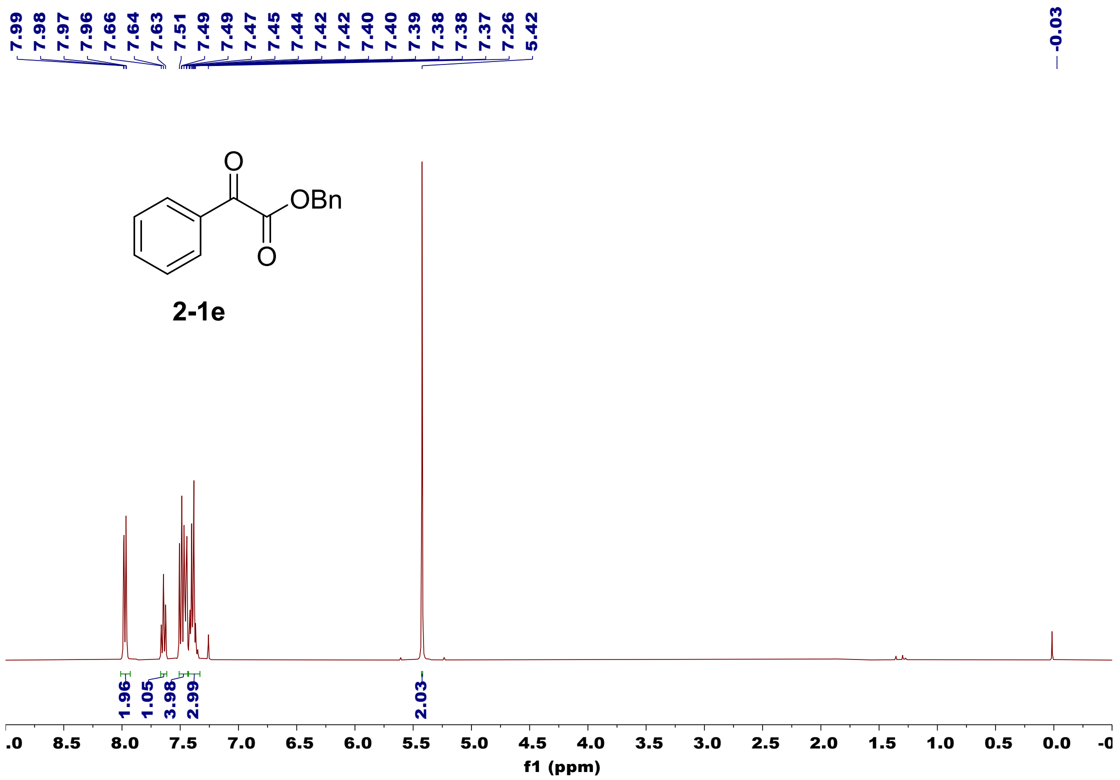
7.99
7.98
7.97
7.96
7.66
7.64
7.63
7.51
7.49
7.49
7.47
7.45
7.44
7.42
7.42
7.40
7.40
7.39
7.38
7.38
7.37
7.26
5.42
-0.03
OBn
O
O
1.96
1.05
3.98
2.99
2.03
f1 (ppm)

2-1e

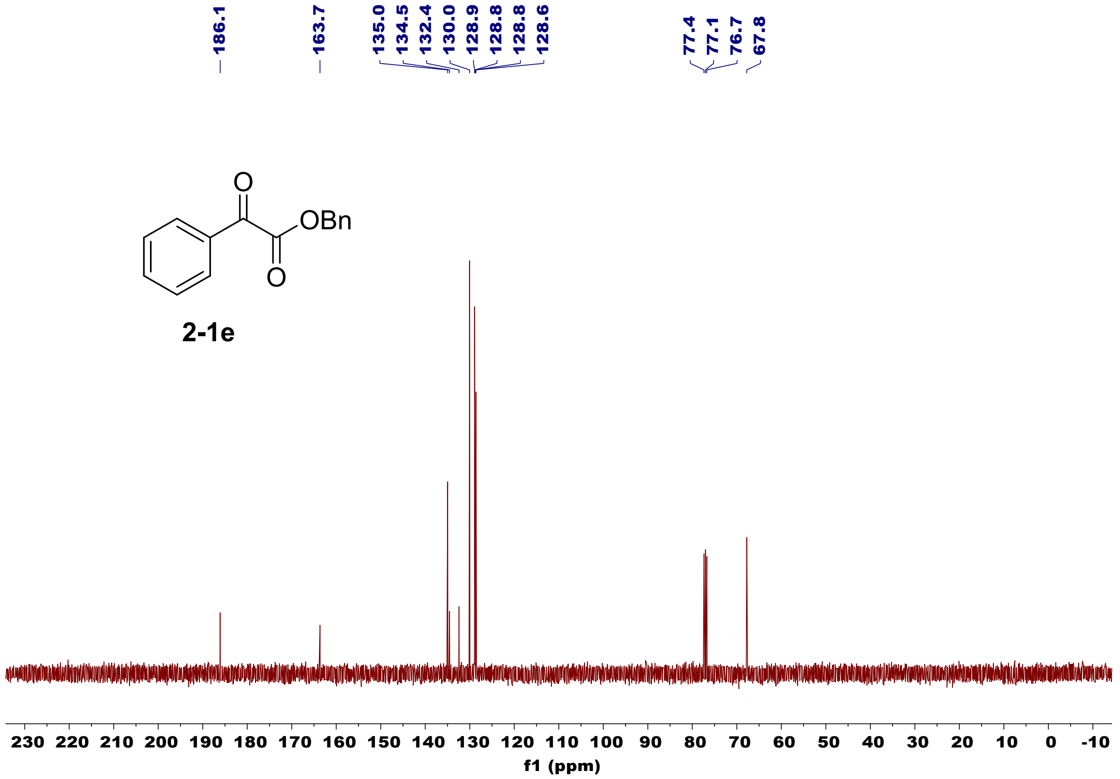
186.1
163.7
135.0
134.5
132.4
130.0
128.9
128.8
128.8
128.6
77.4
77.1
76.7
67.8
OBn
O
O
230 220 210 200 190 180 170 160 150 140 130 120 110 100 90 80 70 60 50 40 30 20 10 0 -10
f1 (ppm)

2-1e

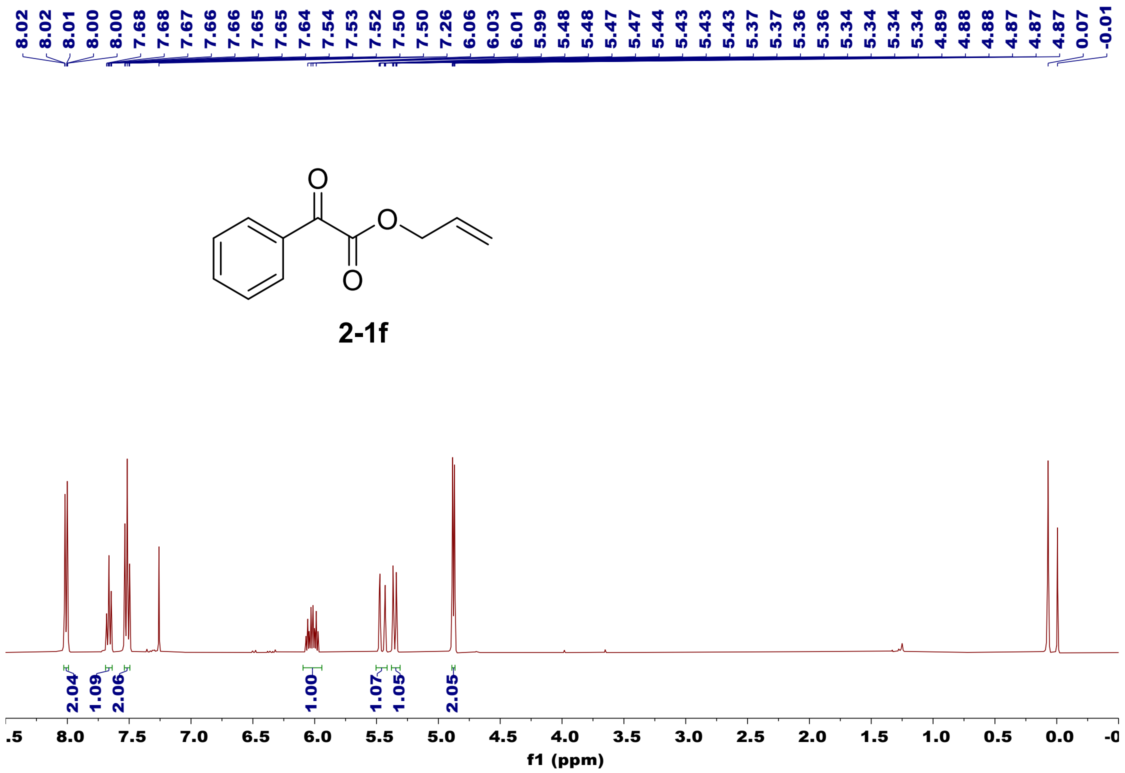
8.02 8.02 8.01 8.00 8.00 7.68 7.68 7.67 7.66 7.66 7.65 7.65 7.64 7.54 7.53 7.52 7.50 7.50 7.26 6.06 6.03 6.01 5.99 5.48 5.48 5.47 5.47 5.44 5.43 5.43 5.43 5.37 5.37 5.36 5.36 5.34 5.34 5.34 5.34 4.89 4.88 4.88 4.87 4.87 4.87 0.07 -0.01
O
O
O
2-1f
2.04 1.09 2.06 1.00 1.07 1.05 2.05
.5 8.0 7.5 7.0 6.5 6.0 5.5 5.0 4.5 4.0 3.5 3.0 2.5 2.0 1.5 1.0 0.5 0.0
f1 (ppm)

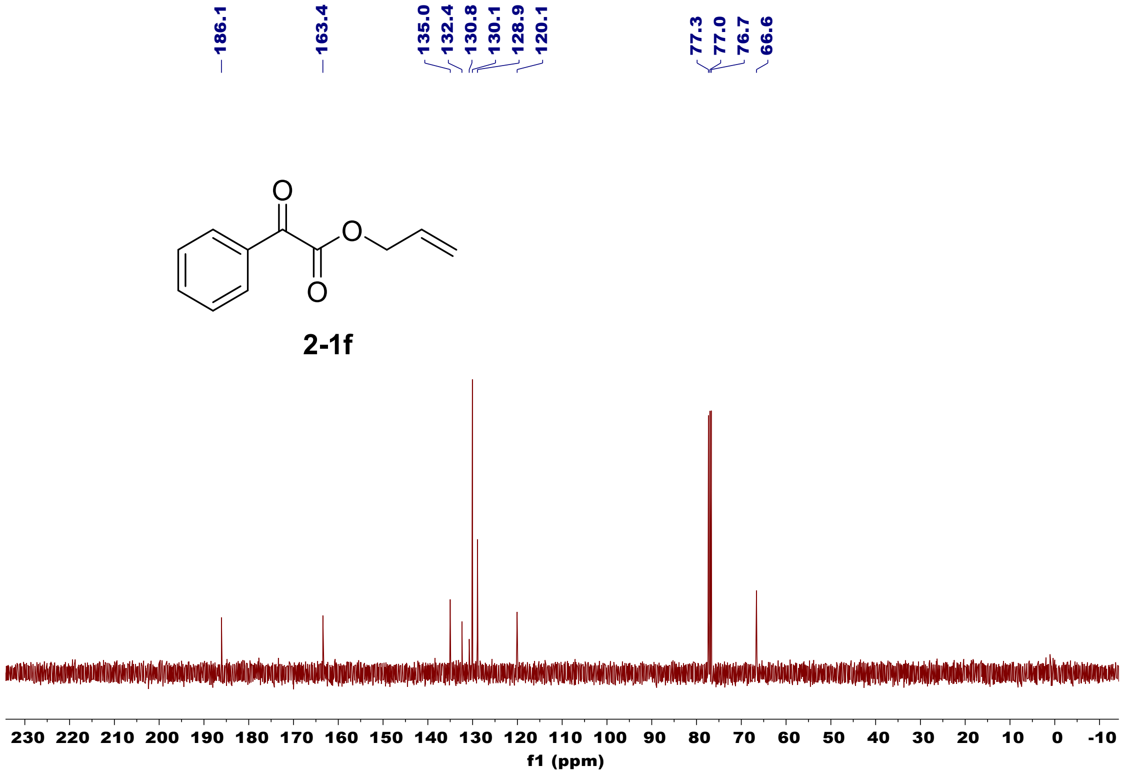
186.1
163.4
135.0 132.4 130.8 130.1 128.9 120.1
77.3 77.0 76.7 66.6
O
O
O
2-1f
230 220 210 200 190 180 170 160 150 140 130 120 110 100 90 80 70 60 50 40 30 20 10 0 -10
f1 (ppm)

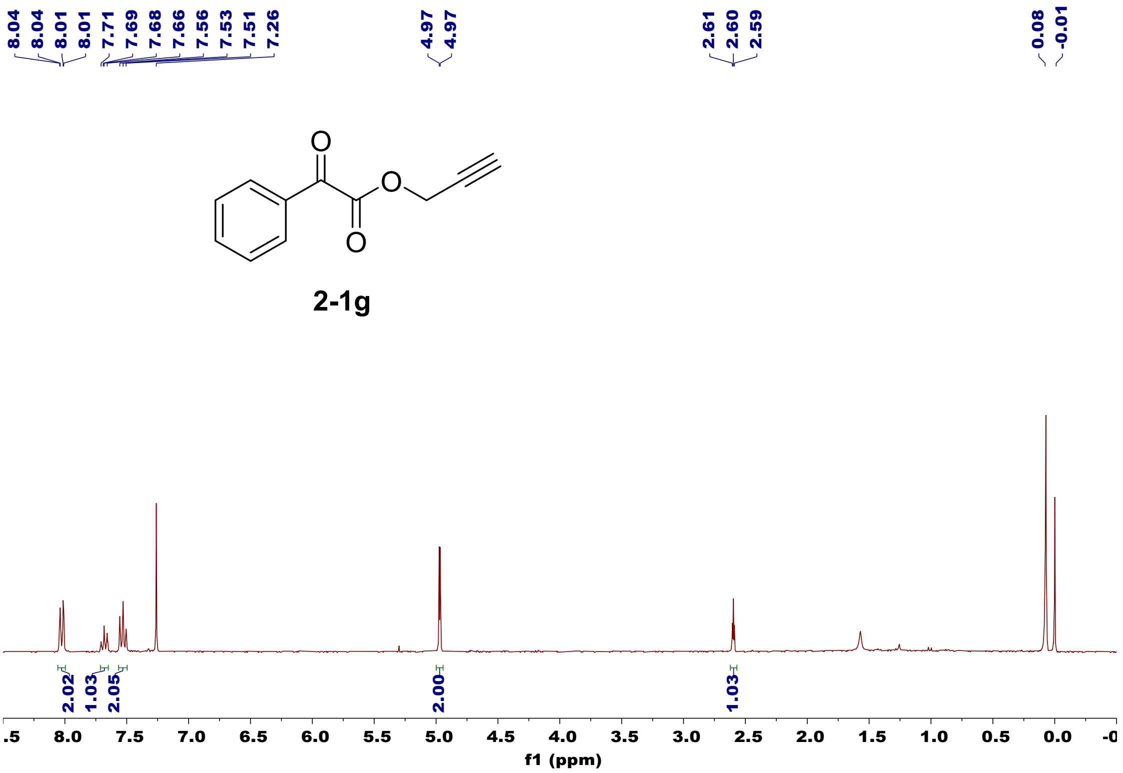
8.04
8.04
8.01
8.01
7.71
7.69
7.68
7.66
7.56
7.53
7.51
7.26
4.97
4.97
2.61
2.60
2.59
0.08
-0.01
O
O
O
2-1g
2.02
1.03
2.05
2.00
1.03
f1 (ppm)

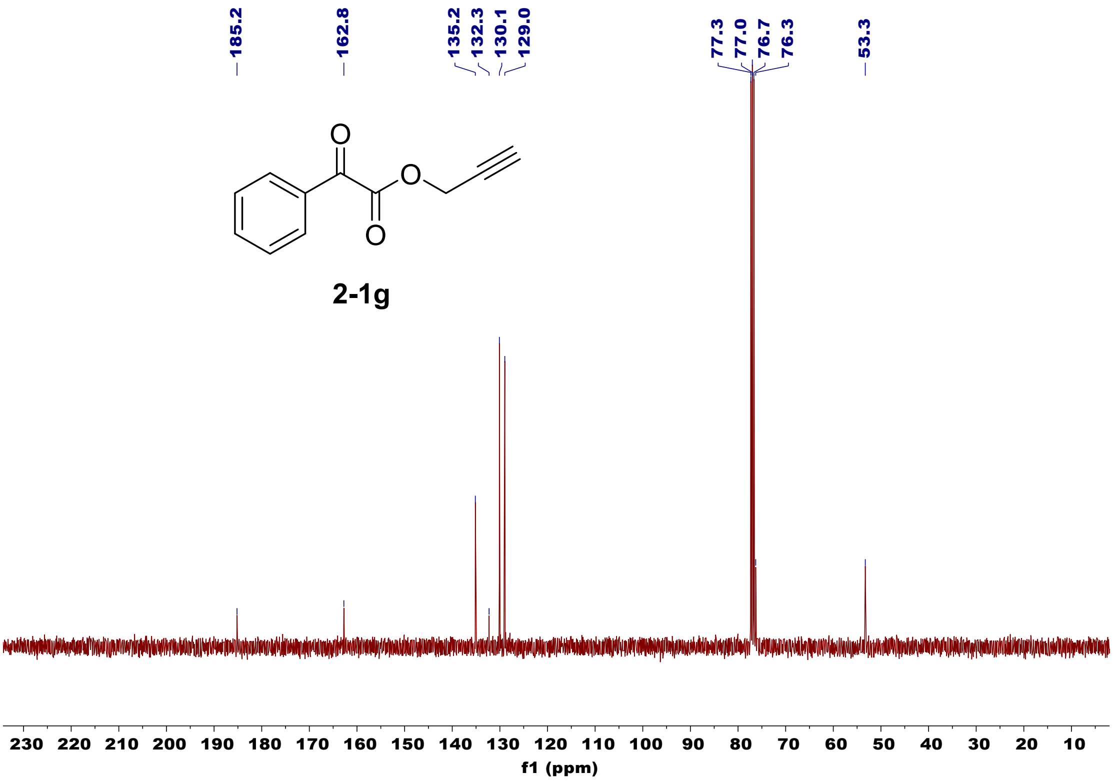
185.2
162.8
135.2
132.3
130.1
129.0
77.3
77.0
76.7
76.3
53.3
O
O
O
2-1g
f1 (ppm)

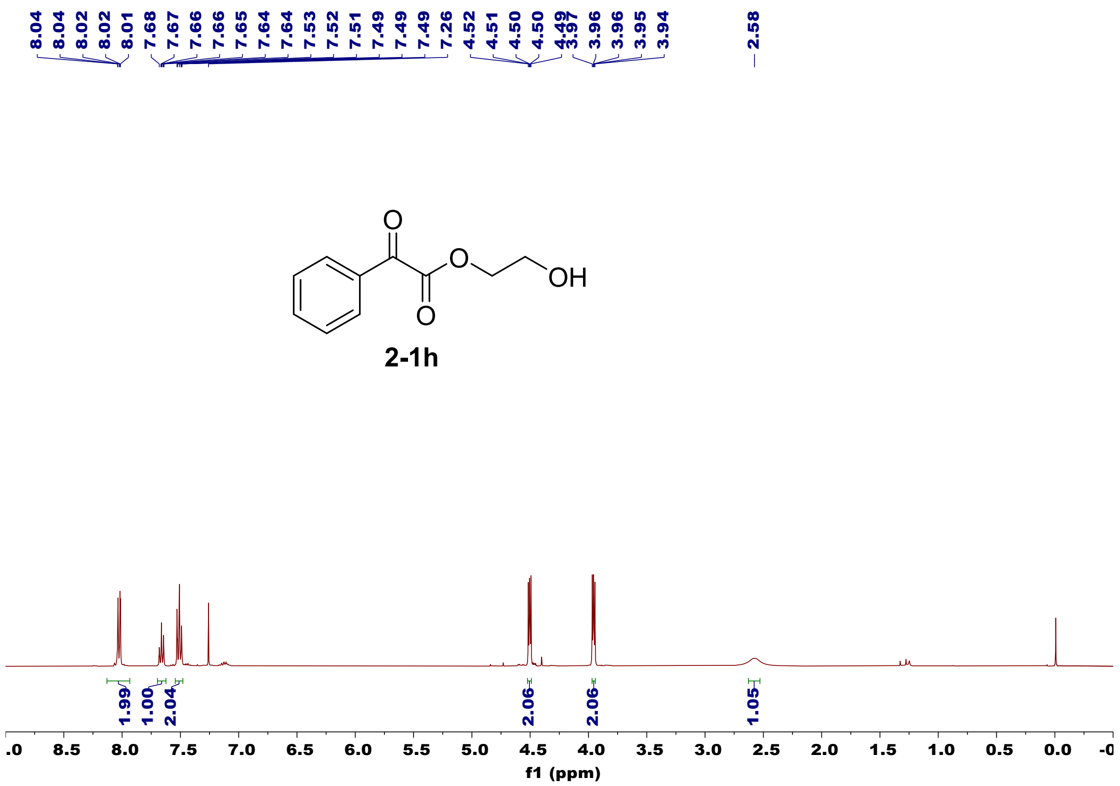
8.04 8.04 8.02 8.02 8.01 7.68 7.67 7.66 7.66 7.65 7.64 7.64 7.53 7.52 7.51 7.49 7.49 7.49 7.26 4.52 4.51 4.50 4.50 4.49 3.97 3.96 3.96 3.95 3.94
2.58
O
O
OH
O
2-1h
1.99
1.00
2.04
2.06
2.06
1.05
f1 (ppm)

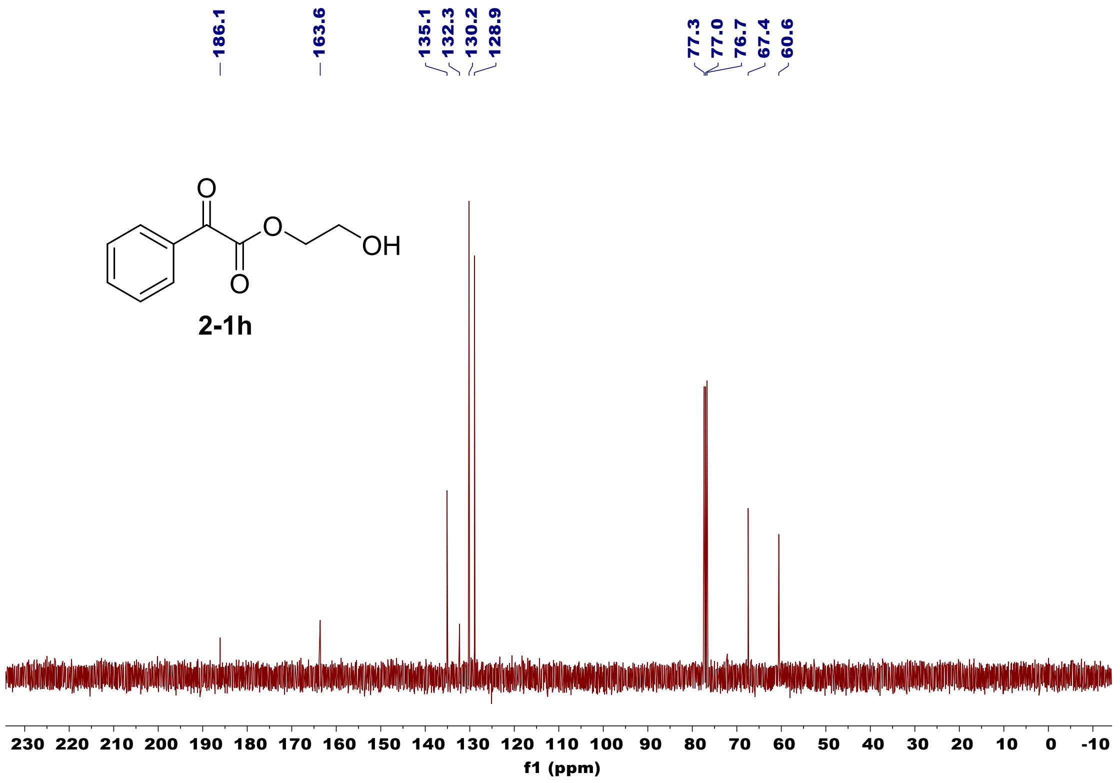
186.1
163.6
135.1 132.3 130.2 128.9
77.3 77.0 76.7 67.4 60.6
O
O
OH
O
2-1h
f1 (ppm)

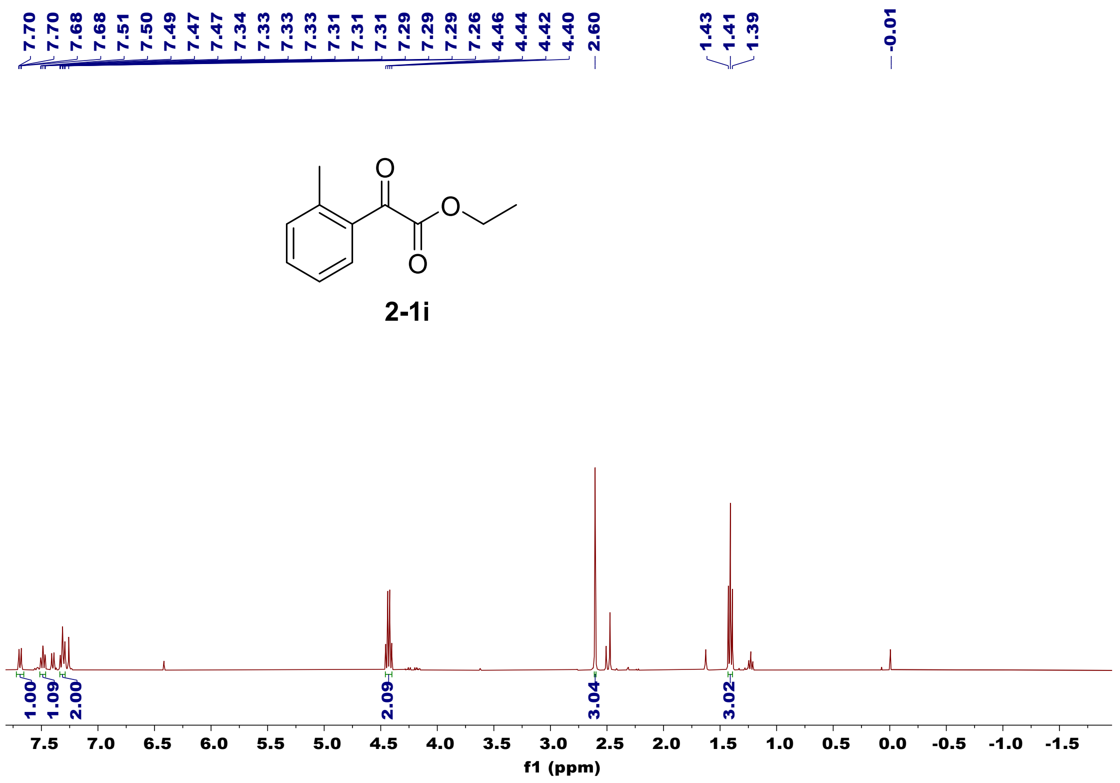
7.70 7.70 7.68 7.68 7.51 7.50 7.49 7.47 7.47 7.34 7.33 7.33 7.33 7.31 7.31 7.31 7.29 7.29 7.29 7.26 4.46 4.44 4.42 4.40 2.60
1.43 1.41 1.39
-0.01
O
O
O
2-1i
1.00 1.09 2.00
2.09
3.04
3.02
7.5 7.0 6.5 6.0 5.5 5.0 4.5 4.0 3.5 3.0 2.5 2.0 1.5 1.0 0.5 0.0 -0.5 -1.0 -1.5
f1 (ppm)

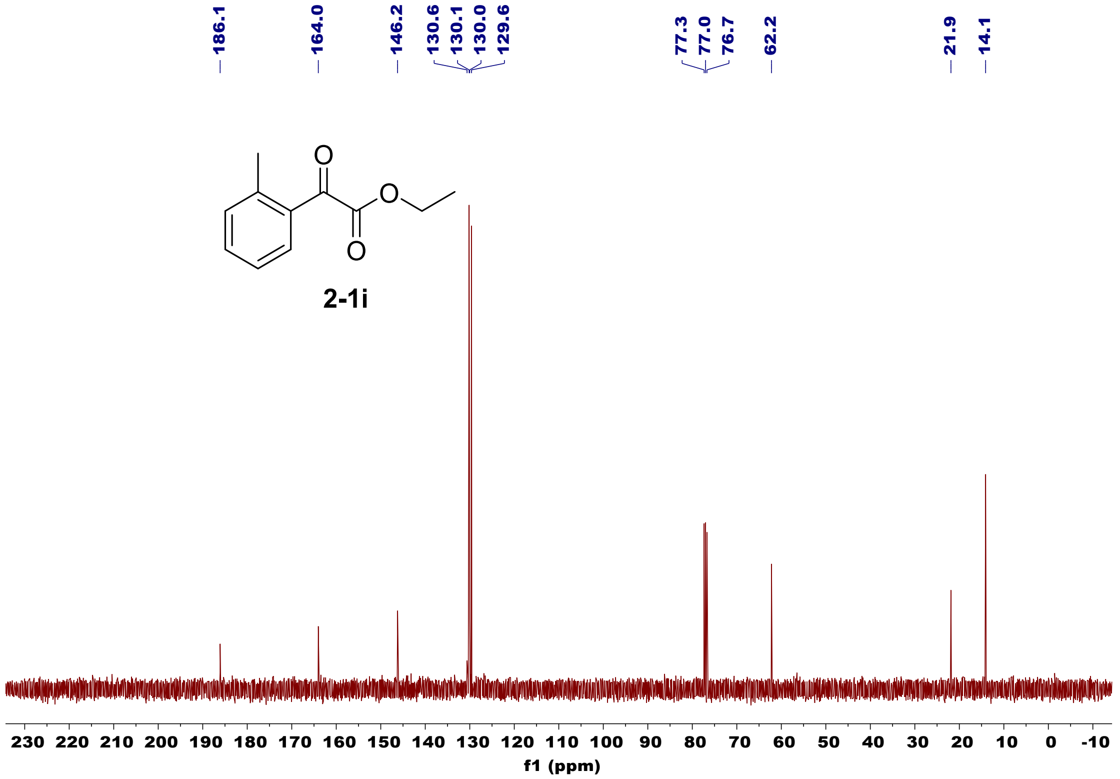
186.1
164.0
146.2
130.6 130.1 130.0 129.6
77.3 77.0 76.7
62.2
21.9 14.1
O
O
O
2-1i
230 220 210 200 190 180 170 160 150 140 130 120 110 100 90 80 70 60 50 40 30 20 10 0 -10
f1 (ppm)

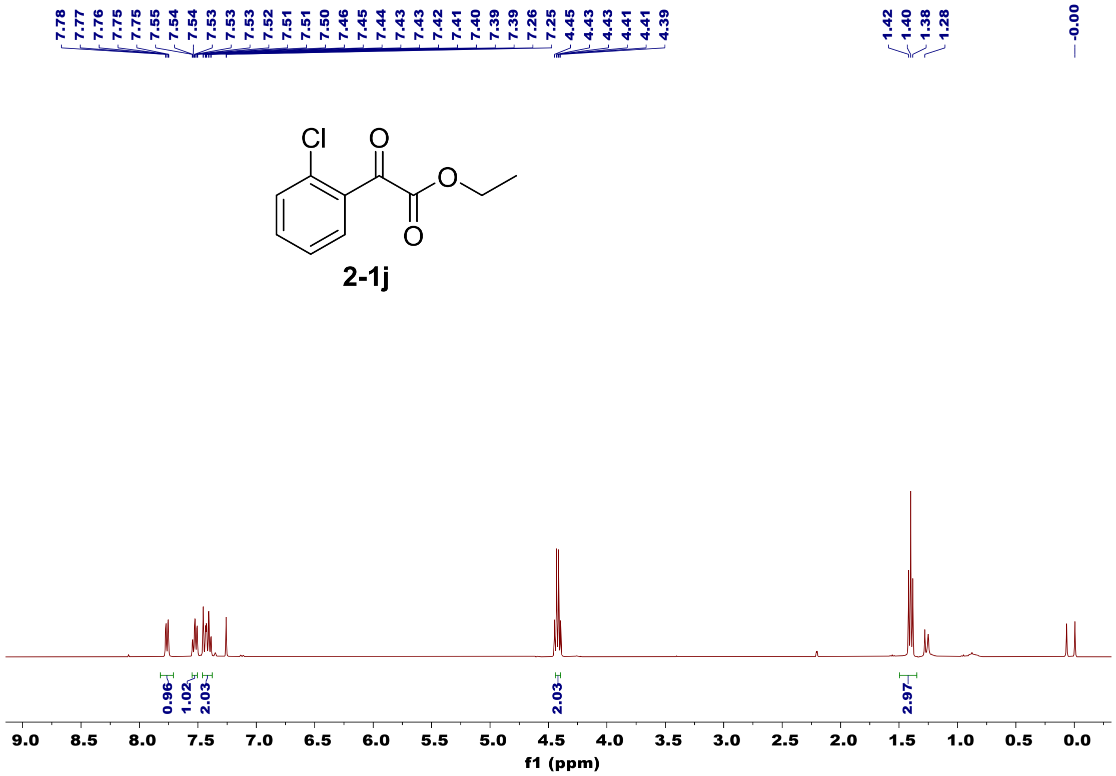
7.78 7.77 7.76 7.75 7.75 7.55 7.54 7.54 7.53 7.53 7.53 7.52 7.51 7.51 7.50 7.46 7.45 7.44 7.43 7.43 7.42 7.41 7.40 7.39 7.39 7.26 7.25 4.45 4.43 4.43 4.41 4.41 4.41 4.39
1.42 1.40 1.38 1.28
-0.00
Cl
O
O
O
2-1j
0.96
1.02
2.03
2.03
2.97
9.0 8.5 8.0 7.5 7.0 6.5 6.0 5.5 5.0 4.5 4.0 3.5 3.0 2.5 2.0 1.5 1.0 0.5 0.0
f1 (ppm)

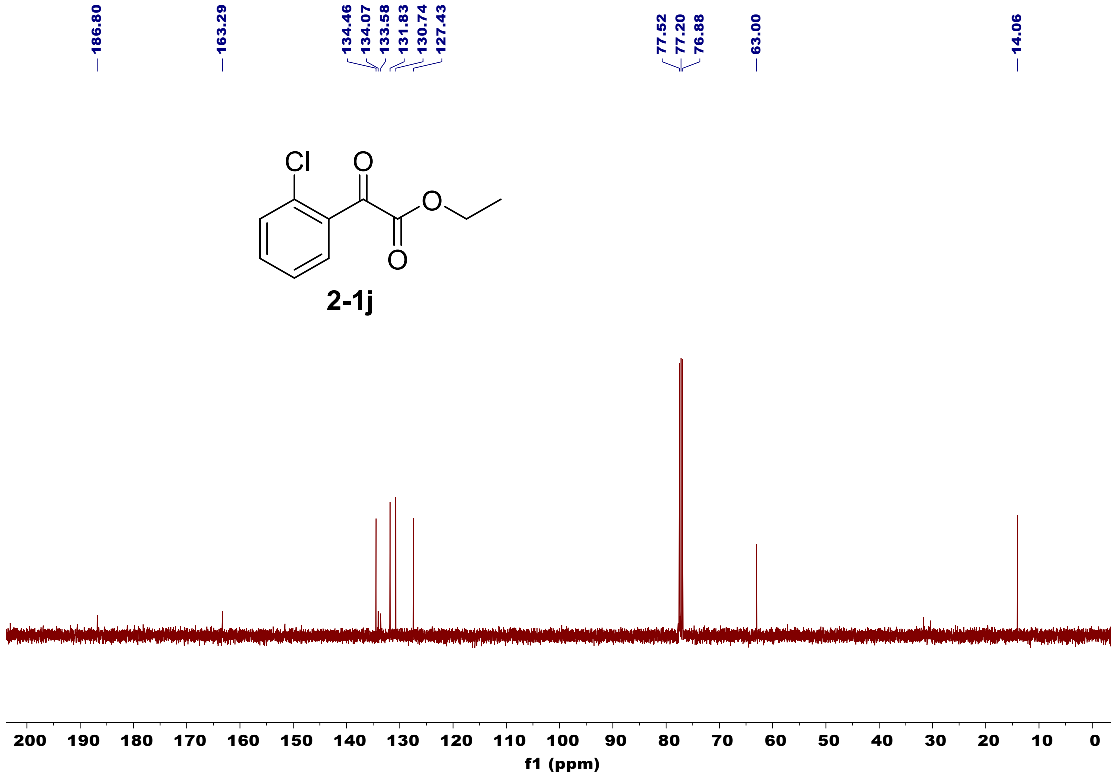
186.80
163.29
134.46 134.07 133.58 131.83 130.74 127.43
77.52 77.20 76.88
63.00
14.06
Cl
O
O
O
2-1j
200 190 180 170 160 150 140 130 120 110 100 90 80 70 60 50 40 30 20 10 0
f1 (ppm)

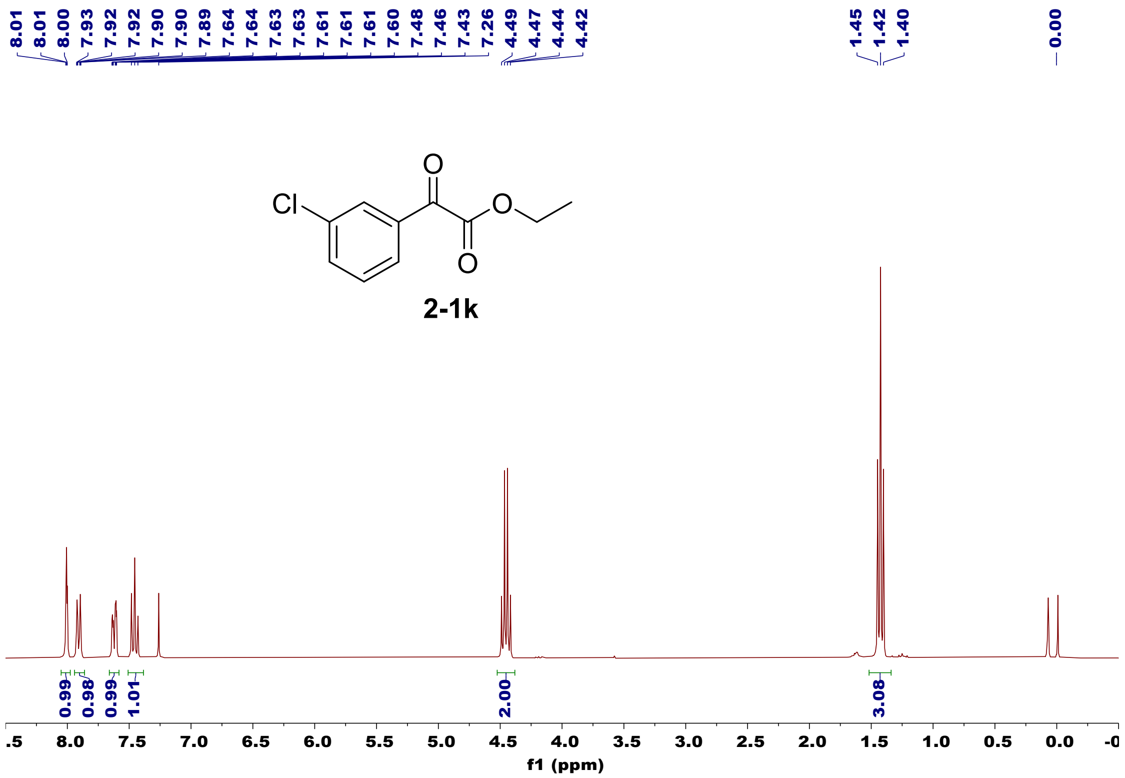
8.01
8.01
8.00
7.93
7.92
7.92
7.90
7.90
7.89
7.64
7.64
7.63
7.63
7.61
7.61
7.61
7.60
7.48
7.46
7.43
7.26
4.49
4.47
4.44
4.42
1.45
1.42
1.40
0.00
Cl
O
O
O
2-1k
0.99
0.98
0.99
1.01
2.00
3.08
f1 (ppm)

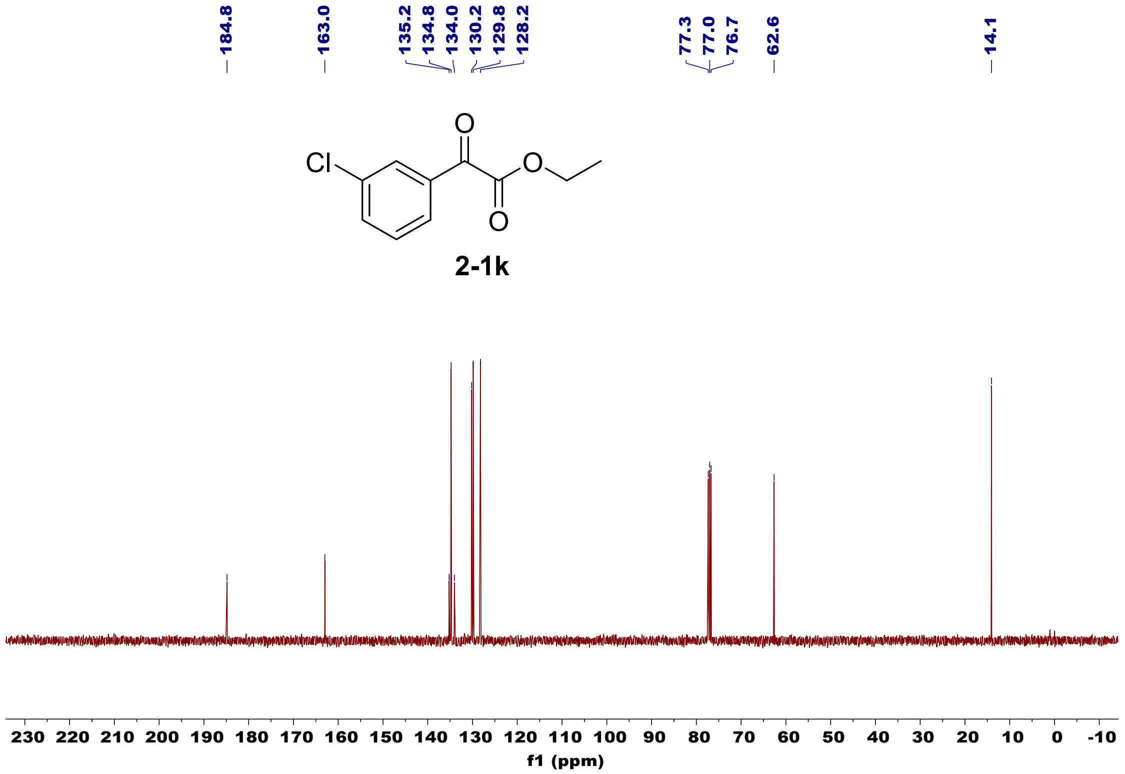
184.8
163.0
135.2
134.8
134.0
130.2
129.8
128.2
77.3
77.0
76.7
62.6
14.1
Cl
O
O
O
2-1k
f1 (ppm)

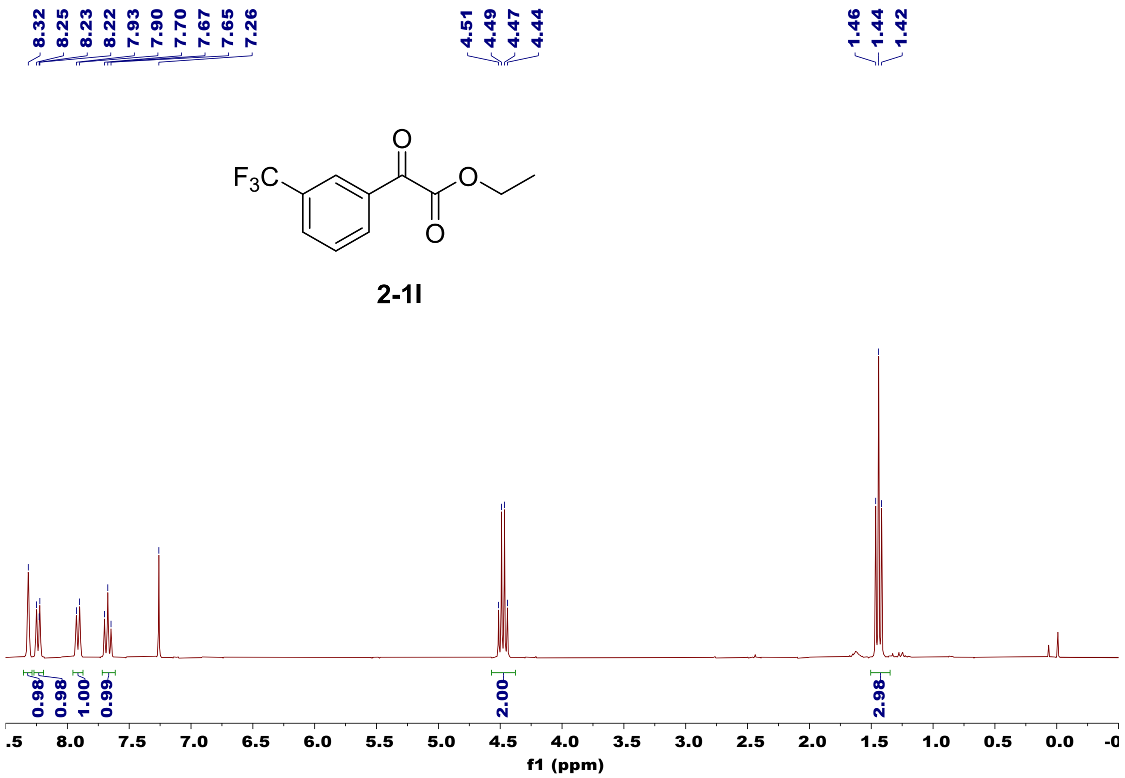
8.32 8.25 8.23 8.22 7.93 7.90 7.70 7.67 7.65 7.26
4.51 4.49 4.47 4.44
1.46 1.44 1.42
F3C
O
O
O
2-1l
0.98 0.98 1.00 0.99
2.00
2.98
f1 (ppm)

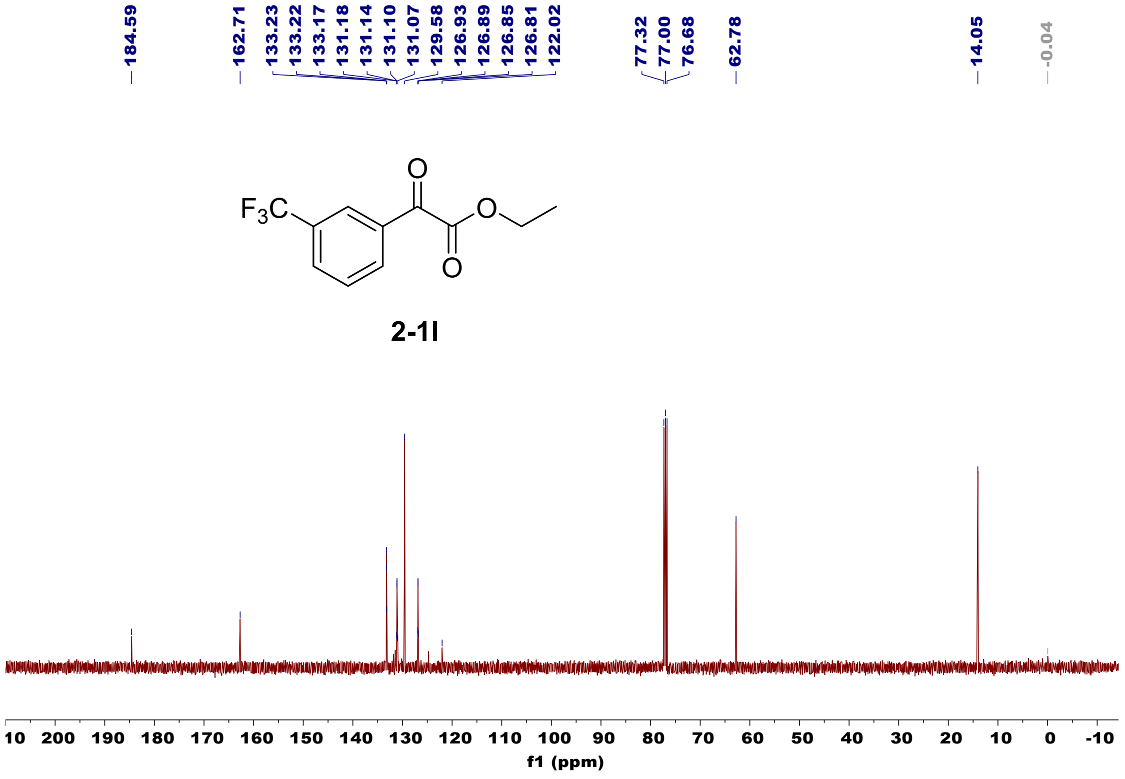
184.59
162.71
133.23 133.22 133.17 131.18 131.14 131.10 131.07 129.58 126.93 126.89 126.85 126.81 122.02
77.32 77.00 76.68
62.78
14.05
-0.04
F3C
O
O
O
2-1l
f1 (ppm)

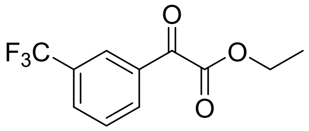

2-1l

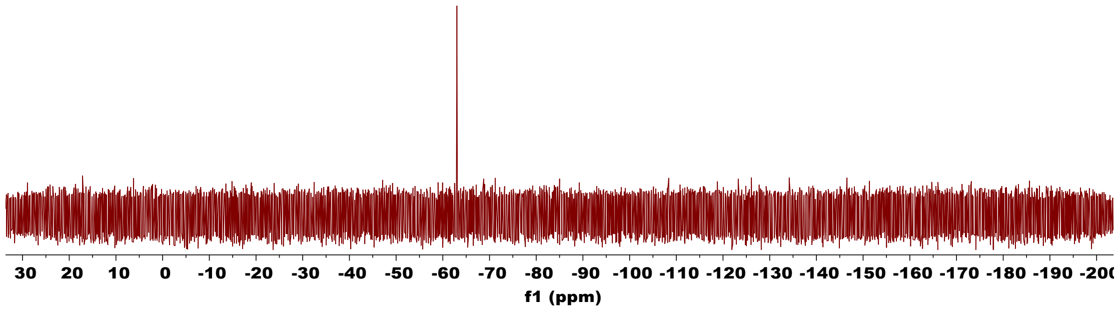
-63.0
30 20 10 0 -10 -20 -30 -40 -50 -60 -70 -80 -90 -100 -110 -120 -130 -140 -150 -160 -170 -180 -190 -200
f1 (ppm)

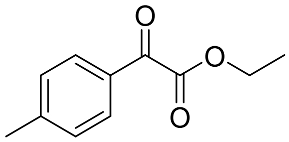

2-1m

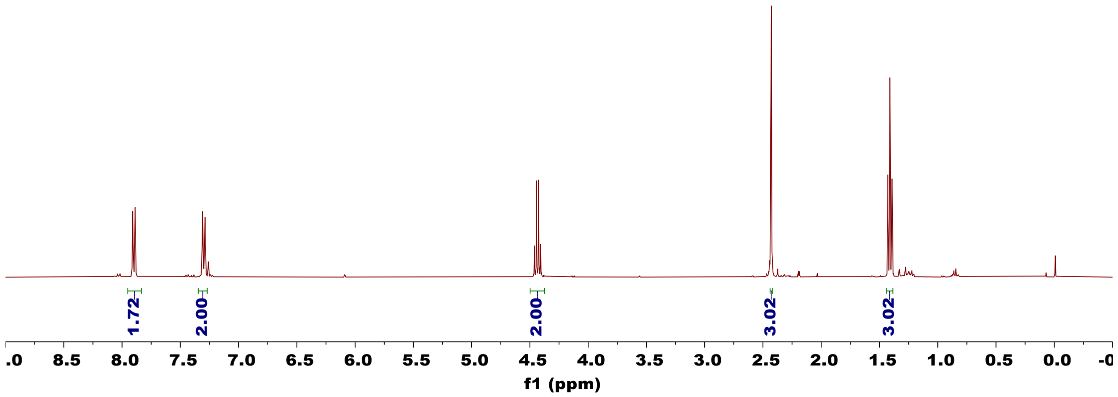
7.91 7.89 7.31 7.29 7.26 4.46 4.44 4.43 4.41 2.43 1.43 1.41 1.39
1.72 2.00 2.00 3.02 3.02
8.5 8.0 7.5 7.0 6.5 6.0 5.5 5.0 4.5 4.0 3.5 3.0 2.5 2.0 1.5 1.0 0.5 0.0
f1 (ppm)

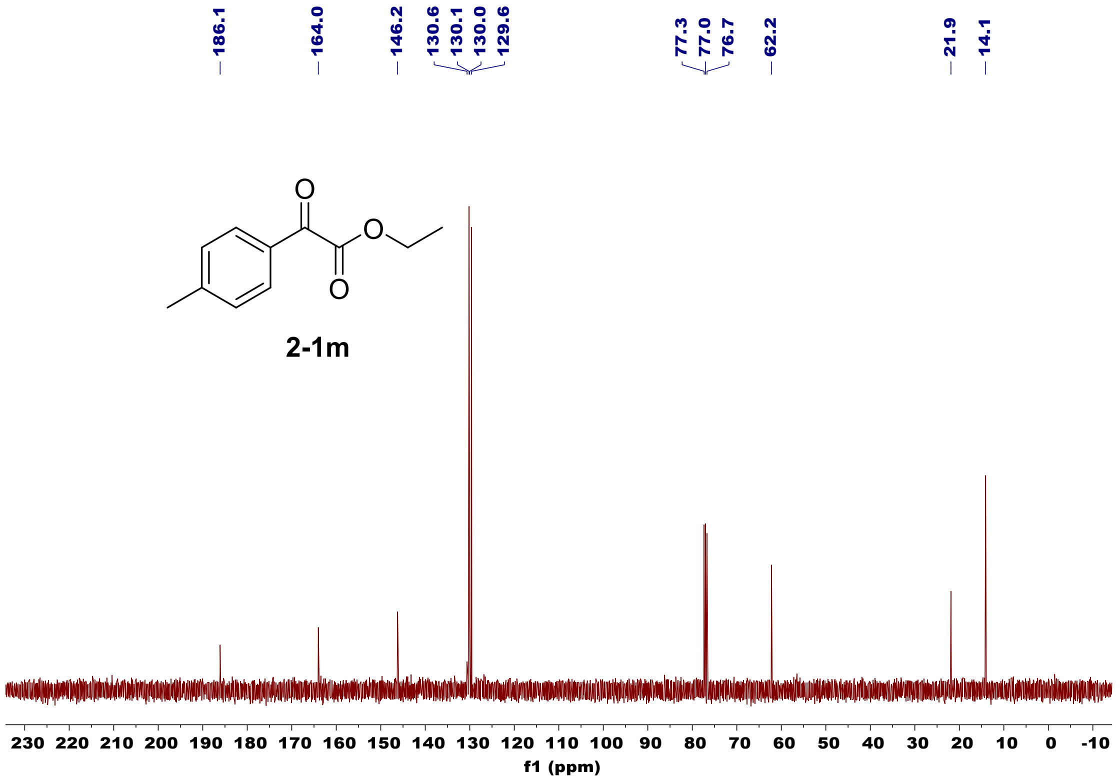
186.1
164.0
146.2
130.6
130.1
130.0
129.6
77.3
77.0
76.7
62.2
21.9
14.1
O
O
O
2-1m
230 220 210 200 190 180 170 160 150 140 130 120 110 100 90 80 70 60 50 40 30 20 10 0 -10
f1 (ppm)

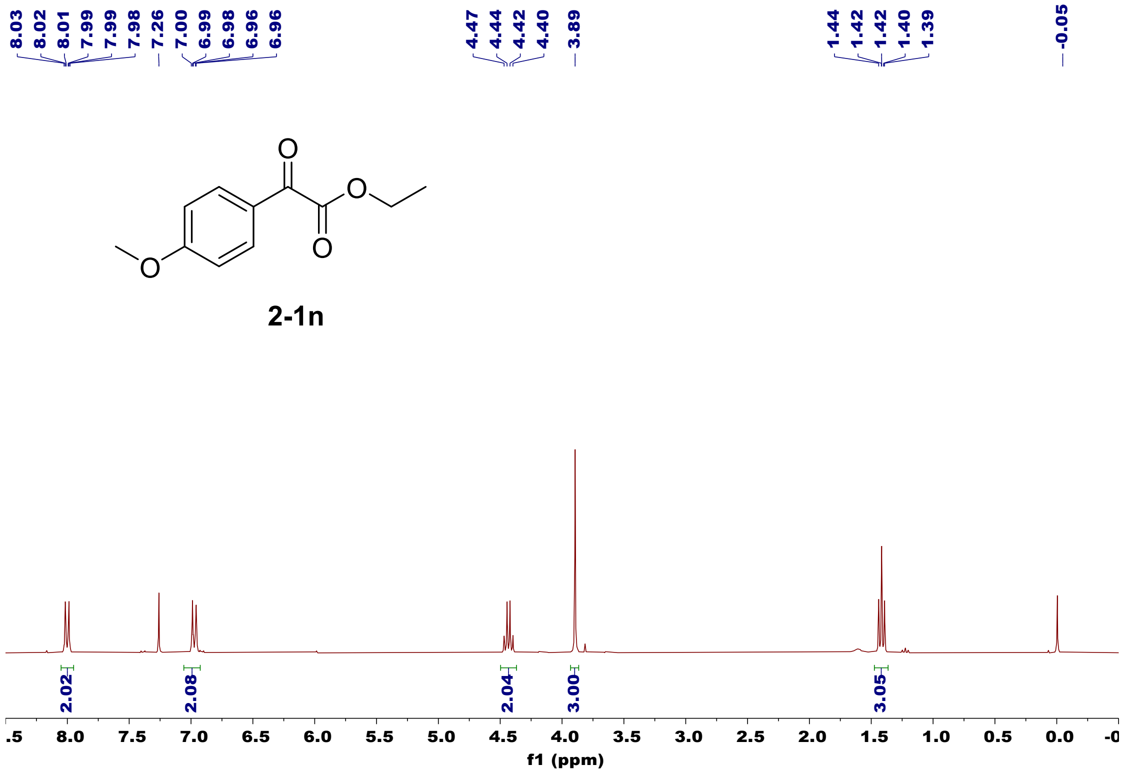
8.03
8.02
8.01
7.99
7.99
7.98
7.26
7.00
6.99
6.98
6.96
6.96
4.47
4.44
4.42
4.40
3.89
1.44
1.42
1.42
1.40
1.39
-0.05
O
O
O
O
2-1n
2.02
2.08
2.04
3.00
3.05
.5 8.0 7.5 7.0 6.5 6.0 5.5 5.0 4.5 4.0 3.5 3.0 2.5 2.0 1.5 1.0 0.5 0.0 -0
f1 (ppm)

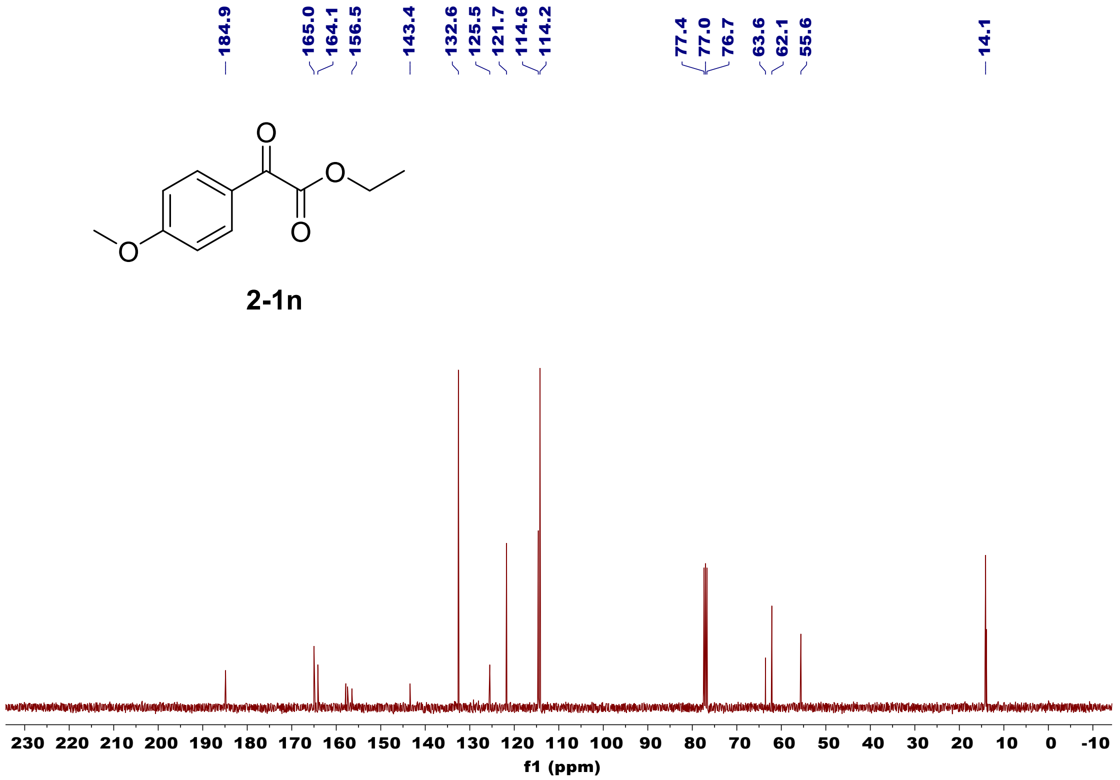
184.9
165.0
164.1
156.5
143.4
132.6
125.5
121.7
114.6
114.2
77.4
77.0
76.7
63.6
62.1
55.6
14.1
230 220 210 200 190 180 170 160 150 140 130 120 110 100 90 80 70 60 50 40 30 20 10 0 -10
f1 (ppm)

2-1n

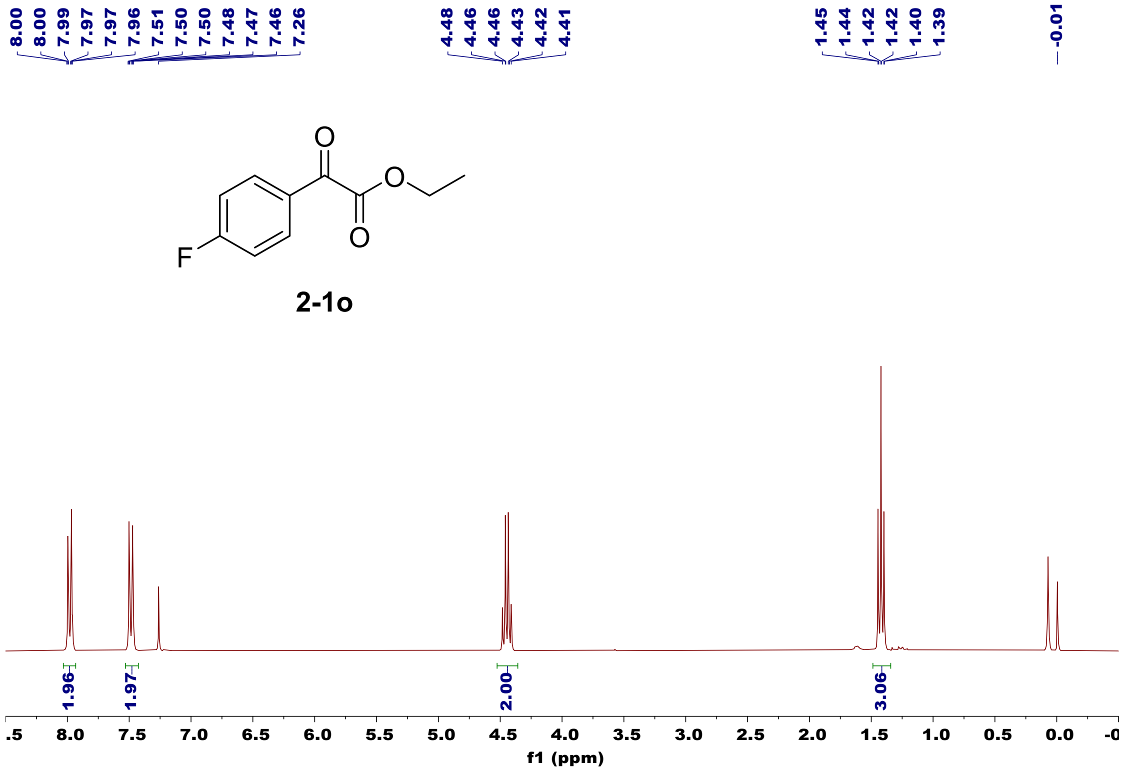
8.00
8.00
7.99
7.97
7.97
7.96
7.51
7.50
7.50
7.48
7.47
7.46
7.26
4.48
4.46
4.46
4.43
4.42
4.41
1.45
1.44
1.42
1.42
1.40
1.39
-0.01
F
1.96
1.97
2.00
3.06
.5 8.0 7.5 7.0 6.5 6.0 5.5 5.0 4.5 4.0 3.5 3.0 2.5 2.0 1.5 1.0 0.5 0.0 -0
f1 (ppm)

2-1o

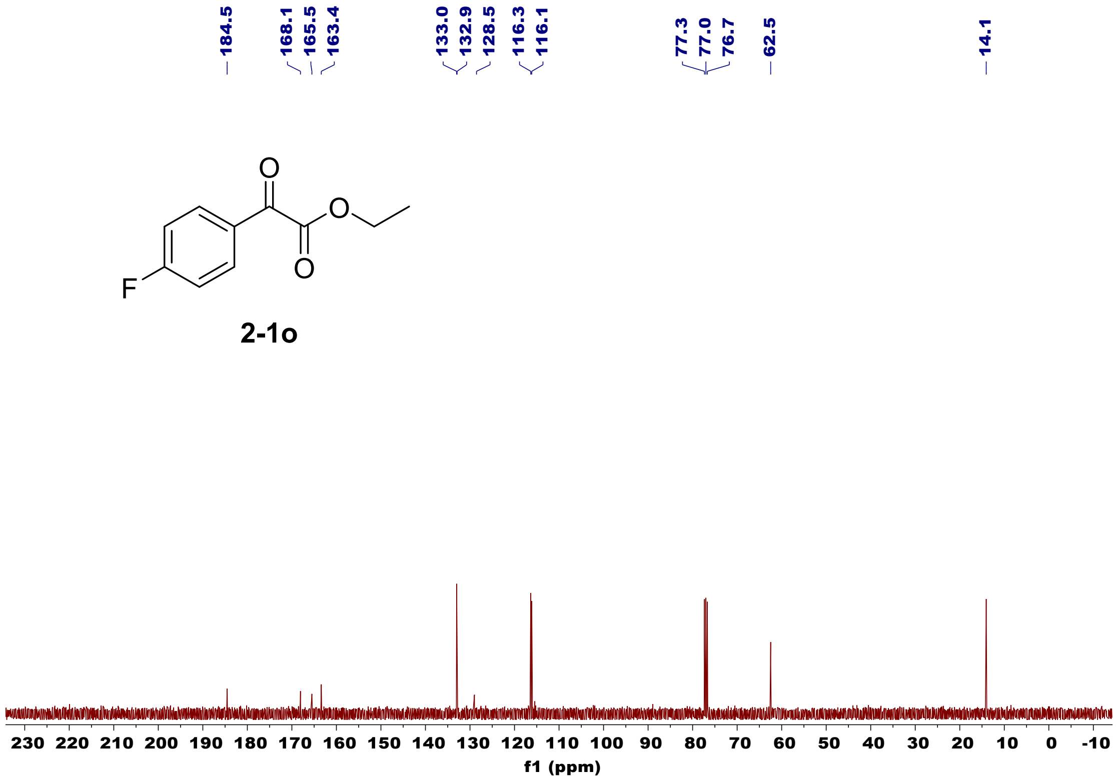
184.5
168.1
165.5
163.4
133.0
132.9
128.5
116.3
116.1
77.3
77.0
76.7
62.5
14.1
F
O
O
O
2-1o
230 220 210 200 190 180 170 160 150 140 130 120 110 100 90 80 70 60 50 40 30 20 10 0 -10
f1 (ppm)

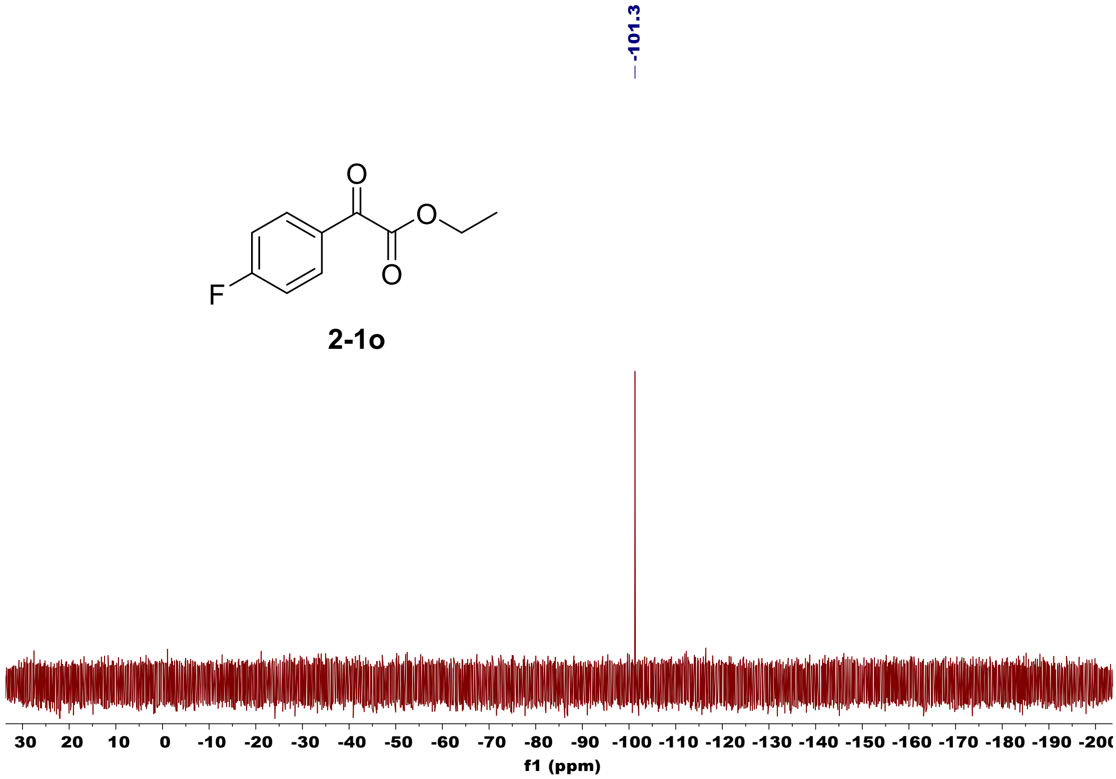
-101.3
F
O
O
O
2-1o
30 20 10 0 -10 -20 -30 -40 -50 -60 -70 -80 -90 -100 -110 -120 -130 -140 -150 -160 -170 -180 -190 -200
f1 (ppm)

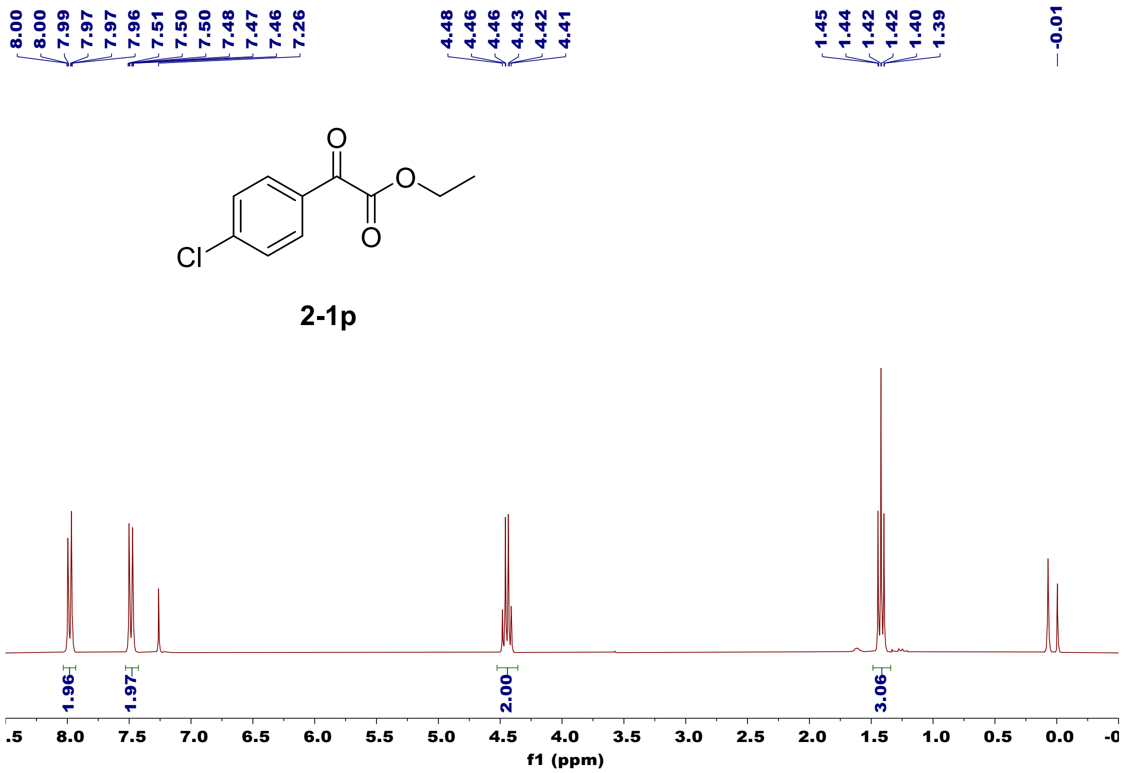
8.00 8.00 7.99 7.97 7.97 7.96 7.51 7.50 7.50 7.48 7.47 7.46 7.26
4.48 4.46 4.46 4.43 4.42 4.41
1.45 1.44 1.42 1.42 1.40 1.39
-0.01
O
O
O
Cl
2-1p
1.96
1.97
2.00
3.06
f1 (ppm)

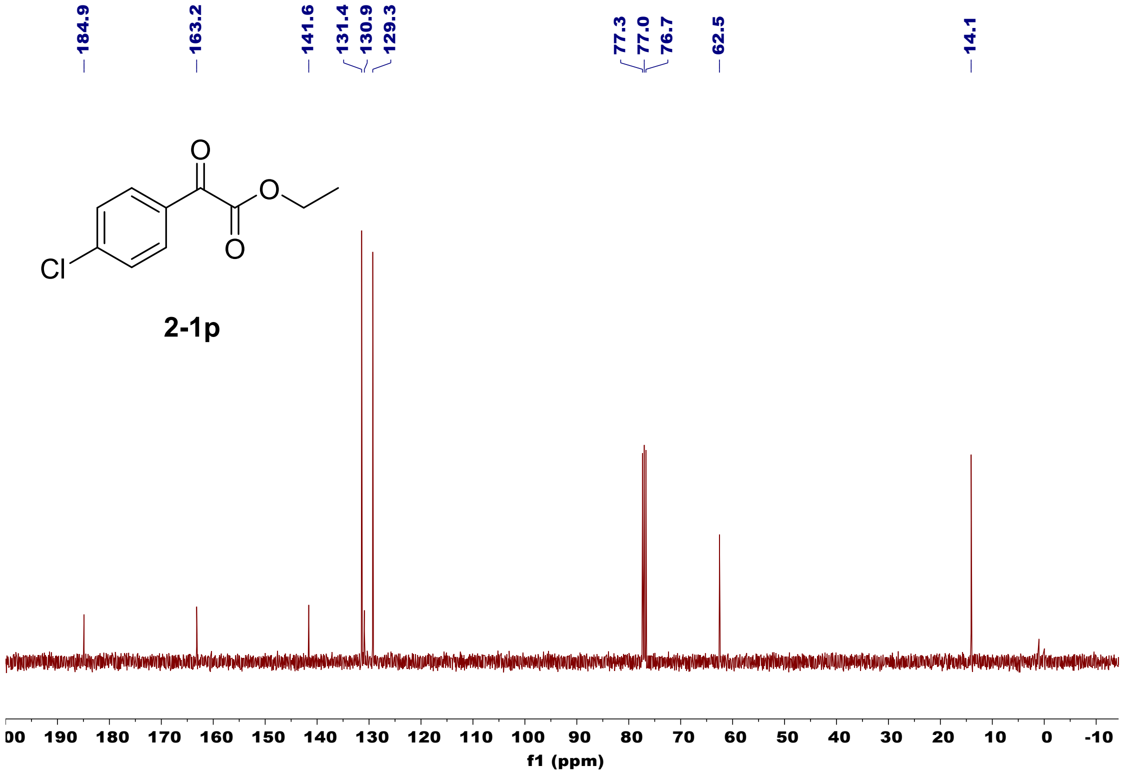
184.9
163.2
141.6 131.4 130.9 129.3
77.3 77.0 76.7
62.5
14.1
O
O
O
Cl
2-1p
f1 (ppm)

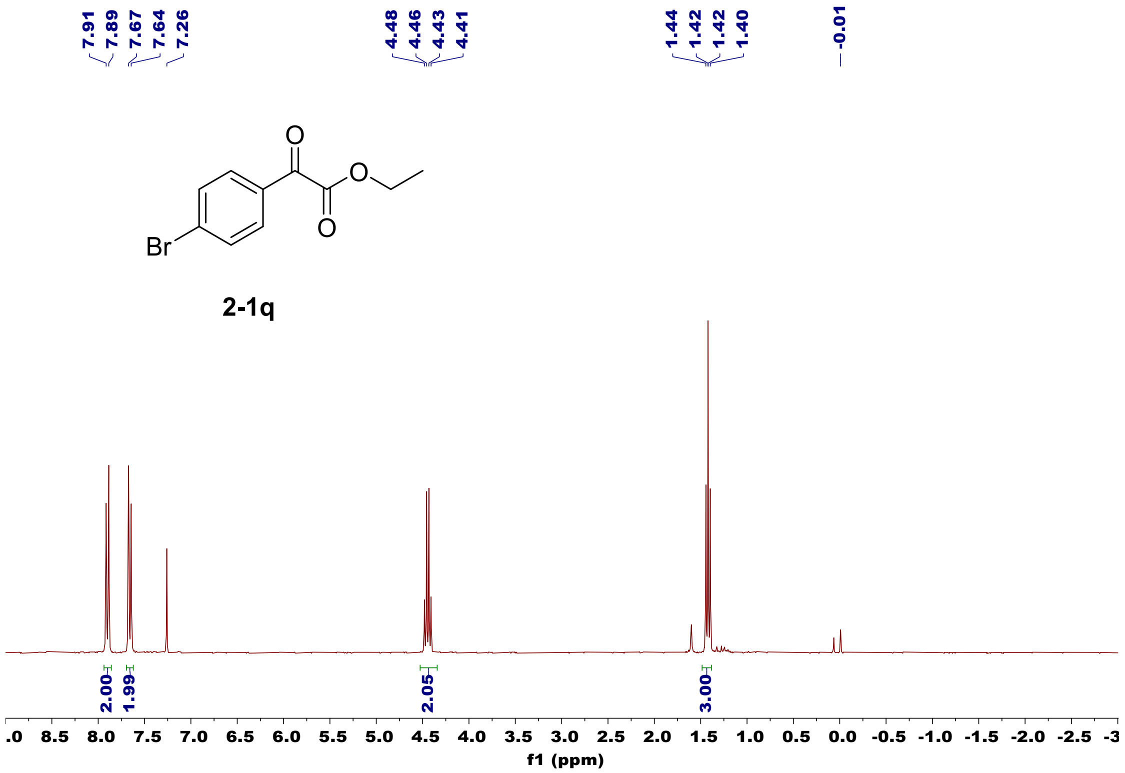
7.91
7.89
7.67
7.64
7.26
4.48
4.46
4.43
4.41
1.44
1.42
1.42
1.40
-0.01
Br
O
O
O
2-1q
2.00
1.99
2.05
3.00
.0 8.5 8.0 7.5 7.0 6.5 6.0 5.5 5.0 4.5 4.0 3.5 3.0 2.5 2.0 1.5 1.0 0.5 0.0 -0.5 -1.0 -1.5 -2.0 -2.5 -3
f1 (ppm)

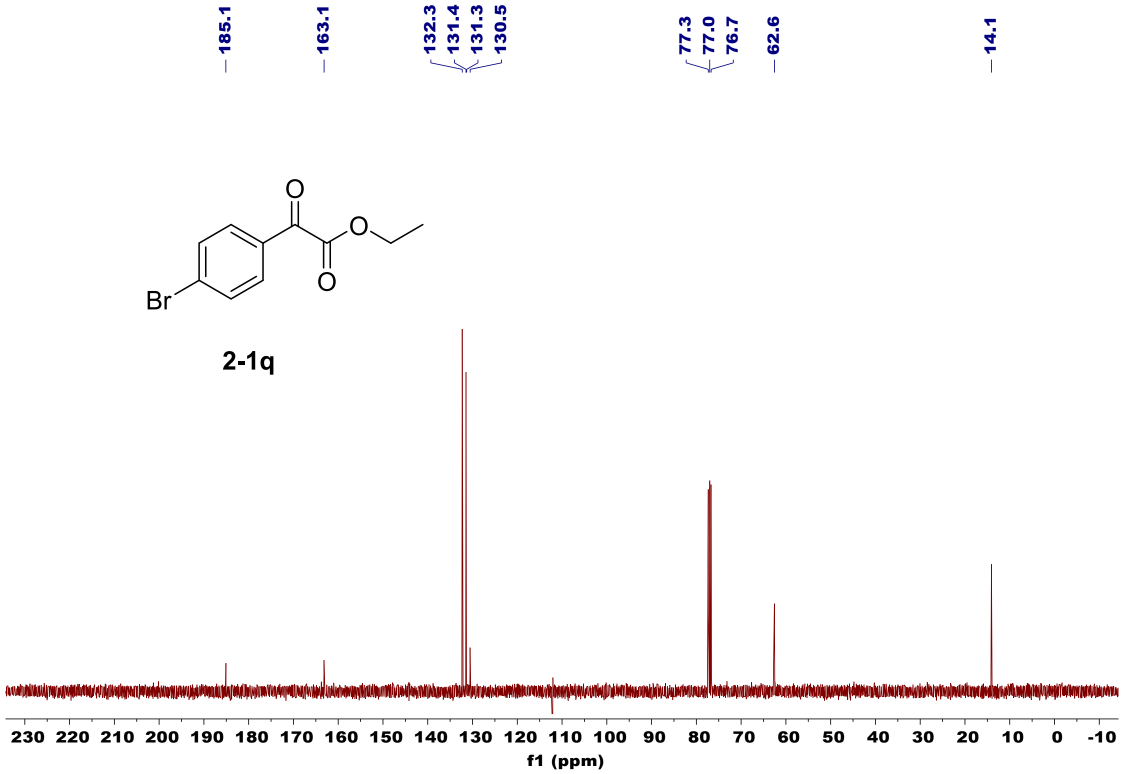
185.1
163.1
132.3
131.4
131.3
130.5
77.3
77.0
76.7
62.6
14.1
Br
O
O
O
2-1q
230 220 210 200 190 180 170 160 150 140 130 120 110 100 90 80 70 60 50 40 30 20 10 0 -10
f1 (ppm)

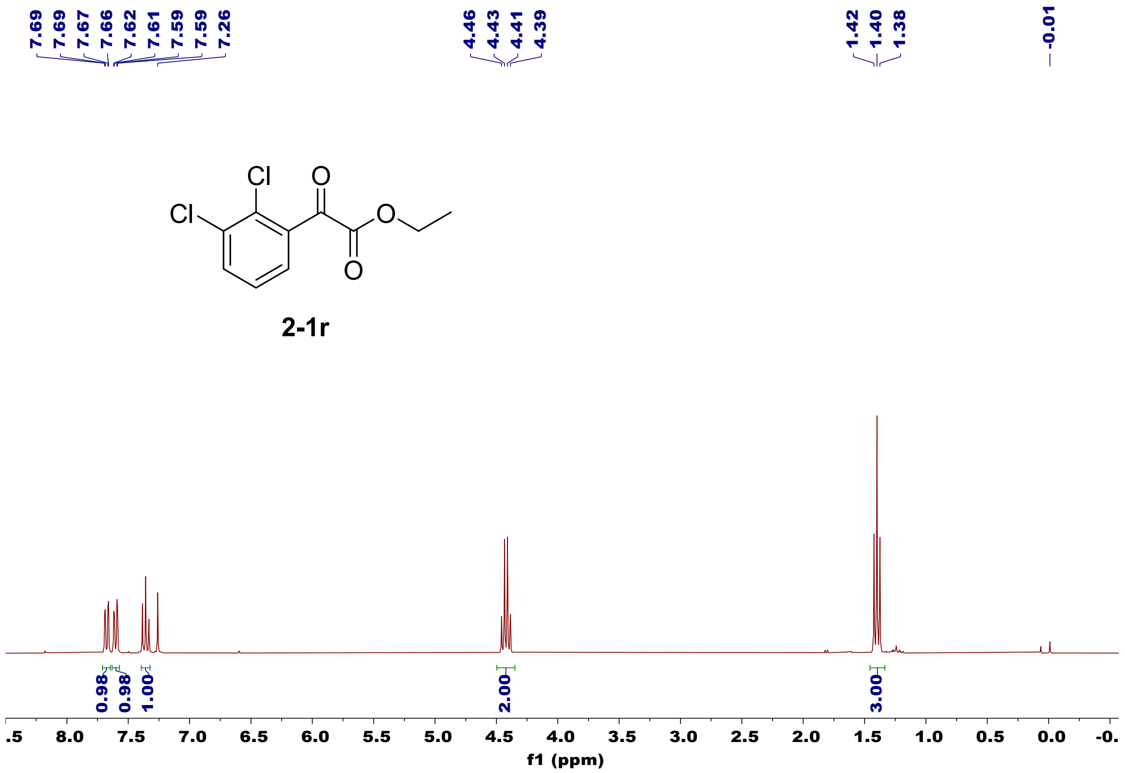
7.69
7.69
7.67
7.66
7.62
7.61
7.59
7.59
7.26
4.46
4.43
4.41
4.39
1.42
1.40
1.38
-0.01
Cl
Cl
O
O
O
2-1r
0.98
0.98
1.00
2.00
3.00
f1 (ppm)

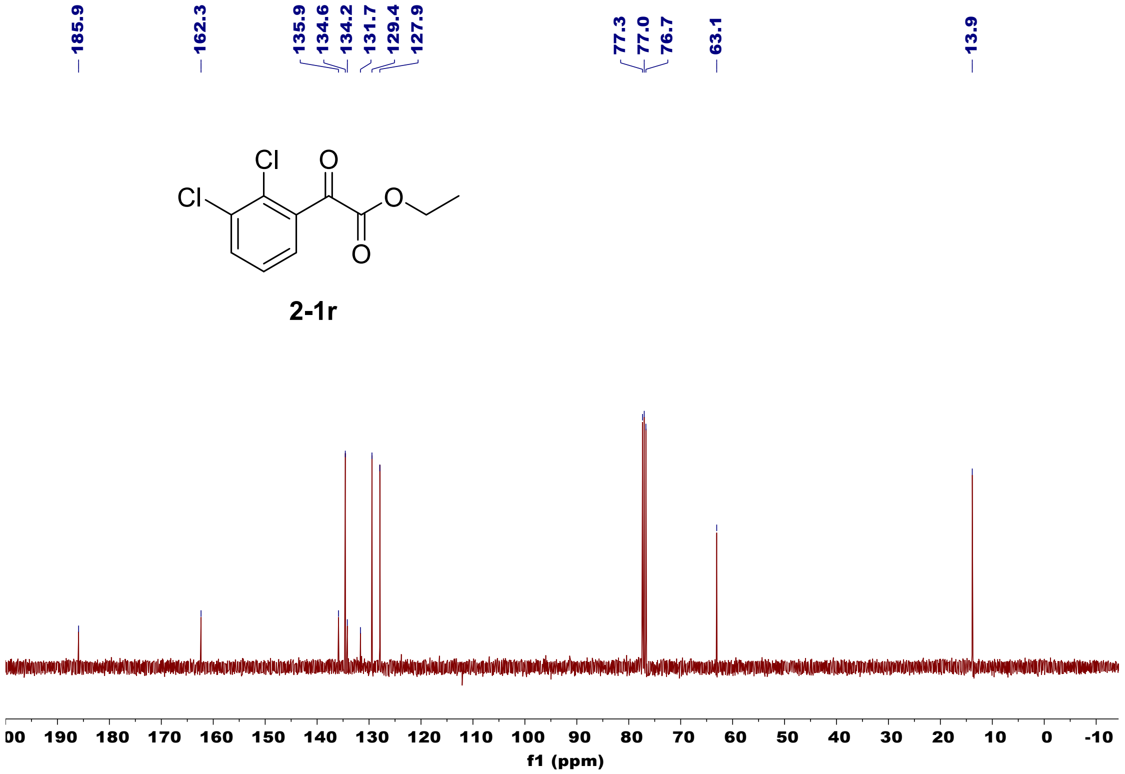
185.9
162.3
135.9
134.6
134.2
131.7
129.4
127.9
77.3
77.0
76.7
63.1
13.9
Cl
Cl
O
O
O
2-1r
f1 (ppm)

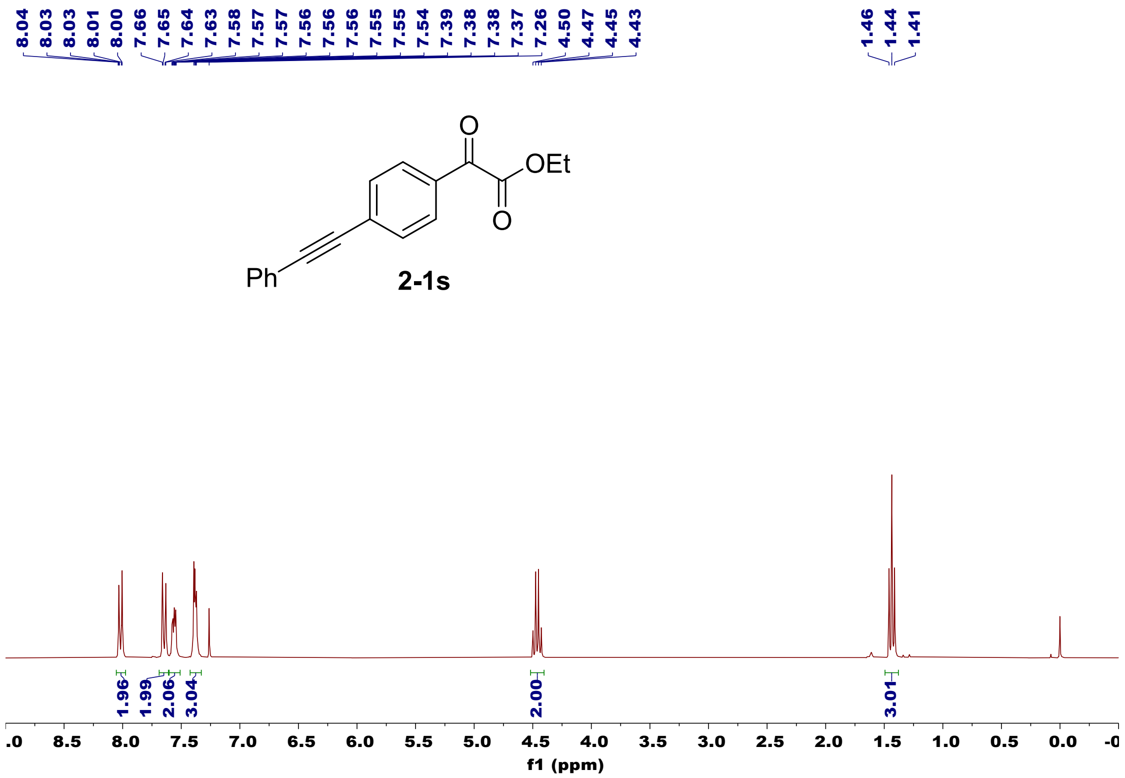
8.04 8.03 8.03 8.01 8.00 7.66 7.65 7.64 7.63 7.58 7.57 7.57 7.56 7.56 7.56 7.55 7.55 7.54 7.39 7.38 7.38 7.37 7.26 4.50 4.47 4.45 4.43
1.46 1.44 1.41
O
OEt
O
Ph
2-1s
1.96 1.99 2.06 3.04
2.00
3.01
.0 8.5 8.0 7.5 7.0 6.5 6.0 5.5 5.0 4.5 4.0 3.5 3.0 2.5 2.0 1.5 1.0 0.5 0.0 -0
f1 (ppm)

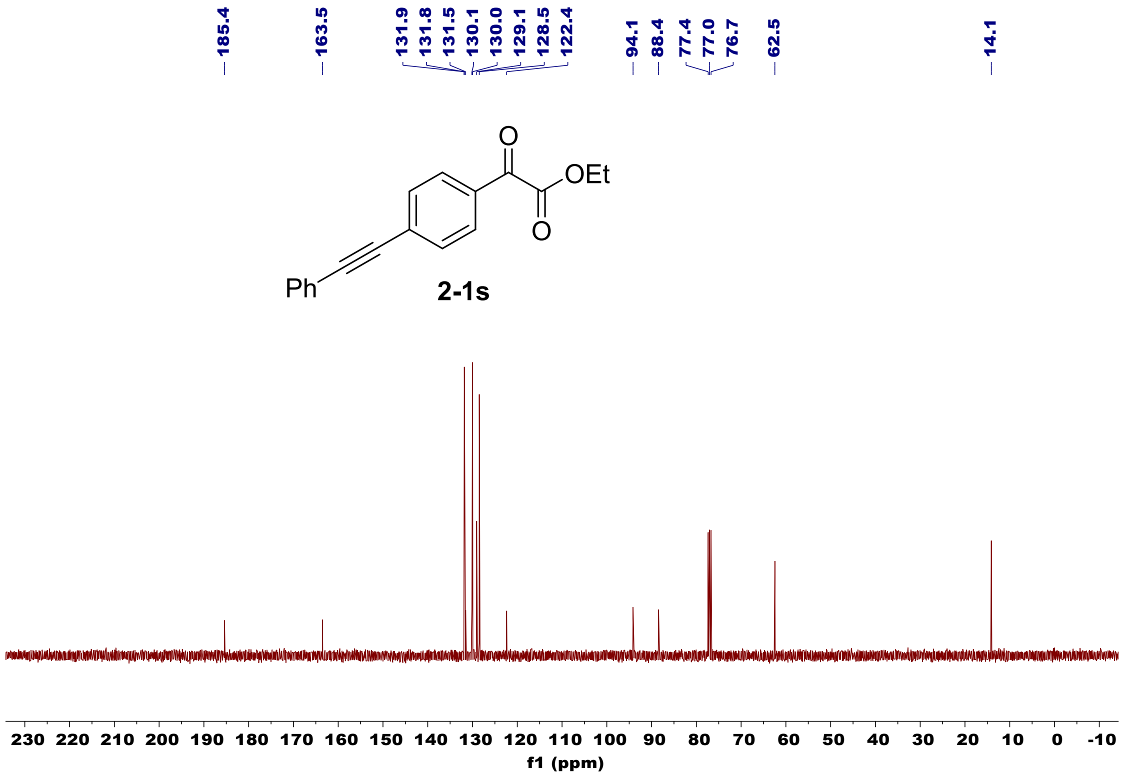
185.4
163.5
131.9 131.8 131.5 130.1 130.0 129.1 128.5 122.4
94.1 88.4
77.4 77.0 76.7
62.5
14.1
O
OEt
O
Ph
2-1s
230 220 210 200 190 180 170 160 150 140 130 120 110 100 90 80 70 60 50 40 30 20 10 0 -10
f1 (ppm)

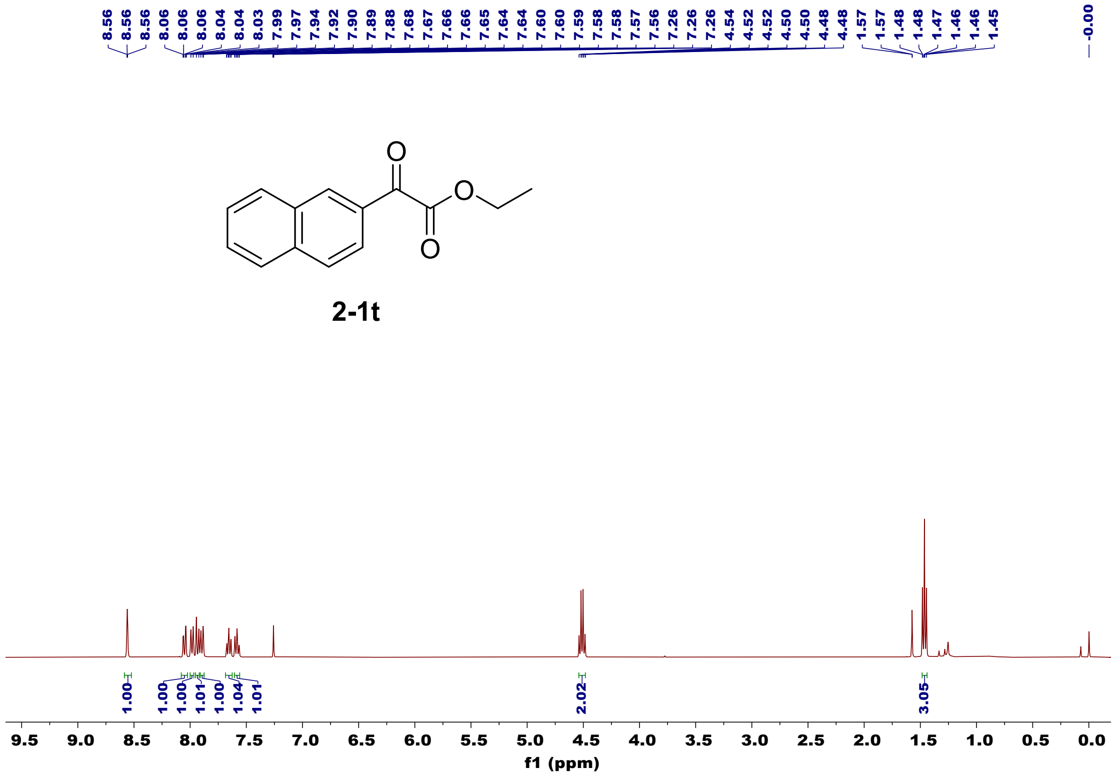
8.56 8.56 8.56 8.06 8.06 8.04 8.04 8.03 7.99 7.97 7.94 7.92 7.90 7.89 7.88 7.68 7.67 7.66 7.66 7.65 7.64 7.64 7.60 7.60 7.59 7.58 7.58 7.57 7.56 7.26 7.26 7.26 4.54 4.52 4.52 4.50 4.50 4.48 4.48 1.57 1.57 1.48 1.48 1.47 1.46 1.46 1.45
-0.00
O
O
O
2-1t
1.00 1.00 1.00 1.01 1.00 1.04 1.01 2.02 3.05
9.5 9.0 8.5 8.0 7.5 7.0 6.5 6.0 5.5 5.0 4.5 4.0 3.5 3.0 2.5 2.0 1.5 1.0 0.5 0.0
f1 (ppm)

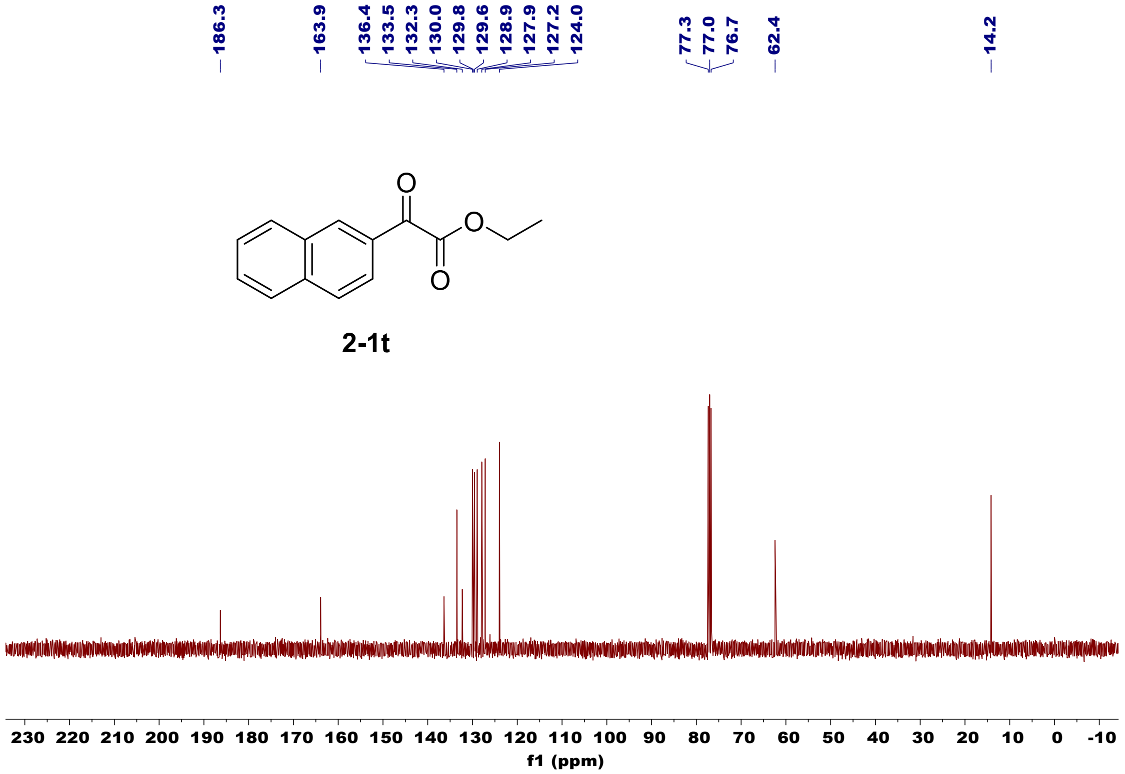
186.3
163.9
136.4 133.5 132.3 130.0 129.8 129.6 128.9 127.9 127.2 124.0
77.3 77.0 76.7
62.4
14.2
O
O
O
2-1t
230 220 210 200 190 180 170 160 150 140 130 120 110 100 90 80 70 60 50 40 30 20 10 0 -10
f1 (ppm)

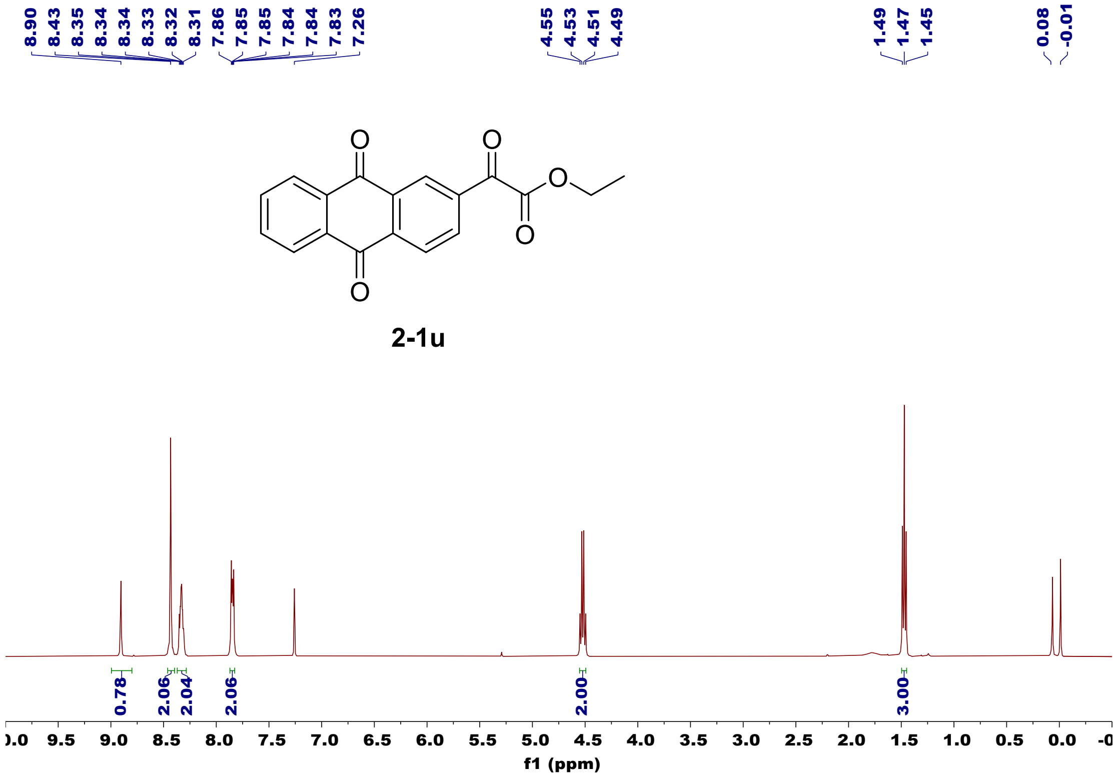
8.90 8.43 8.35 8.34 8.34 8.33 8.32 8.31 7.86 7.85 7.85 7.84 7.84 7.83 7.26
4.55 4.53 4.51 4.49
1.49 1.47 1.45
0.08 -0.01
2-1u
0.78 2.06 2.04 2.06 2.00 3.00
f1 (ppm)

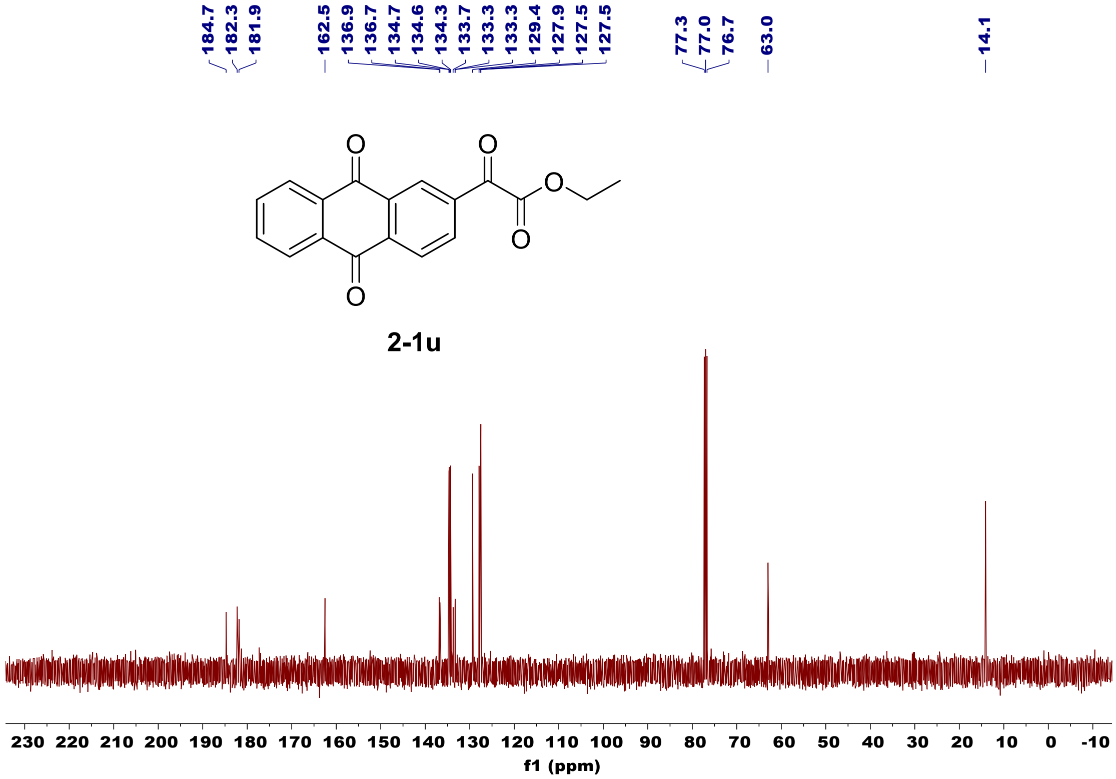
184.7 182.3 181.9
162.5 136.9 136.7 134.7 134.6 134.3 133.7 133.3 133.3 129.4 127.9 127.5 127.5
77.3 77.0 76.7
63.0
14.1
2-1u
f1 (ppm)

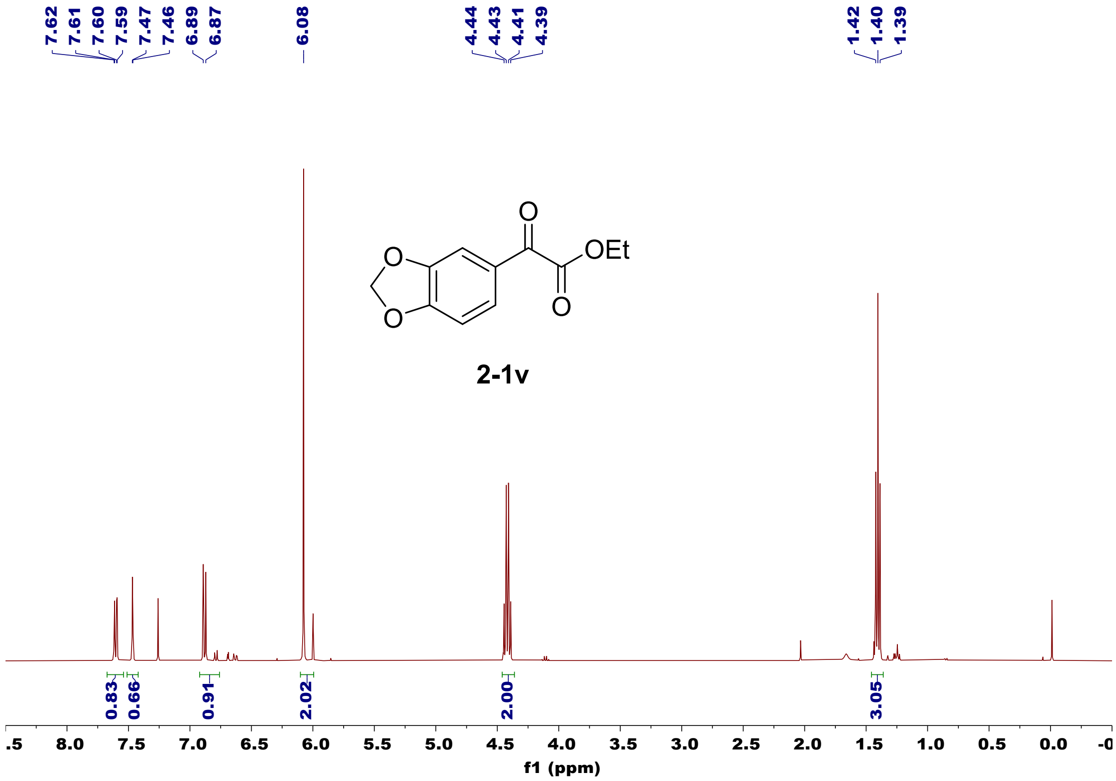

7.62
7.61
7.60
7.59
7.47
7.46
6.89
6.87
6.08
4.44
4.43
4.41
4.39
1.42
1.40
1.39
O
OEt
O
O
O
2-1v
0.83
0.66
0.91
2.02
2.00
3.05
f1 (ppm)

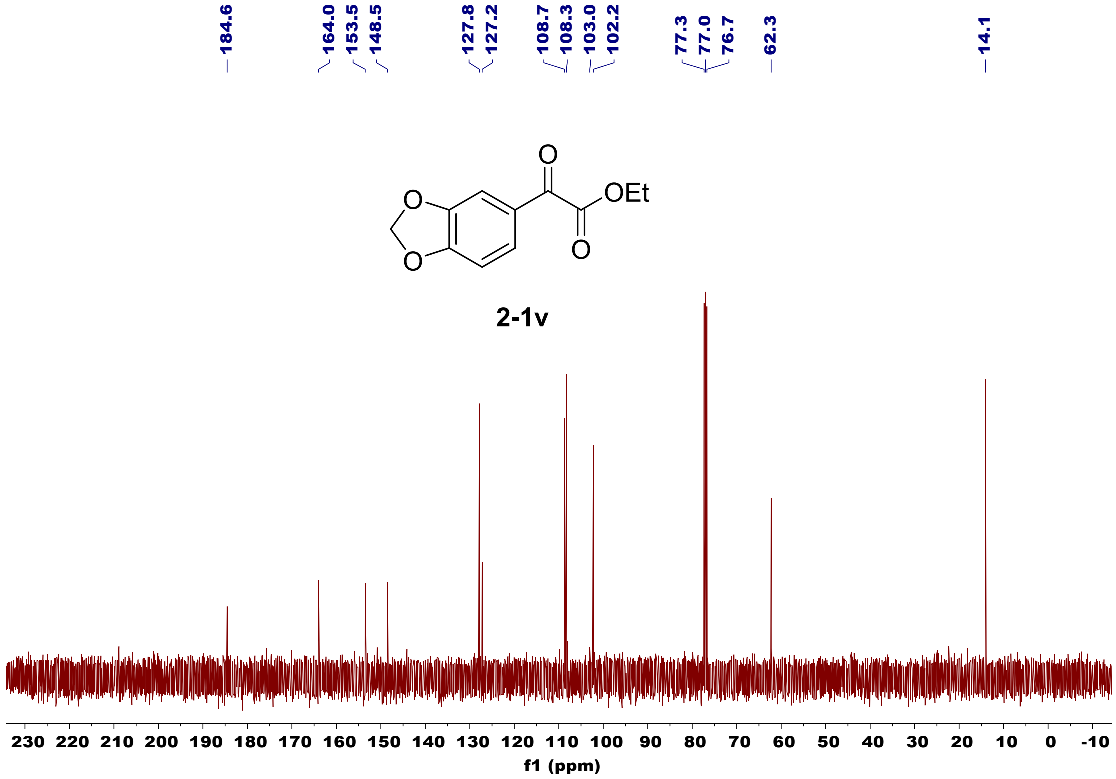

184.6
164.0
153.5
148.5
127.8
127.2
108.7
108.3
103.0
102.2
77.3
77.0
76.7
62.3
14.1
O
OEt
O
O
O
2-1v
f1 (ppm)

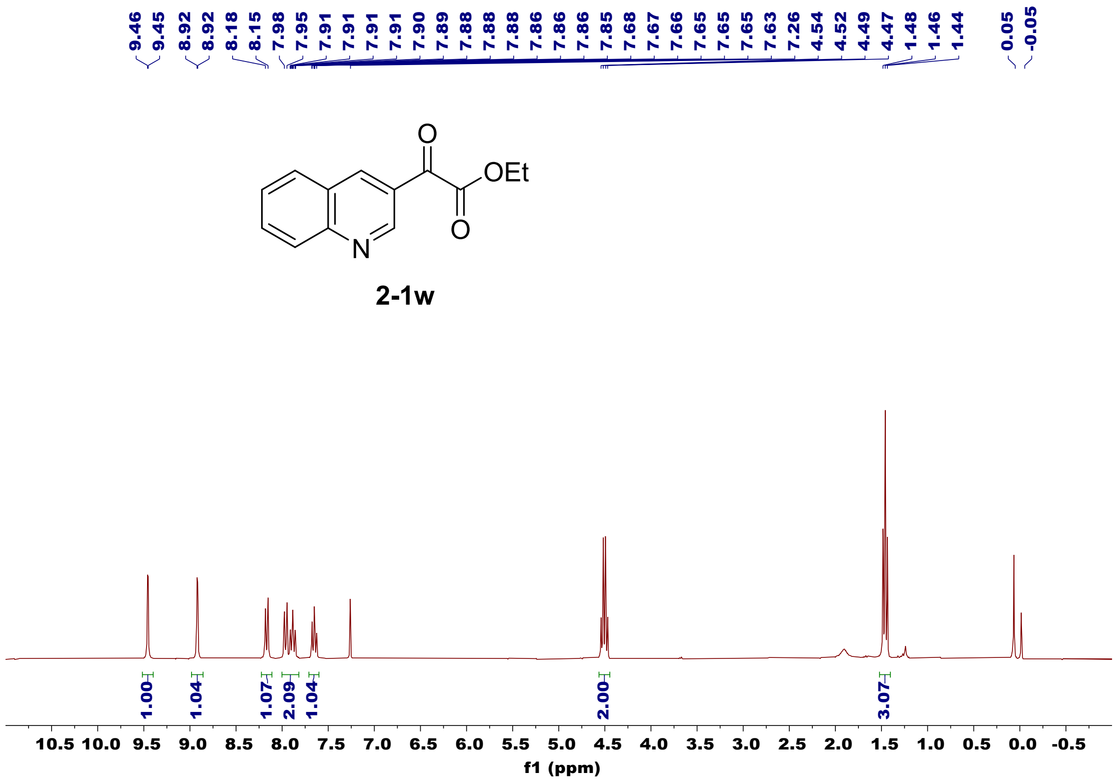
9.46 9.45 8.92 8.92 8.18 8.15 7.98 7.95 7.91 7.91 7.91 7.91 7.90 7.89 7.88 7.88 7.88 7.86 7.86 7.86 7.85 7.68 7.67 7.66 7.65 7.65 7.65 7.63 7.26 4.54 4.52 4.49 4.47 1.48 1.46 1.44 0.05 -0.05
O
OEt
O
N
2-1w
1.00 1.04 1.07 2.09 1.04 2.00 3.07
10.5 10.0 9.5 9.0 8.5 8.0 7.5 7.0 6.5 6.0 5.5 5.0 4.5 4.0 3.5 3.0 2.5 2.0 1.5 1.0 0.5 0.0 -0.5
f1 (ppm)

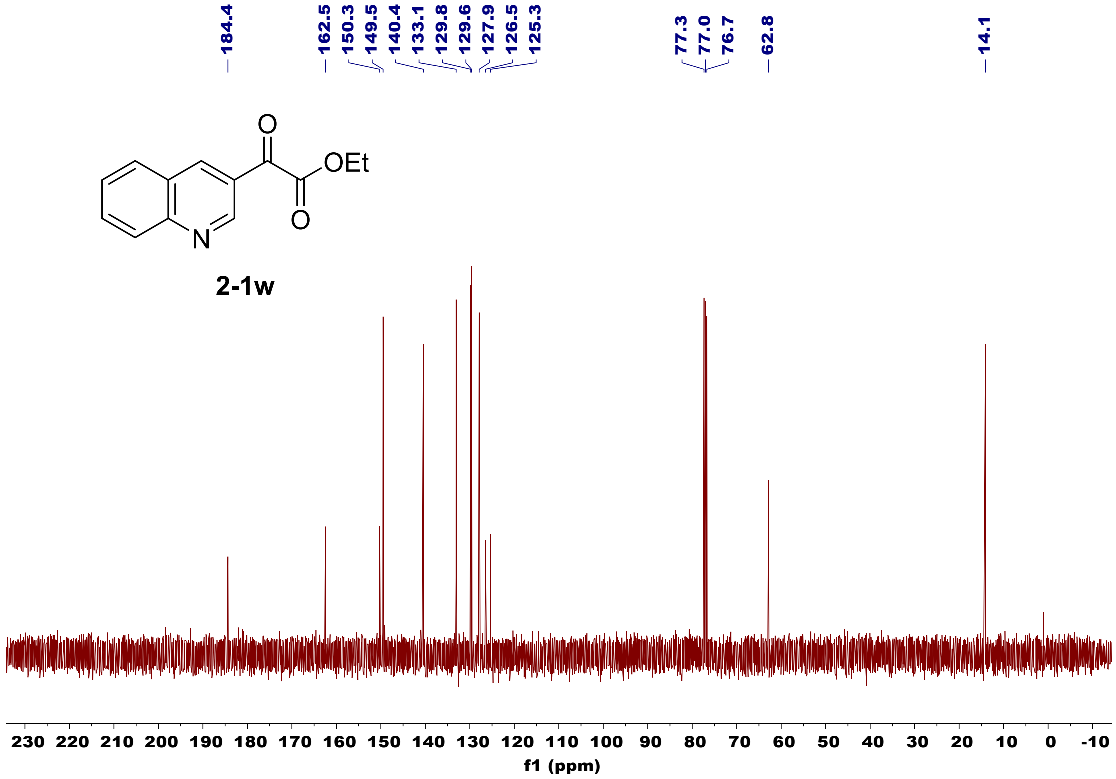
184.4 162.5 150.3 149.5 140.4 133.1 129.8 129.6 127.9 126.5 125.3 77.3 77.0 76.7 62.8 14.1
O
OEt
O
N
2-1w
230 220 210 200 190 180 170 160 150 140 130 120 110 100 90 80 70 60 50 40 30 20 10 0 -10
f1 (ppm)

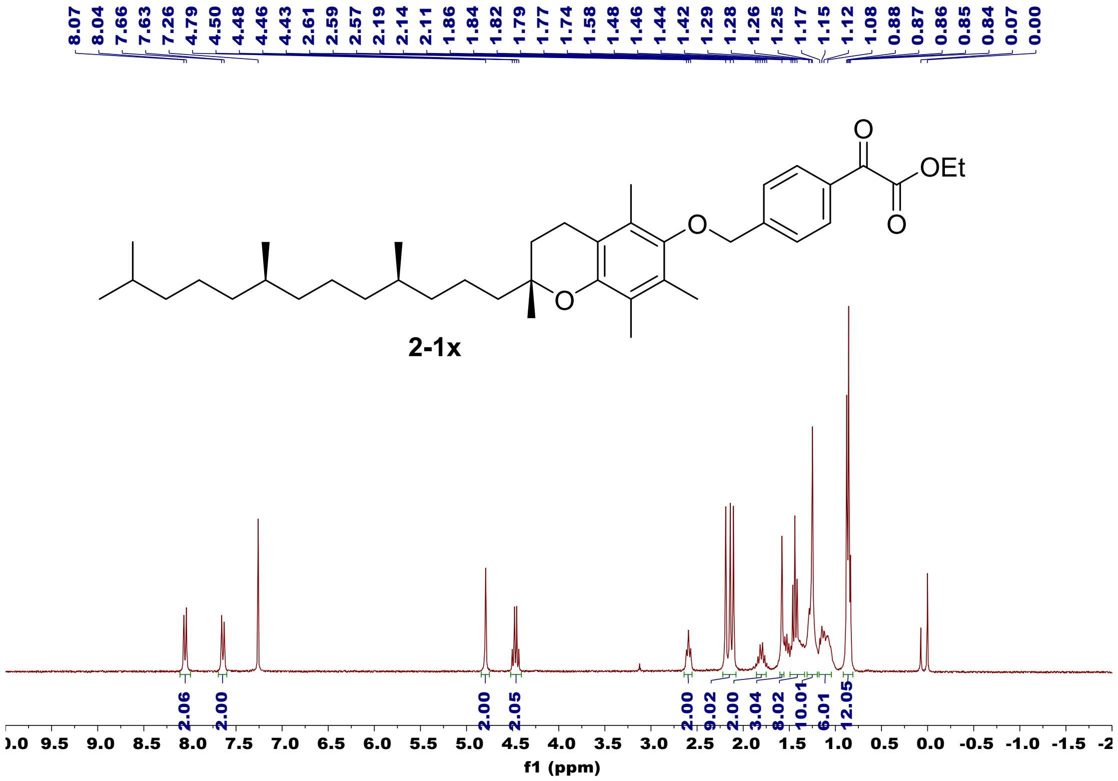
8.07 8.04 7.66 7.63 7.26 4.79 4.50 4.48 4.46 4.43 2.61 2.59 2.57 2.19 2.14 2.11 1.86 1.84 1.82 1.79 1.77 1.74 1.58 1.48 1.46 1.44 1.42 1.29 1.28 1.26 1.25 1.17 1.15 1.12 1.08 0.88 0.87 0.86 0.85 0.84 0.07 0.00
O
OEt
O
O
O
2-1x
2.06
2.00
2.00
2.05
2.00
9.02
2.00
3.04
8.02
10.01
6.01
12.05
f1 (ppm)

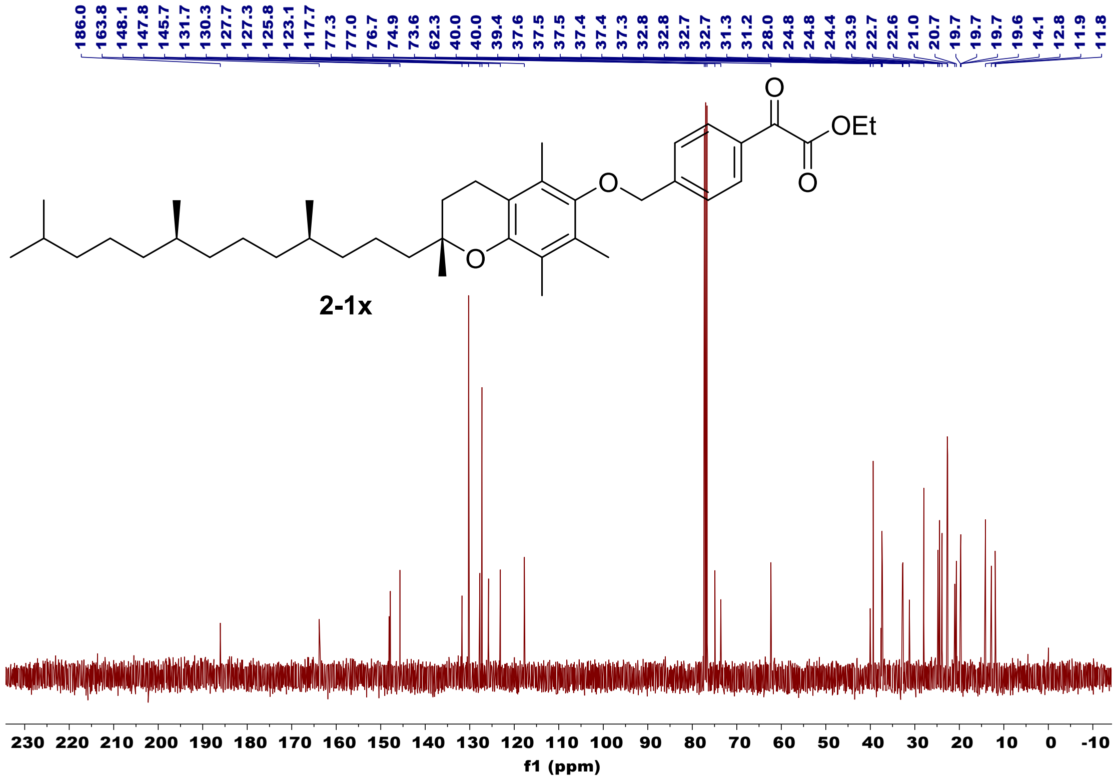
186.0 163.8 148.1 147.8 145.7 131.7 130.3 127.7 127.3 125.8 123.1 117.7 77.3 77.0 76.7 74.9 73.6 62.3 40.0 40.0 39.4 37.6 37.5 37.5 37.4 37.4 37.3 32.8 32.8 32.7 32.7 31.3 31.2 28.0 24.8 24.8 24.4 23.9 22.7 22.6 21.0 20.7 19.7 19.7 19.7 19.6 14.1 12.8 11.9 11.8
O
OEt
O
O
O
2-1x
230 220 210 200 190 180 170 160 150 140 130 120 110 100 90 80 70 60 50 40 30 20 10 0 -10
f1 (ppm)

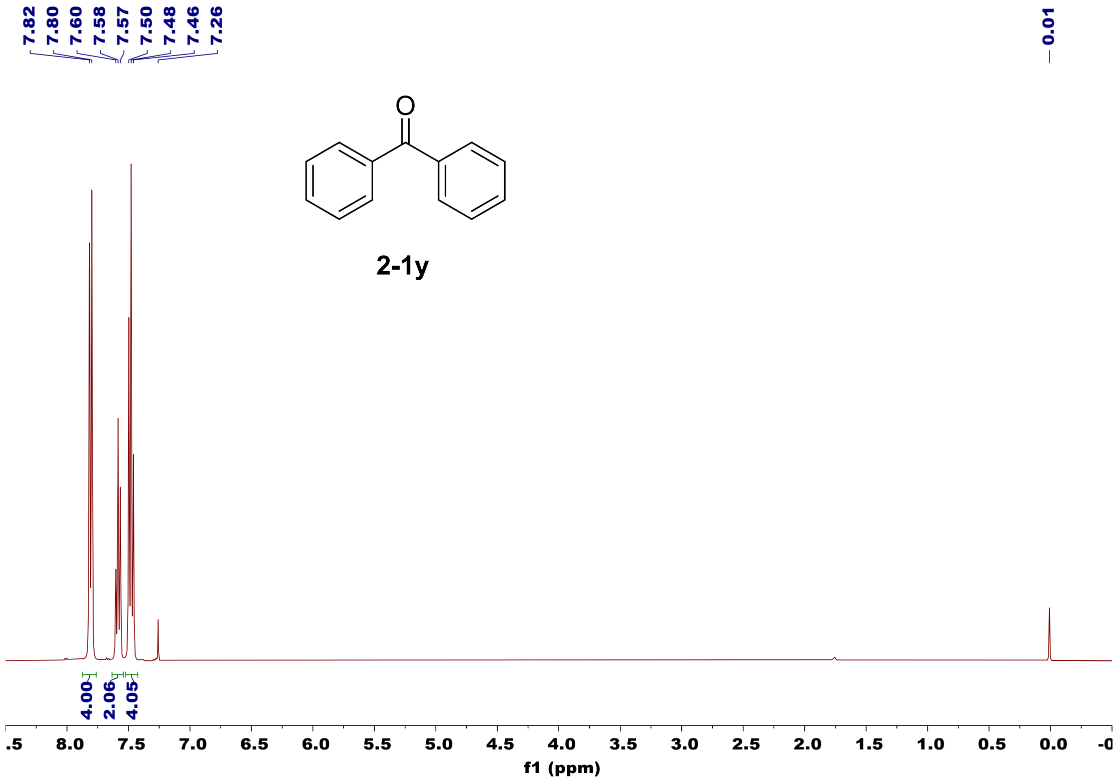
7.82
7.80
7.60
7.58
7.57
7.50
7.48
7.46
7.26
0.01
2-1y
4.00
2.06
4.05
f1 (ppm)

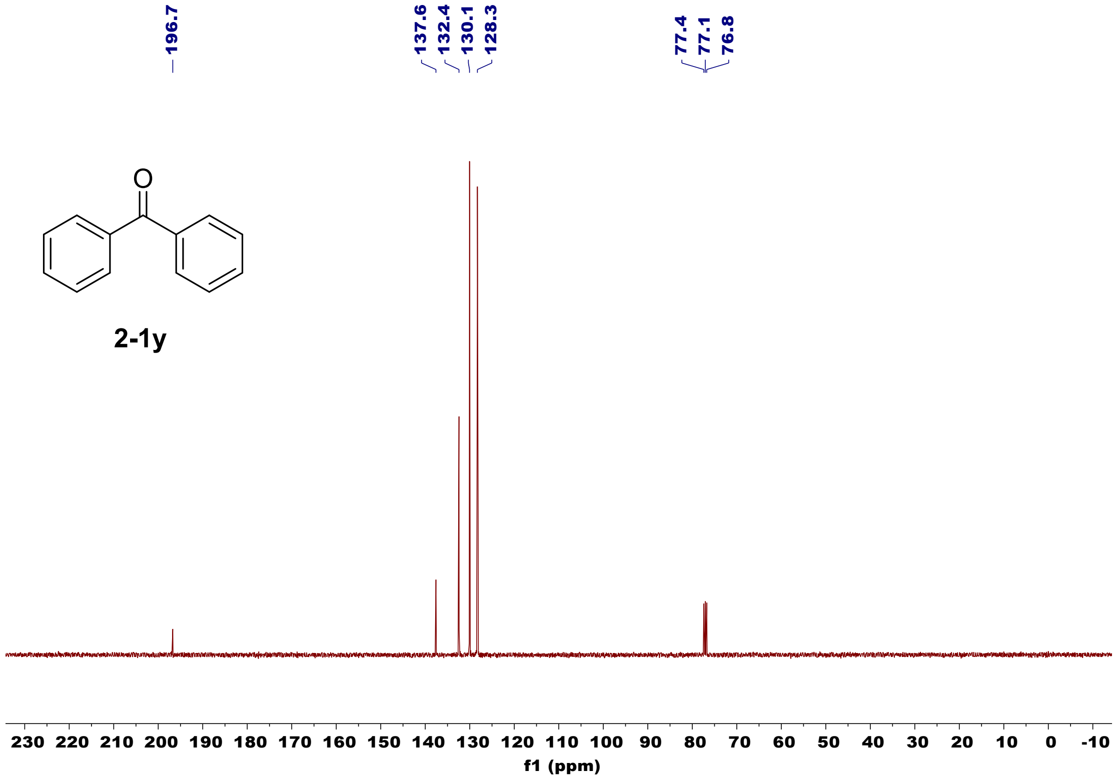
196.7
137.6
132.4
130.1
128.3
77.4
77.1
76.8
2-1y
f1 (ppm)

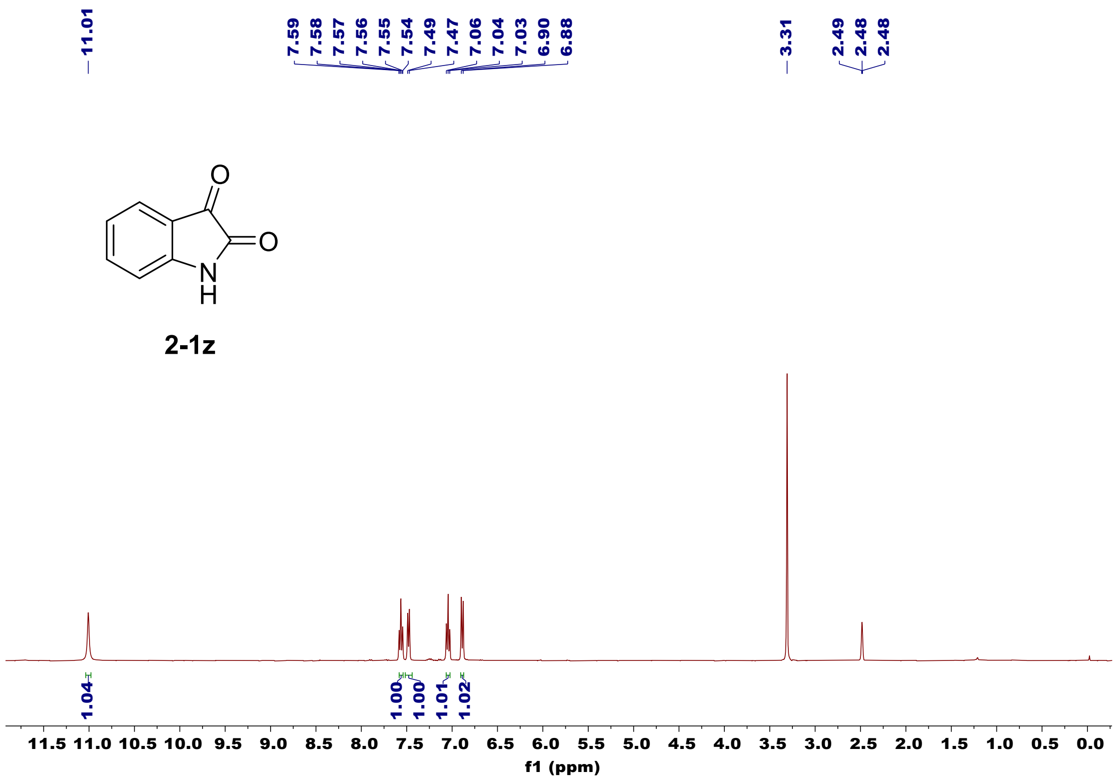
11.01
7.59 7.58 7.57 7.56 7.55 7.54 7.49 7.47 7.06 7.04 7.03 6.90 6.88
3.31
2.49 2.48 2.48
O
O
N
H
2-1z
1.04
1.00 1.00 1.01 1.02
11.5 11.0 10.5 10.0 9.5 9.0 8.5 8.0 7.5 7.0 6.5 6.0 5.5 5.0 4.5 4.0 3.5 3.0 2.5 2.0 1.5 1.0 0.5 0.0
f1 (ppm)

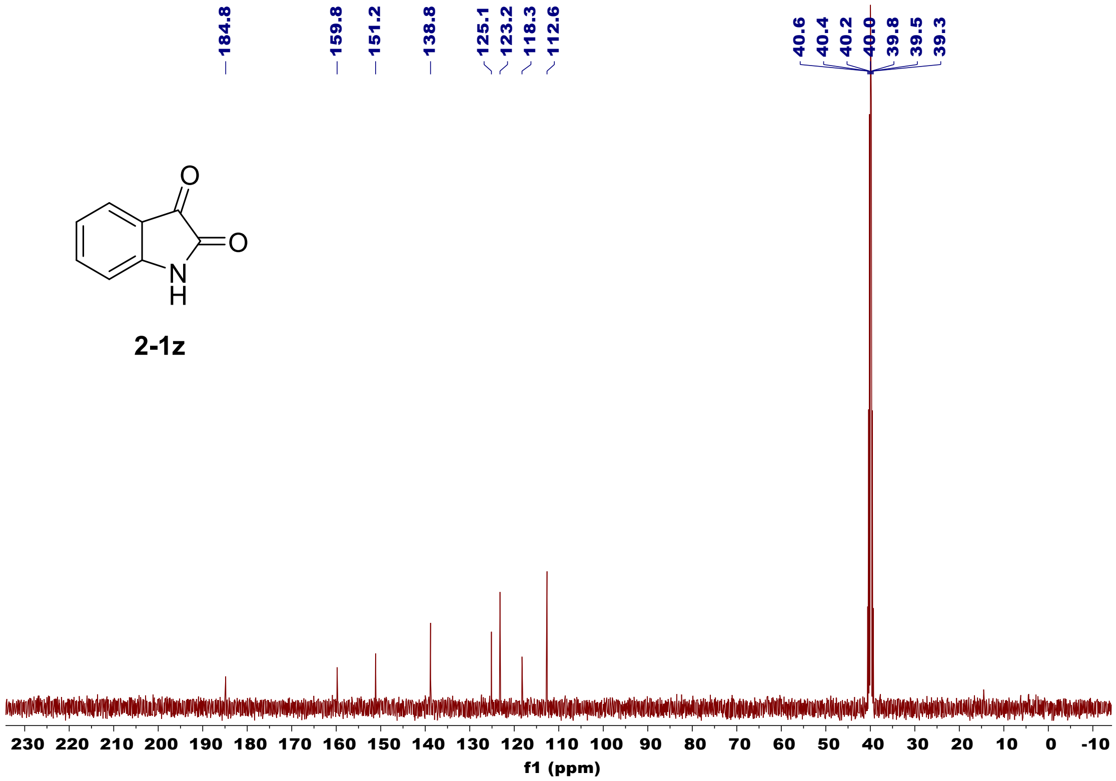
184.8
159.8 151.2 138.8 125.1 123.2 118.3 112.6
40.6 40.4 40.2 40.0 39.8 39.5 39.3
O
O
N
H
2-1z
230 220 210 200 190 180 170 160 150 140 130 120 110 100 90 80 70 60 50 40 30 20 10 0 -10
f1 (ppm)

6 HRMS of $^{18}O_2$ control experiment

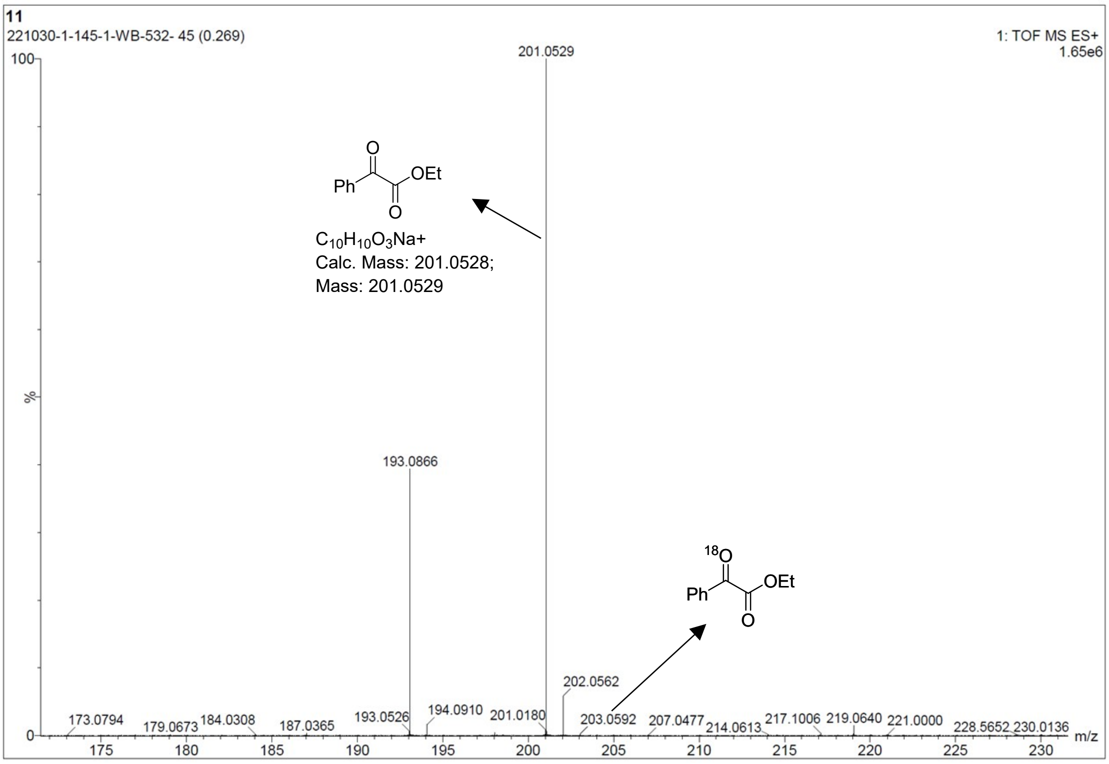

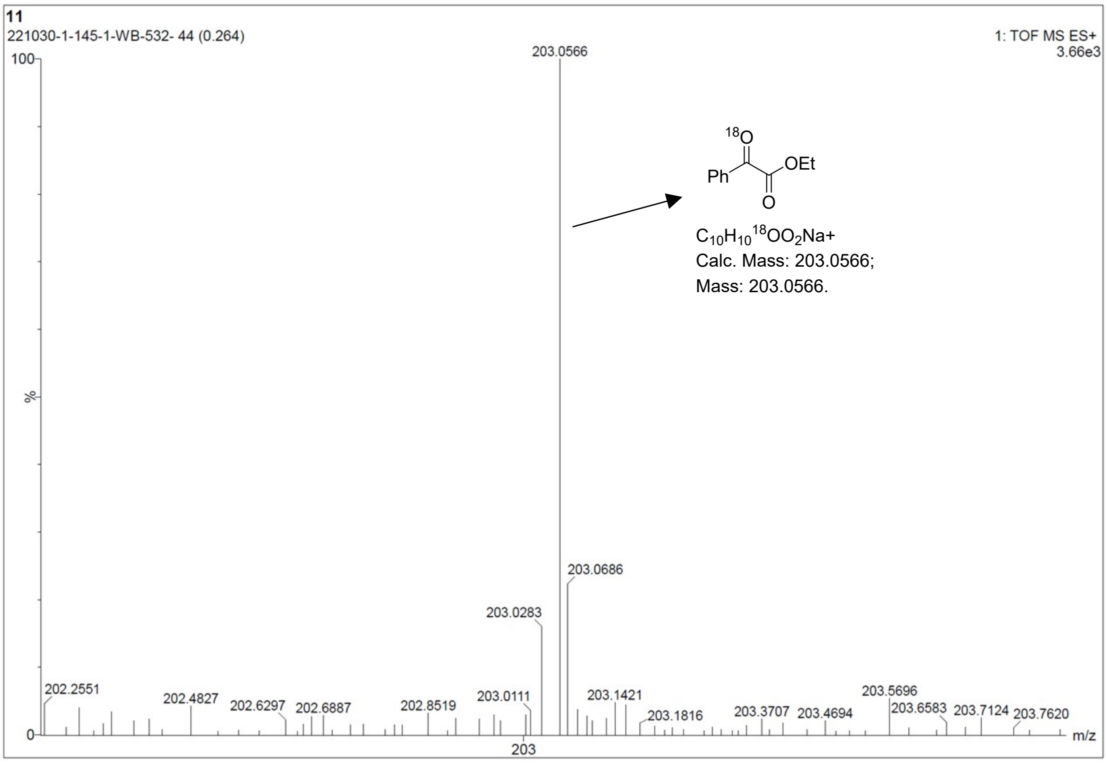

Supplement: RA-012-D2RA05739E-s001 [file RA-012-D2RA05739E-s001.pdf]
